# MOF-ChemUnity: Literature-Informed Large Language Models for Metal–Organic Framework Research

**DOI:** 10.1021/jacs.5c11789

**Published:** 2025-11-10

**Authors:** Thomas Michael Pruyn, Amro Aswad, Sartaaj Takrim Khan, Ju Huang, Robert Black, Seyed Mohamad Moosavi

**Affiliations:** † Chemical Engineering & Applied Chemistry, 7938 University of Toronto, Toronto, Ontario M5S 3E5, Canada; ‡ Clean Energy Innovation Research Centre (CEI), 6356 National Research Council Canada, Mississauga, Ontario L5K 1B4, Canada; ¶ Vector Institute for Artificial Intelligence, Toronto, Ontario M5G 0C6, Canada

## Abstract

Artificial intelligence (AI) is transforming research in metal–organic frameworks (MOFs), where models trained on structured computational data routinely predict new materials and optimize their properties. This raises a central question: What if we could leverage the full breadth of MOF knowledge, not just structured data sets, but also the scientific literature? For researchers, the literature remains the primary source of knowledge, yet much of its content, including experimental data and expert insight, remains underutilized by AI systems. We introduce MOF-ChemUnity, a structured, extensible, and scalable knowledge graph that unifies MOF data by linking literature-derived insights to crystal structures and computational data sets. By disambiguating MOF names in the literature and connecting them to crystal structures in the Cambridge Structural Database, MOF-ChemUnity unifies experimental and computational sources and enables cross-document knowledge extraction and linking. We showcase how this enables multiproperty machine learning across simulated and experimental data, compilation of complete synthesis records for individual compounds by aggregating information across multiple publications, and expert-guided materials recommendations via structure-based machine learning descriptors for pore geometry and chemistry. When used as a knowledge source to augment large language models (LLMs), MOF-ChemUnity enables a literature-informed AI assistant that operates over the full scope of MOF knowledge. Expert evaluations show improved accuracy, interpretability, and trustworthiness across tasks such as retrieval, inference of structure–property relationships, and materials recommendation, outperforming standard LLMs. This work lays the foundation for literature-informed materials discovery, enabling both scientists and AI systems to reason over the full existing knowledge in a new way.

## Graphical abstract

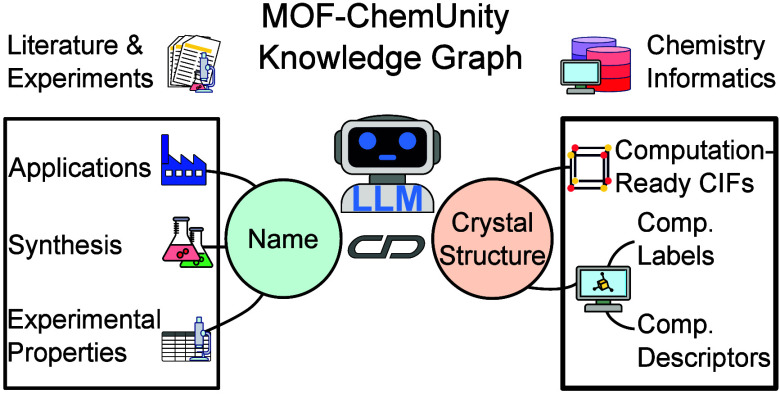

## Introduction

Metal–organic frameworks (MOFs) are a versatile class of crystalline materials characterized by high surface area, chemical tunability, and structural diversity, with potential applications spanning gas separation and storage, catalysis, and sensing.^1^ Since the discovery of MOFs with permanent porosity,^2,3^ research activity in the field has expanded rapidly, leading to the synthesis of over 125,000 distinct frameworks and the computational prediction of millions more.^4,5^ To navigate this vast and complex design space, machine learning has emerged as a powerful tool that leverages patterns in large chemical data sets to guide materials discovery.^6–10^ Models trained on structured data from computational chemistry have enabled rapid screening of MOFs for gas uptake and selectivity,^11^ mechanical strength,^12^ and other properties.^13,14^ However, this data represents only a fraction of the knowledge available: the majority of MOF-related information and data, especially from experimental studies, remains locked in unstructured literature and underutilized by machine learning methods. Structuring this wealth of information could vastly expand the scope and applicability of data-driven materials design.

Prior work has demonstrated the value of literature-derived data for MOF property prediction.^15–18^ For example, Nandy et al.^15^ applied natural language processing to extract thermal stability data from MOF literature and train predictive models. Other examples show the usefulness of such an approach for predicting surface area,^19^ recommending synthesis conditions,^20^ and designing stable MOFs.^21–24^ These efforts highlight the potential of text mining to convert textual knowledge into structured data sets for machine learning. Most existing approaches, however, remain narrow in scope, focusing on single-property extraction or static data sets that are not easily extendable. Even large-scale text-mined data sets emphasize property extraction from the literature rather than robust linkage to crystal structures.^19^ A major barrier to such unification is the lack of standardized naming conventions.^25–27^ For instance, a single compound can be referred to as “HKUST-1” in the literature, labeled “Compound 1” in a given article, and cataloged as “FIQCEN” in the Cambridge Structural Database (CSD).^28^ This inconsistency is not unique to MOFs but pervasive across materials science, leading to difficulties for both humans and LLMs in matching data across sources.^29^ Without robust entity resolution, linking experimental data to computational models remains a persistent challenge. Recent advances in large language models (LLMs) offer a path forward.^30^ LLMs enable general-purpose, scalable extraction of properties, synthesis procedures, and application insights directly from text.^31–34^ Crucially, they also allow for contextual inference, identifying properties, connecting entities, or recommendations even when not explicitly stated.^35^ These capabilities open new opportunities to bridge experimental observations and computational representations, unlocking the full potential of integrated, literature-informed materials discovery.

In this work, we develop a methodology that leverages LLMs to establish reliable one-to-one mappings between MOF names and coreferences used in a publication and crystal structures cataloged in the CSD to disambiguate MOF names and synonyms with their crystal structures. This linking enables the unification of experimental and computational data sources to create MOF-ChemUnity, a structured, and extensible knowledge graph that consolidates MOF-related data across the scientific literature. In its current version, MOF-ChemUnity integrates information from approximately 10,000 scientific articles and over 15,000 CSD crystal structures with computational chemistry properties into a machine-actionable format. Starting from crystal structures and linking them to the literature allows us to enrich computation-ready databases with experimental measurements, synthesis conditions, and expert insights. This crystal structure-centric design makes the data set directly usable for machine learning, since descriptors are defined on crystal structures, and it also supports continuous growth by incorporating new computational and experimental information as it becomes available. Finally, organizing this information in a knowledge graph enables the storage of contextual metadata, such as the source and justification for each extracted data point, thereby increasing transparency and trust and allowing both humans and AI systems to assess the validity and fidelity of the data.

We showcase several use cases, including multiproperty machine learning models that combine simulated and experimental data, expert-guided material recommendation using structure-based embeddings (machine learning descriptors quantifying MOF chemistry and geometry), and a literature-informed AI assistant capable of reasoning and evidence-grounded retrieval, and also cross-document extraction and data aggregation. The infrastructure is designed for continuous expansion, allowing for the incorporation of newly published MOFs and ongoing enrichment of the database. By organizing both extracted and inferred knowledge in a unified graph, MOF-ChemUnity provides a scalable, extensible, and queryable foundation for application-driven, literature-informed materials discovery.

## Results and Discussion

### LLM Agent and Workflows for Entity Resolution and Information Extraction

MOF synthetic chemists commonly report characterization data and property measurements in their research articles, referring to their materials using a variety of naming conventions, such as names derived from authors institutions (e.g., The Hong Kong University of Science and Technology, “HKUST-1”^3^), compound coreference (e.g., “Compound 1”), chemical formula (e.g., “Cu-BTC”), or structure-specific labels (e.g., “MOF-199”), all of which may refer to the same compound. In addition, when a new MOF is reported, often its crystal structure is resolved via X-ray diffraction and deposited into the CSD, where it is assigned a reference code. These structures form the foundation of computation-ready data sets, such as the CoRE MOF,^36,37^ QMOF,^13,38^ MOSAEC,^39^ which enable the prediction of a wide range of properties using computational chemistry, including adsorption and electronic properties.

Although CSD reference codes link crystal structures with computational data sets, a major challenge remains in connecting the experimental literature to these structures by accurately disambiguating MOF names. The most direct link is the digital object identifier (DOI) of the publication in which a structure was reported in CSD. However, this link is often ambiguous since a single DOI may correspond to multiple CSD entries, and a single paper may refer to multiple MOFs, including ones which are not deposited into CSD. To resolve this ambiguity and establish a reliable one-to-one mapping between MOF names and CSD crystal structures, we developed an LLM-based matching agent that leverages both textual and structural information. Specifically, we employ LLMs in a Retrieval-Augmented Generation (RAG) framework,^40^ which combines the reasoning capabilities of LLMs with metadata and crystallographic details from the CSD, such as atom types, chemical formulas, symmetry information, and lattice parameters, to match crystal structures to the corresponding MOF names and coreferences in the associated publications (see methods section and Figure S.2). This agent enables one-to-one mapping of MOFs with their corresponding names, while also identifying and resolving coreferences to individual MOFs within each document (Figure 1).

**Figure 1:**
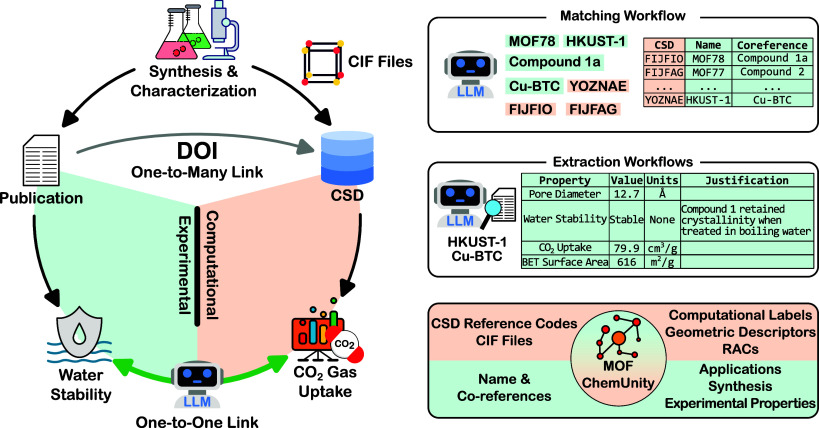
LLM agents for matching and extracting MOF data. The panel on the left illustrates the reporting process for MOFs. After synthesizing and characterizing the MOF crystals, the findings are reported as publications and crystal information is deposited as a CIF into Cambridge Structural Database (CSD).^28^ LLMs are used to create a one-to-one link between the results from experimental and computational efforts. On the right, the first workflow is matching a crystal structure, identified by a CSD reference code, to MOF names and coreferences in a research article. In the extraction workflow, the names are used to extract information from the publication like properties, applications, and synthesis procedures. Lastly, the MOF-ChemUnity knowledge graph is built combining computational and experimental information.

Once MOF entities are resolved, the next step is to extract relevant scientific information associated with each compound. For this, we developed two complementary extraction workflows: a general-purpose pipeline and a specialized workflow for nuanced properties, such as water stability. Both workflows employ RAG to extract data from scientific articles. The general workflow takes the resolved MOF name and its coreferences as input, identifies relevant sentences, and outputs structured data on material properties, synthesis procedures, and recommended applications. The specialized workflow targets specific queries and incorporates an additional verification step to improve robustness and accuracy, following our previous work using chain-of-verification (see methods section and Figure S.8).^35^

We performed systematic prompt tuning and benchmarking prior to large-scale deployment of these workflows. For the entity matching workflow, we evaluated 60 articles containing 108 MOFs, achieving to match over 98% of these MOFs with accuracy of 94%. For general information extraction, benchmarks were created for both property and synthesis data. The extraction of properties from 20 articles (90 properties) achieved precision 89% and yield 94%, while the synthesis procedure from 20 articles produced precision and yield 97.5%, correctly identifying precursors, solvents, conditions and summaries (see SI section 6). These results establish confidence in the scalability and applicability of workflows.

To build our data set, we start from MOF crystal structures from the CSD that are also included in computation-ready data sets, namely CoRE MOF 2019 and QMOF (see Figure S.22).^13,36^ This selection ensures that for these compounds, the crystal structures, computational data, and experimental reports are available. We retrieved the full-text publications for these MOFs from multiple publishers, including the American Chemical Society (ACS), Elsevier, and Royal Society of Chemistry (RSC) (see SI section 9). Applying the matching workflow successfully disambiguated and linked 93% of these MOF crystal structures, that is 15,143 crystal structures, with their names and coreferences across 9,874 papers (see Figure S.24). An example of this includes MOF structures reported in a single study,^41^ where our matching agent successfully matches the crystal structures in CSD (COKPEZ, COKMUM, COKMEW, COKPOJ, COKMOG, COKPUP) with the corresponding names and synonyms, namely M-MOF-74, or COP-27-M, or M2­(dobdc) (M = Mg, Mn, Fe, Co, Ni, Cu, Zn). We note that the coreferences can indicate when a MOF has undergone postsynthetic modifications —for example, when UiO-66 is loaded with platinum nanoparticles, it is referred to as “Pt@UiO-66”. The matching agent is able to successfully differentiate these MOFs from their standard, nonmodified MOF names. Similarly, the matching agent is also able to differentiate between isoreticular MOFs reported in the same work. We then applied our extraction workflows to these matched MOFs to retrieve information on synthesis procedures, reported properties, and application insights. While this work focuses on these three categories, the extraction workflows are readily extendable to other properties of interest to extract broader range of literature-derived information. Our extraction campaign yielded over 70,000 properties, such as thermal stability and emission wavelength, and 2,500 recommended applications, such as gas separation and storage and photoluminescence, for the matched MOFs (see Figure S.24).

A notable outcome of our analysis is the extent and diversity of MOF applications, with over 25 distinct application classes identified, ranging from sustainability applications (e.g., gas separation and storage, water purification, and pollutant removal), to health (e.g., drug delivery and cancer therapy), and electronics (e.g., sensing and LED) (Figure 2a). In addition, Figure 2b shows the range of properties reported in the literature for some of the applications. As we observe significant overlap across property categories and application domains, this highlights an opportunity: researchers working in one domain may benefit from insights reported in others. However, the breadth and volume of this information quickly become overwhelming to navigate manually, by human researchers and AI, motivating the need for a unified, structured framework to organize and cross-link relevant knowledge across disciplines.

**Figure 2:**
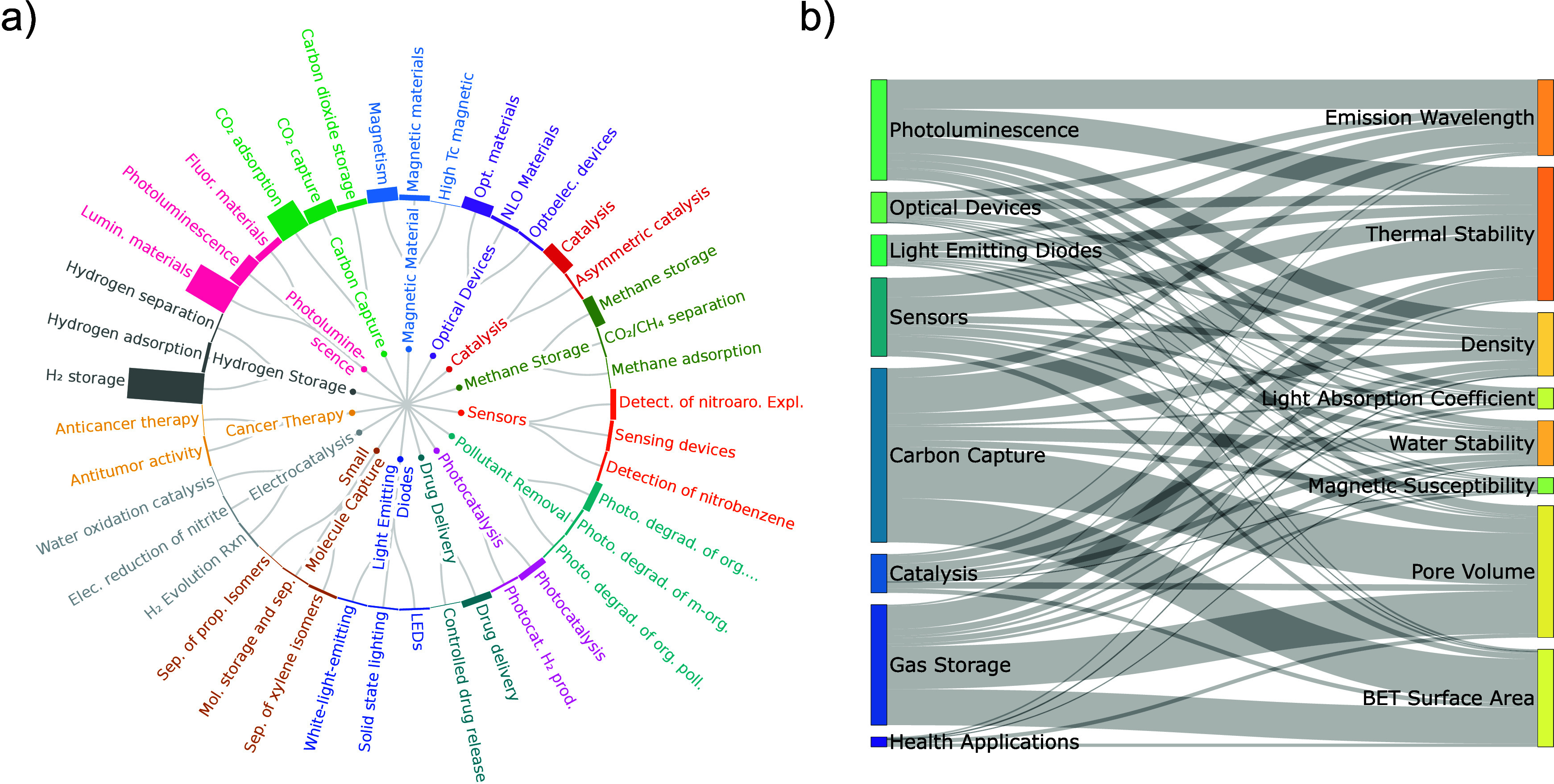
MOF applications and properties extracted from the literature. Over 70,000 properties and 2,500 application mentions were extracted for more than 15,000 MOF crystal structures by resolving MOF names and coreferences across approximately 10,000 publications. (a) Application taxonomy of MOFs, from the extracted suggested applications, where each branch represents a distinct application domain, and sub-branches reflect specific terms and phrases extracted from the text. Bar lengths correspond to the frequency of occurrence across the corpus. (b) Relationship map connecting MOF application domains (left) to relevant physicochemical properties (right). Edge thickness reflects the frequency with which a given property is reported for each application, illustrating how literature-reported measurements align with application-specific priorities.

### Structuring MOF Data into a Knowledge Graph

MOF chemical data is inherently heterogeneous, originating from experimental studies, computational databases, and a wide range of application domains. These data are reported using diverse terminologies, formats, and granularity, making it challenging to unify, analyze, or reuse at scale. To convert this fragmented knowledge into a resource that is both machine-readable and human-navigable, we organize all extracted and linked data in a knowledge graph, “MOF-ChemUnity”, which unifies MOF data across experiment and computation. Knowledge graphs are a powerful way to represent heterogeneous data^42,43^ where entities (nodes) are connected by labeled relationships (edges), allowing the representation of rich interlinked relationships that go beyond traditional tabular formats. While previous works also constructed knowledge graphs for MOFs (MOF-KG^42^) specifically and framework materials generally (KG-FM^43^), they face key limitations that MOF-ChemUnity addresses. MOF-KG is constructed using a combination of multiple existing structured databases.^42^ KG-FM extracts information from abstracts only and does not link extracted information to computational data like CoRE MOF 2019 or QMOF.^43^

In designing MOF-ChemUnity, we focused on three key objectives: scalability, linkability, and queryability. First, the knowledge graph must be scalable and appendable, allowing seamless integration of new data as the literature and computational databases continue to grow. Second, it must support cross-document entity resolution, ensuring that multiple references to the same compound, across different papers, naming conventions, or databases, can be accurately linked. Third, it should enable both local and global queries, supporting fine-grained lookups (e.g., synthesis conditions for a single MOF) as well as broader analyses (e.g., identifying structure–property trends across applications). To achieve these goals, we designed a schema with unique node and relationship types. Each MOF is represented as a MOF node, and all coreferences or synonyms are linked through “Name” nodes using “Has Name” relationships. Publications, synthesis steps, properties, and application mentions are modeled as distinct nodes, connected via semantic relationships such as “Has Property”, “Has Synthesis”, and “Has Source”. The resulting knowledge graph contains over 40,000 nodes and 3,200,000 relationships. The complete schema, full knowledge graph, and an individual MOF subgraph is shown in Figure 3.

**Figure 3:**
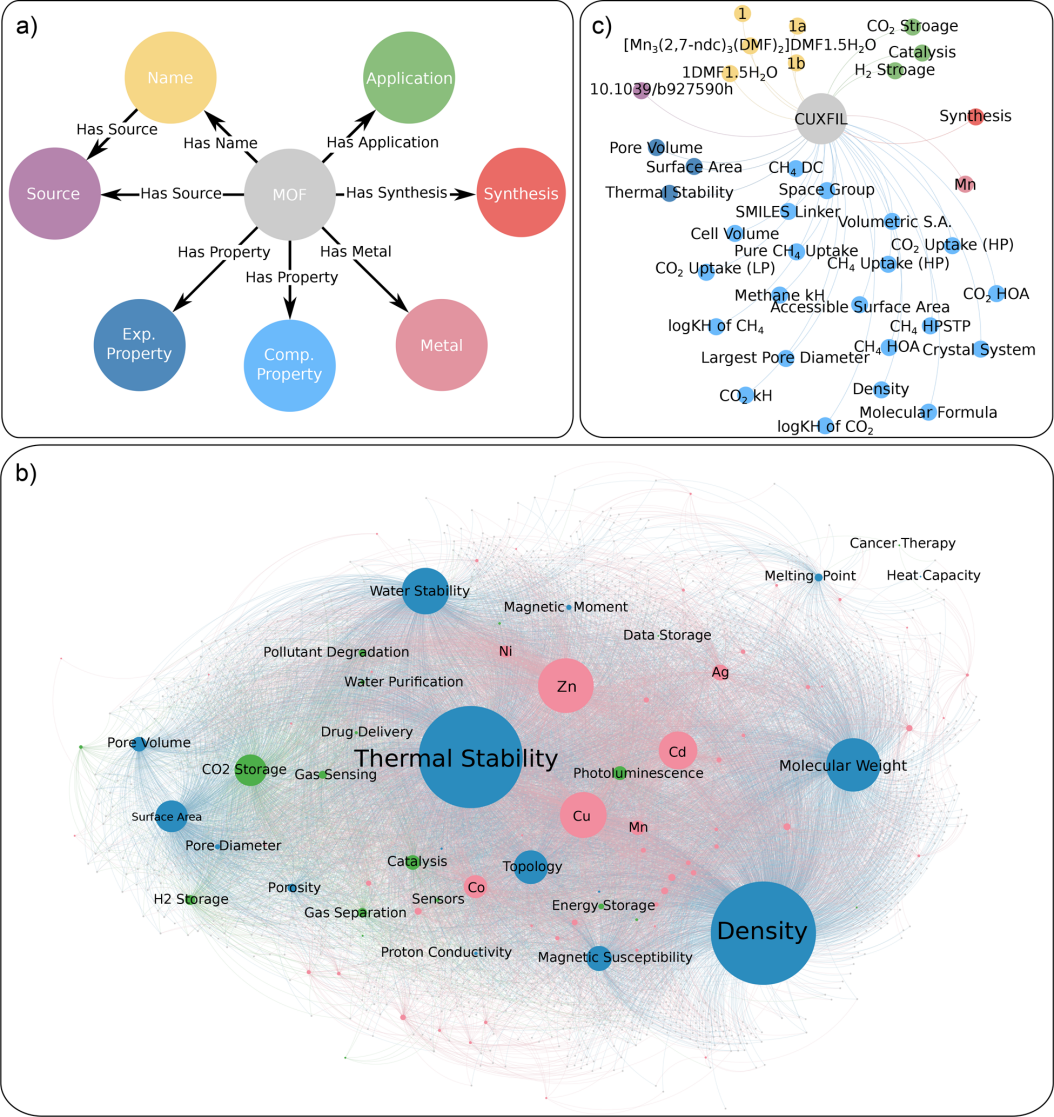
Structuring heterogeneous MOF data through knowledge graphs. (a) The MOF-ChemUnity schema, representing the unique node and relationship types contained in the knowledge graph. (b) The entire knowledge graph. In this representation, the nodes with the highest number of connections appear the largest. As well, nodes that are highly interconnected with each other appear closer together. Small gray nodes represent individual MOFs. This visualization was created using Gephi.^44^ (c) An example of an individual MOF’s subgraph, detailing its connections. The color coding shows the type of node from (a).

This relational framework captures the context and connectivity of scientific knowledge, what a material is used for, how it is made, where it was reported, and what properties it exhibits, mirroring how researchers reason across disciplines. It supports rich, multihop queries such as “Which MOFs reported for pollutant removal in aqueous environments have high water stability and use zirconium as the metal node?”, queries that resemble the way a human expert would search the literature. On the machine side, the graph structure also supports retrieval-augmented reasoning, allowing language models to generate context-aware answers grounded in structured relationships.^45^

A key advantage of using a knowledge graph is its support for storing textual data to improve transparency and explainability. In MOF-ChemUnity, evidence and reasoning behind each extracted relationship is preserved by storing the textual justifications from the LLM. These justifications are LLM-generated rationales or direct sentences from the paper which are directly stored on the knowledge graph. This is particularly useful when there is no standard reporting procedure followed for a property. For example, authors report the water stability of their synthesized materials in various ways, from evidence of insolubility to advanced characterizations. In MOF-ChemUnity, we store the full evidence from the paper in addition to the stable/unstable labels. As an example, Zn­(LTP)_2_ is flagged as water stable since the authors mention in their work that “the as-synthesized material is insoluble in water and common organic solvents”.^46^ While this might provide a weak evidence that the MOF is water stable, for practical applications, further investigation is needed. Storing the evidence together with the label allows users to make decisions regarding data quality. Moreover, this improves transparency and trust, enabling researchers to critically assess the provenance of each connection. Similarly, the knowledge graph allows the source of each computational property and label to be stored. This allows for the storage of computational settings, such as the choice of the force field and partial charge calculations, as well as the computation-ready crystal structure. This improves traceability and enables continuous updates to improve database sources.

### Multi-Property Machine Learning on Unified Experimental and Computational Data

A core use case of MOF-ChemUnity is to serve as a structured, machine learning ready data set for modeling structure–property relationships. Because all extracted data are linked to crystal structures, MOF-ChemUnity enables models to incorporate both computationally derived and experimentally reported properties within a unified framework. This integration allows researchers to move beyond computational data and supports more comprehensive MOF discovery tasks, such as multiproperty prediction and optimization.

As an illustrative example, we consider the discovery of MOFs for carbon capture, where ideal candidates must combine high CO_2_ uptake with robust water stability. While CO_2_ uptake can be estimated using molecular simulations, water stability is typically derived from experimental studies. Previous machine learning models have treated these properties separately. Although models for CO_2_ uptake are widely available, only recently have water stability models emerged.^23^ Most notably, Terrones et al.^22^ curated a data set of over 1,000 literature-derived labels and trained a predictive model. In comparison, MOF-ChemUnity contains over 1,800 water stability annotations, linked directly to crystal structures and additional properties.

Using the MOF-ChemUnity water stability data set, we trained a classifier model that achieves strong performance on water stability prediction, with 80% accuracy and an F1 score of 86% (Figure 4), with refinements to the model further explored in the SI (Section 13). Importantly, because MOF-ChemUnity also includes simulated CO_2_ uptake data from molecular simulations, we can perform joint filtering and identify materials that meet both criteria. In particular, we find MOFs with high predicted CO_2_ adsorption and high water stability (see SI section 14), where we perform density functional theory (DFT) and accurate calculations on the final identified set.

**Figure 4:**
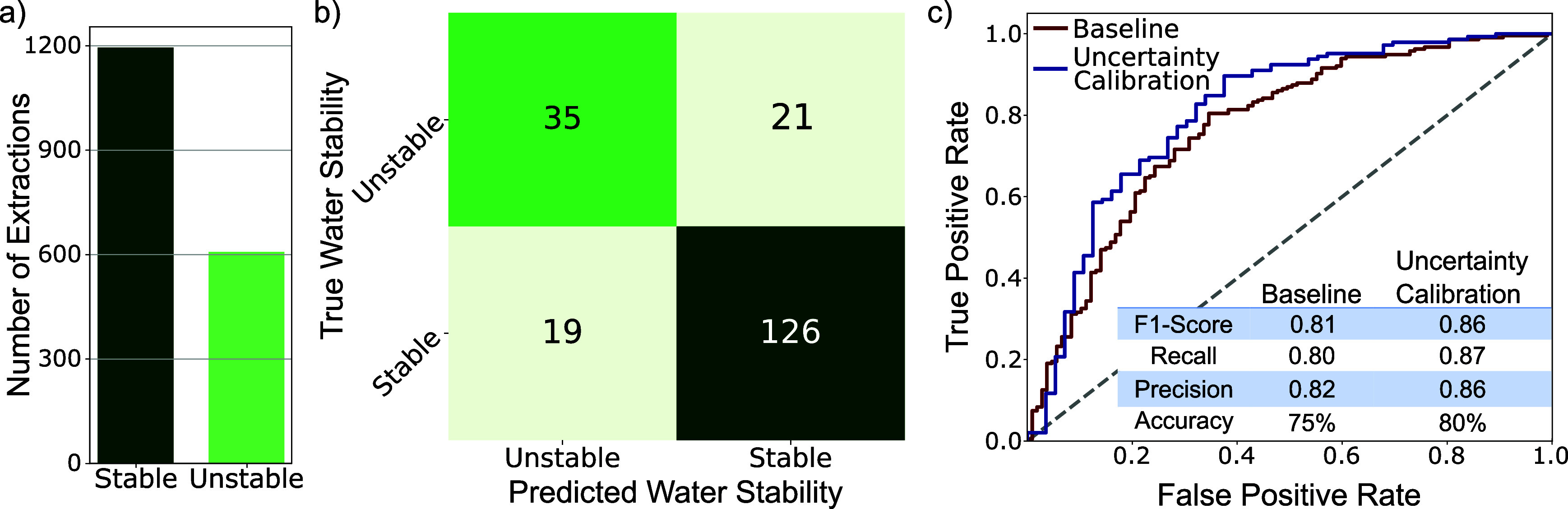
Using machine learning to predict MOF water stability. (a) Distribution of extracted water stability labels. (b) Confusion matrix showing water stability prediction results from using our machine learning model. (c) ROC-AUC curves, alongside their F1-scores, recall, precision and accuracy for baseline model and model with uncertain points removed.

In this demonstration, water stability was treated as a binary stable/unstable classification. This simplification does not capture important distinctions between liquid water and water vapor, nor does it reflect specific process conditions relevant to carbon capture. The current data set is based on heterogeneous literature sources and therefore provides only a proxy for practical stability. A more refined analysis for a specific process of interest, such as testing materials in vapor or steam will be essential. This example illustrates how our data set can already support multiproperty screening and highlights its potential to accelerate discovery workflows as more detailed experimental and computational standards emerge.

### Navigating MOF Chemical Space by Expert Recommendations

Experts often recommend their MOFs for specific applications based on intuition, experience, or domain knowledge. From the literature, we extracted hundreds of such recommendations for applications including gas separation, catalysis, and sensing (Figure 2c). These recommendations are typically grounded in comparisons with benchmark materials available at the time of discovery, drawing on prior studies to justify performance claims. While inherently valuable, these recommendations are often difficult to formalize or use systematically.

To address this, we used the link between expert recommendations and crystal structures in MOF-ChemUnity to embed MOFs into a structure-aware chemical space. Using machine learning descriptors derived from crystal structures, we generated low-dimensional embeddings and constructed a nearest-neighbor map that clusters materials with similar pore geometry and chemistry. The descriptors we use include numerical descriptors for pore geometry, such as pore size and surface area, and for chemistry using revised autocorrelation (RACs). In Figure 5a, we show that materials with similar pore volumes tend to cluster together. This allows us to explore the neighborhood of any expert-recommended MOF to identify structurally similar candidates, effectively translating human intuition into a reusable tool for materials recommendation.

**Figure 5:**
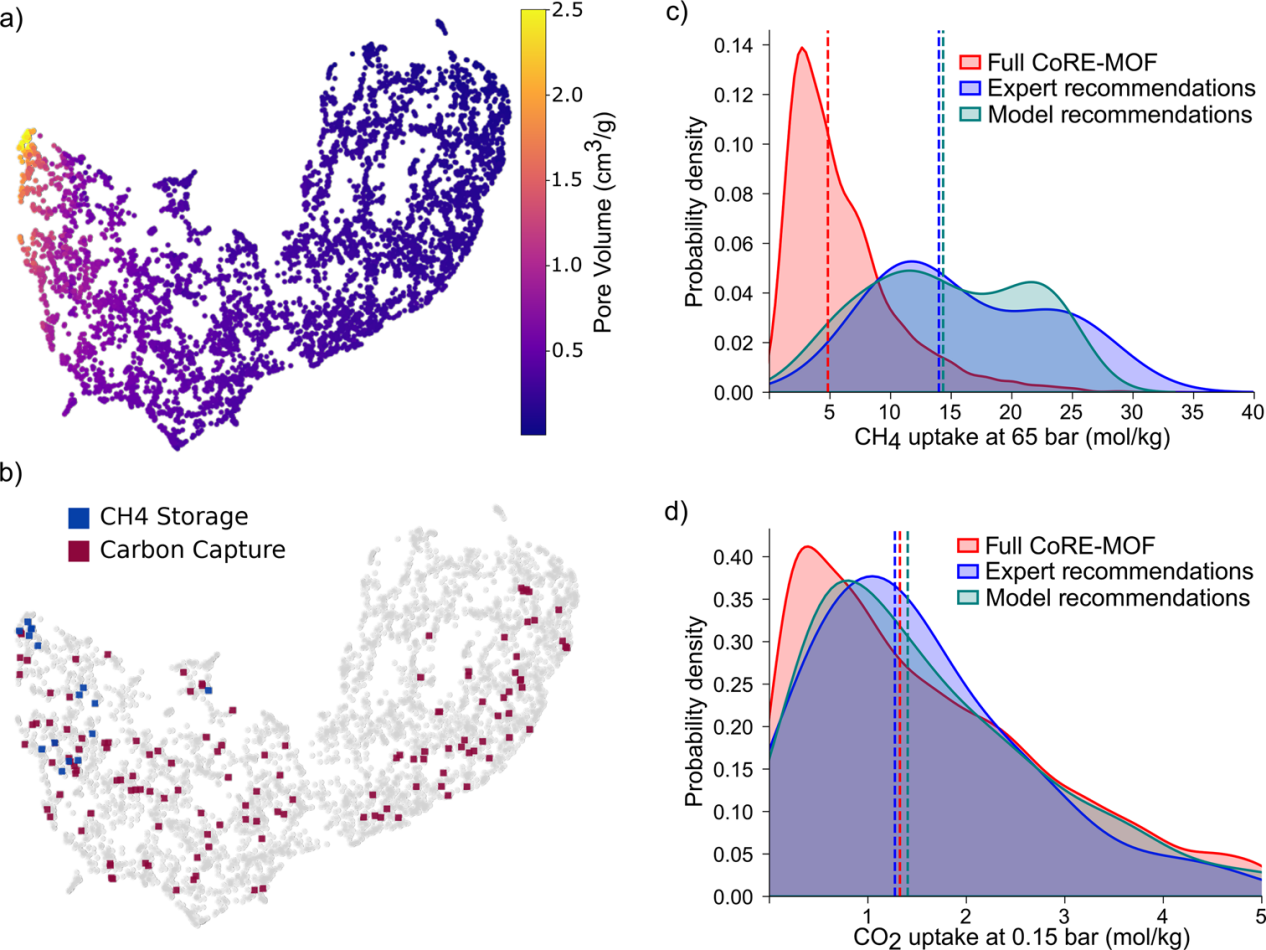
MOF applications recommendation by experts. (a and b) UMAP embedding of pore geometry descriptors, such as pore size, surface area, and pore volume,^47,48^ of MOFs in the MOF-ChemUnity database; in (a) color coding shows pore volume for each material, and (b) expert-recommended MOFs for methane storage and carbon capture. (c and d) Distribution of the methane and carbon dioxide uptake of all materials in the CoRE MOF 2019 database,^36^ MOFs expert recommended in the literature, and ones recommended by a nearest neighbor model trained on the computational features and expert recommendations.

We evaluated the effectiveness of this approach on two applications for which we have computational property data: methane storage and carbon dioxide capture. For each MOF recommended in the literature, we identified its location in the embedding space (Figure 5b) and sampled nearby materials. Figure 5c and d show that in both applications these neighboring MOFs, labeled as model recommendations, exhibit properties similar to those recommended by experts. This demonstrates that expert intuition, when mapped into structure space, enable machine learning models to learn their intuition and experimental data.

It is insightful to assess the strength and specificity of experts’ recommendations. For this, we compared the property distributions of expert-recommended MOFs to those of their neighbors and to randomly sampled materials from the full database. For methane storage, the average CH_4_ uptake of both expert-recommended and nearby MOFs is substantially higher than the data set-wide average (Figure 5c), indicating that experts are effectively selecting high-performing materials for methane storage. This aligns with prior work suggesting that methane storage is governed by relatively intuitive geometric properties, such as void fraction and working capacity under pressure swing conditions.^11^ In contrast, for carbon capture, the property distributions of expert-recommended MOFs are similar to those of random samples (Figure 5d), suggesting that expert intuition is less reliable in this domain. This limitation likely stems from two factors. First, evaluating performance in carbon capture is inherently more complex than in methane storage: high-performing materials can emerge from diverse and often nonintuitive combinations of properties, such as CO_2_ uptake, selectivity, and heat of adsorption. A MOF may be recommended based on the strength in one of these metrics, even if it is not optimal overall. Second, materials design itself is more challenging. The embedding locations of the MOFs recommended by experts show little clustering (Figure 5b), reflecting that carbon capture performance depends on a subtle interplay of pore geometry and chemical functionality rather than on a single dominant descriptor, such as pore volume for methane storage.

These results show that expert intuition can be quantified, put in use, and even evaluated when knowledge across an entire material class is systematically collected and structured, as demonstrated here with MOF-ChemUnity. More broadly, they demonstrate how expert recommendations, when embedded into a structured and navigable chemical space, can serve as powerful priors for materials discovery. This is particularly useful in low-data regimes such as early stage screening, where only a handful of high-quality examples exist. By integrating expert-labeled MOFs into the graph, MOF-ChemUnity enables researchers to explore chemically meaningful neighborhoods, expand sparse data sets, and accelerate the search for promising candidates grounded in both human judgment and structural similarity.

### Toward Literature-Informed AI Research Assistants

A central motivation of this work is to enable AI systems that operate over the full scope of MOF knowledge, not just structured computational data, and demonstrate robust performance across a range of scientific tasks. Recently, LLMs have gained widespread attention for their general-purpose capabilities, including retrieving, synthesizing, and summarizing scientific content, even for tasks on which they were not explicitly trained.^49,50^ However, their use in scientific research remains limited due to frequent hallucinations, confidently producing responses not grounded in any source. This lack of factual traceability undermines their trustworthiness in research contexts, where evidence is essential. Although RAG methods offer a promising solution by supplying LLMs with domain-specific documents to improve factual grounding, existing RAG systems typically rely on limited corpora (e.g., abstracts or article subsets) and struggle with noise and scale when applied to the full literature. MOF-ChemUnity offers an alternative: a distilled, structured representation of key entities and relationships from across the MOF literature. Here, we show that augmenting LLMs with the MOF-ChemUnity knowledge graph enables a literature-informed AI assistant capable of more accurate and interpretable scientific reasoning.

We propose a graph-enhanced RAG approach, where we use MOF-ChemUnity as a domain-specific, high-density context layer for question answering. The graph filters out irrelevant content while preserving factual and relational information, making it well-suited for guiding retrieval. Using an LLM-based query tool, we retrieve the relevant subgraphs and construct a prompt using structured information from the graph (Figure 6a). This rich context is then passed to an LLM, which generates responses grounded in evidence explicitly encoded in the knowledge graph. This design improves the interpretability, consistency, and factual accuracy of LLM outputs on domain-specific tasks.

**Figure 6:**
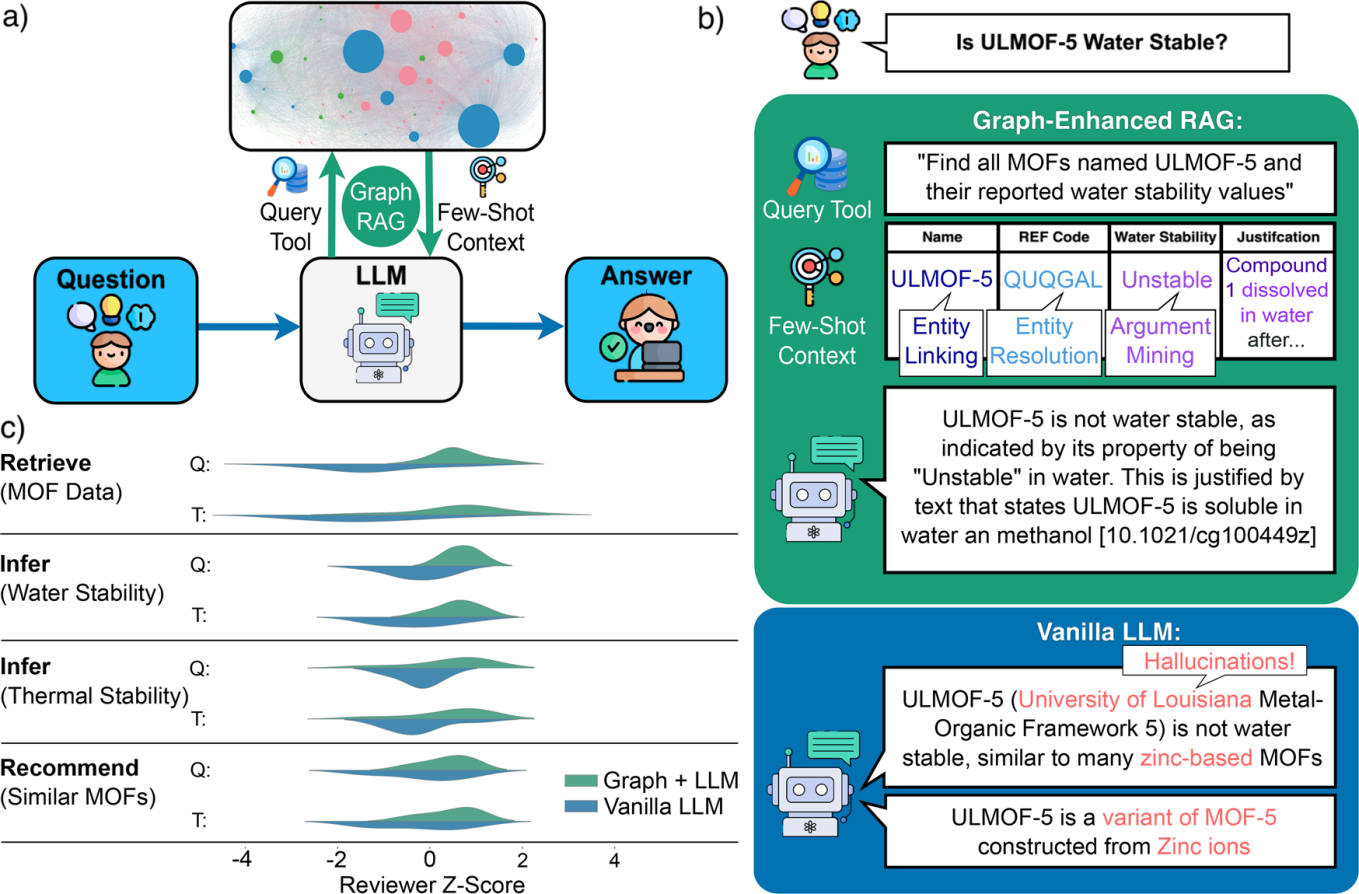
Knowledge Graph Enhanced RAG as a Literature-Informed AI Assistant. (a) The graph-enhanced RAG workflow uses a query tool to retrieve relevant information from the knowledge graph and use it as few-shot context. (b) Example responses show that the vanilla LLM (GPT-4o^51^) is prone to hallucinations, whereas the graph-enhanced workflow uses entity linking, entity resolution, and argument mining to correctly answer the question and provide a citation in the answer. (c) Violin plots detailing the results of the response comparison survey from nine expert chemists on tasks including retreival of MOF data from literature, inference of structure–property relationships related to MOF water stability and thermal stability, and recommending a MOF similar to a given MOF. The graph-enhanced workflow scored higher in both quality (Q) and perceived trustworthiness (T) for all four questions.

Figure 6b illustrates this approach in practice. Banerjee et al.^52^ synthesized a Li-based MOF, called ultralight MOF (ULMOF-5), and referred to it in their paper as “compound 1”. When querying a standard LLM (here, GPT-4o with and without websearch^51^) about the water stability of ULMOF-5, it returns hallucinated responses, confusing it with the unrelated Zn-based MOF-5 due to name similarity. In contrast, MOF-ChemUnity links all coreferences to the correct crystal structure and captures a water stability label (“unstable”) derived from the sentence “compound 1 is soluble in water” from the paper. Our system retrieves this information and provides a factually grounded answer with citation and explanation, improving both accuracy and transparency.

To further evaluate our system, we compared responses from the graph-enhanced RAG and vanilla LLM (here, GPT-4o) across three tasks: factual retrieval, structure–property inference, and material recommendation. Nine MOF experts assessed the quality and perceived trustworthiness of responses in a blinded survey (see SI subsection 12.2). Figure 6c shows that the graph-augmented assistant was rated higher across all tasks. In particular, experts valued its inclusion of citations, specific examples, and verifiable claims, whereas baseline responses were often generic, ungrounded, or unverifiable. These findings demonstrate that augmenting LLMs with structured scientific knowledge improves both factual reliability and user confidence.

An intriguing observation in the survey was that some experts noted that while the graph-based system cited relevant literature, it occasionally reflected outdated views, for example, early assumptions about water stability, which recent sorption studies have since challenged and revised. This highlights an important and fundamental point: science evolves, and AI systems must remain dynamic and reflective of current understanding. Enabling scientific assistants with such abilities will be a grand next challenge. Nonetheless, the transparency of the graph-enhanced approach, citing sources and tracing reasoning, empowers human users to judge the validity of each response, building trust through verifiability.

Our simple workflow illustrates how resolving MOF names, linking them to crystal structures, and capturing literature-derived properties in MOF-ChemUnity enables LLMs to operate as trustworthy, literature-informed assistants. By distilling MOF knowledge into a structured, queryable, and interpretable format, the knowledge graph serves as both a factual substrate and a reasoning scaffold. It supports not only textual evidence but also structured tools such as similarity search, paving the way for scalable, verifiable, and domain-specialized AI systems in scientific discovery.

## Outlook and Concluding Remarks

The main goal of this work was to develop a framework that distills knowledge from the vast MOF literature into a unified, structured, and machine-readable format, that is connected to crystal structures and computational data, enabling both scientists and AI systems to leverage the full breadth of MOF knowledge. MOF chemistry spans a wide range of applications, making it rich in information but challenging to navigate. For example, how can a researcher working on magnetic MOFs benefit from the latest findings in gas separation, when relevant insights may be buried in a different subfield? These insights often remain underutilized, even when similar materials or mechanisms are involved. We address this by disambiguating MOF names and crystal structures and organizing the extracted information into a knowledge graph, MOF-ChemUnity, linking experimental data, computational results, and expert recommendations in a format suitable for advanced search and analysis. This structure allows researchers to ask high-level scientific questions and enables AI models to reason over MOF chemical space in a grounded and interpretable way. This enables a new way of interacting with the literature, one that goes beyond reading papers individually or collecting data manually.

One of the practical motivations of this work was to enable cross-document linking of information about the same MOF. Given that different names or labels are often used for a single compound, traditional literature searches often fail to connect these scattered pieces of information. MOF-ChemUnity resolves this by identifying and linking all coreferences and names to a single material entity, allowing researchers to recover and analyze information across publications in one place. As a demonstration, Figure 7 shows how this enables the reconstruction of complete synthesis procedures by aggregating data from multiple sources, including articles not linked to MOF-ChemUnity and the CSD, or to the original synthesis report. For example, HKUST-1, Cu-BTC, and MOF-199 all refer to the same compound, from which data can be resolved and seamlessly combined in MOF-ChemUnity. By extracting synthesis procedures for this compound from over 200 papers, we uncover a variety of ways to synthesize the same material (see methods section).

**Figure 7:**
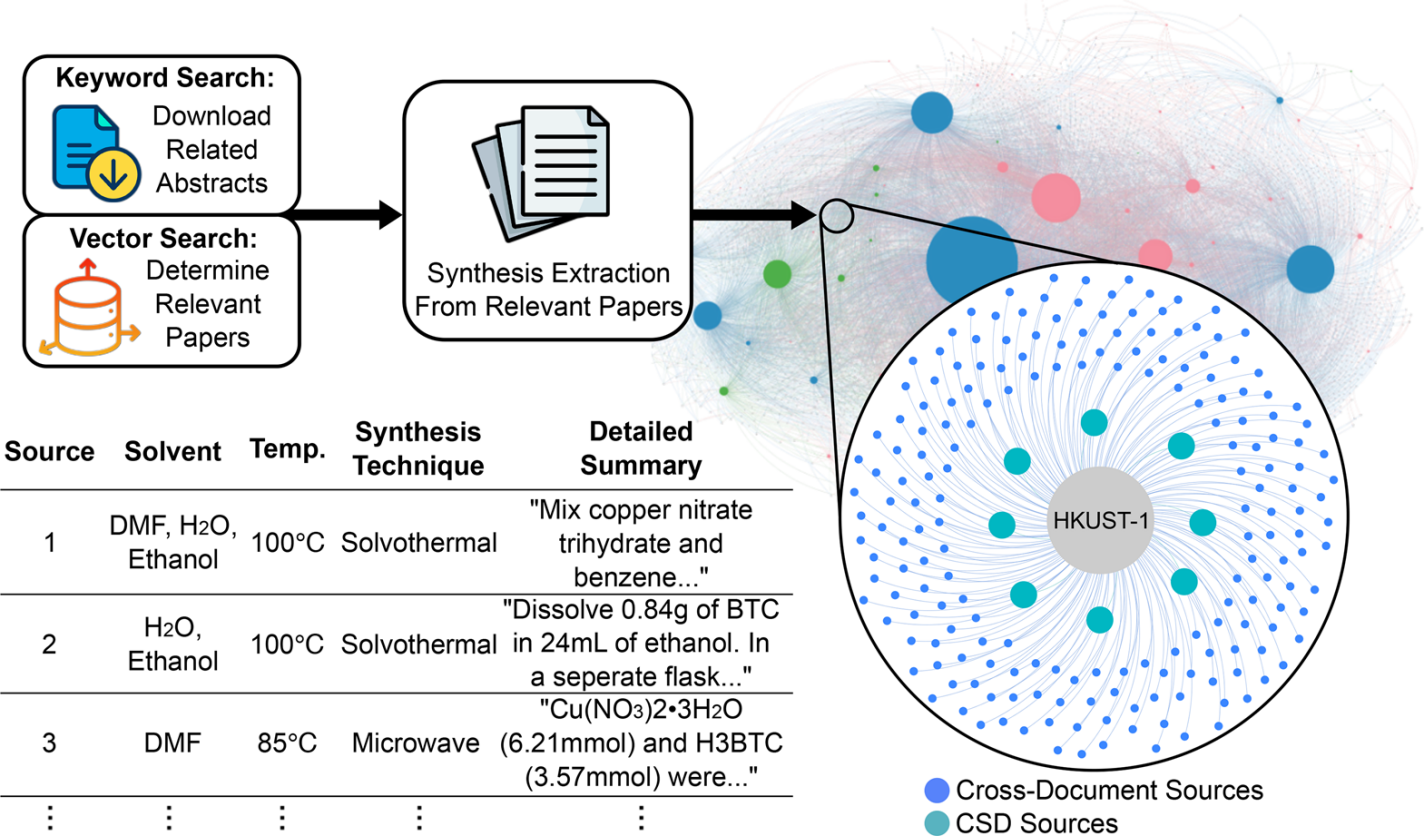
Cross-Document Extraction and Linking. By matching MOF names and CSD reference codes, MOF-ChemUnity enables synthesis information to be aggregated from sources beyond the CSD. This figure illustrates the text-mining of synthesis procedures for HKUST-1 across multiple articles, linking each to its corresponding crystal structure in the knowledge graph. Results can be viewed interactively as a graph or exported as a table.

The case studies presented in this work were intended as a proof of principle that data extraction and interlinking across computational, structural, and experimental sources is both feasible and useful. A critical consequence of such integration, however, is the inhomogeneity of data and the lack of uniformity, fidelity, or quality. For instance, on the structural data side, computation-ready databases such as CoRE MOF 2019 are known to contain errors,^53^ including the inclusion of non-MOF structures and the removal of interpenetrated frameworks; our approach inherits these constraints. Another potential challenge is that the literature may contain information that contradicts the previously extracted data in the graph. This issue does not arise in the present study, since each MOF is associated with a unique MOF–article pair, and no large scale cross-document extraction was performed. Nevertheless, future large-scale extractions will inevitably encounter conflicting reports. By storing data within a knowledge graph, MOF-ChemUnity provides a flexible solution to resolve such contradictions: for example, each synthesis or characterization procedure is stored as a distinct “Has Synthesis” relationship, annotated with metadata such as source, publication date, and justification (see methods and SI subsection 11.2 for more details). This allows both human experts and machine-learning systems to assess the reliability and context of individual data points, and to reconcile discrepancies that may emerge across documents. With the field moving toward standardized formats for reporting, such as the Adsorption Information File (AIF) proposed by IUPAC,^54^ a promising next step is the integration of MOF-ChemUnity with these standards, enabling a platform to unify heterogeneous data while maintaining traceability and interpretability.

We envision MOF-ChemUnity as a practical resource for both researchers and AI systems. From a database perspective, linking MOF names to crystal structures allows the database to be continuously expandable with new publications and appendable with future computational annotations. In addition, the cross-document workflow enables further continuous expansion of the database, supporting large-scale extraction of information across the literature. This design is scalable and appendable, making it suitable for long-term use as the field continues to grow. While this work focuses on MOFs, the overall framework, that is combining large language models, entity linking, and graph-based representation, is general and can be extended to other material classes, such as covalent organic frameworks, zeolites, polymers and beyond. Applying these techniques to other material classes requires domain knowledge to properly diagnose field-specific challenges. For fields facing similar challenges to MOFs, such as a lack of a standard naming convention and data heterogeneity, MOF-ChemUnity provides a strong blueprint for unifying information.

## Methods

### Data Set Curation

To have a starting list of MOFs, we start with the Computation Ready MOF database (CoRE MOF 2019) and Quantum MOF database (QMOF).^13,36^ These databases include 31,068 unique crystal structures. Only entries with computational labels for gas uptake or band gap within either data set are considered. From those entries, only the ones with CSD reference codes were used as input to the workflows since CSD contains the DOI that links this MOF entry to the original discovery paper. The final data set is the intersection of the CSD with the union of the CoRE MOF 2019 and QMOF structures. The full-texts were obtained using the text and data mining (TDM) method corresponding with each of the 5 publishers selected (see SI section 9). The full-texts obtained had two formats, XML (Elsevier and American Chemical Society) and PDF (Royal Society of Chemistry, Wiley, International Union of Crystallography). For XML documents, the Pub2Tei^55^ library was used to convert the custom XML files to a standardized XML format. The documents were then converted to a markdown file format using a Python script. Similarly, PDF were converted to markdown files using Marker^56^ library which uses various models to convert PDFs.

### LLM Matching and Extraction Agents

Once the publications are converted to markdown, large language model (LLM) workflows were used for matching and extraction. An LLM workflow here refers to a specialized chain that executes a sequence of instructions to achieve a single goal. Previous studies have evaluated various LLMs on extraction tasks in chemistry.^16,57,58^ Based on their findings, we selected a state-of-the-art LLM (GPT-4o through OpenAI API) for all workflows to ensure high accuracy and fast inference time. Critically, OpenAI provides a robust structured output end point which allows each workflow to output a structured CSV file.^59^

### Matching MOF Names with Crystal Structures

The first part of our workflow aims to solve the problem of named entity recognition, coreference resolution and unique entity linking for MOFs mentioned in a single publication. Our solution is to provide the LLM with crystal structure-derived information to match the MOF names within the publication to the corresponding CSD reference code. The information includes CSD reference code, lattice parameters, metal node, space group, molecular formula, chemical name, and any known synonym, all obtained using CSD’s Python API for each MOF.^60,61^ The LLM is instructed to find which unique MOF names in the paper correspond to each of the CSD reference codes given. This ensures that there is a unique one-to-one link between CSD reference codes and MOF names from a single paper. It is also instructed to find all the coreferences associated with the MOF. By isolating tasks such as MOF name matching and coreference resolution, we enable fine-grained accuracy evaluation at each step. This design ensures that all extracted MOF mentions are accurately linked to their corresponding CSD reference codes, providing a reliable foundation for subsequent information extraction.

### General Extraction Workflow

The MOF names extracted by the matching workflow are used for information extraction ensemble. In this ensemble, multiple workflows are instructed to use the MOF names and extract different information associated with these names such as properties, recommended applications and synthesis information. These workflows also receive a customized output structure to ensure consistency in the outputs. Finally, a manually curated dictionary maps the various names and abbreviations, which the LLM extracted for properties and applications, to a single unique term (see SI subsection 3.2). Any extracted property or application that does not get mapped using the dictionary is removed from the extraction output. Consequently, eliminating this noise ensures that the extracted information is useful and easy to navigate.

### Specialized Extraction Workflow

Extracting certain properties in one-shot can be confusing for LLM. For example, water stability can be confused with thermal, mechanical, and chemical stability. For these properties, we adapt a Chain of Verification (CoV) approach to improve the accuracy of extraction,^35^ in which the LLM verifies that the water stability justifications extracted are indeed discussing water stability. Therefore, reducing hallucinations and improving extraction accuracy and consistency (see SI subsection 3.3 for implementation details and examples).

### Building and Accessing the Knowledge Graph

Neo4j^62^ was selected as the platform to construct the MOF-ChemUnity knowledge graph, due to its relative ease of use and visualization capabilities. Neo4j uses a native query language called “Cypher”, which enables efficient creation, modification, and querying of graph data. Using our predefined schema, the resulting knowledge graph was populated with over 40,000 nodes and 3,200,000 relationships.

To make our knowledge graph accessible to users unfamiliar with graph query languages, we developed a natural language query tool powered by LLMs. This tool translates users’ natural language requests into executable Cypher queries, automatically retrieves relevant data from the knowledge graph, and presents the results in a tabular format. This enables users to explore and analyze complex MOF data sets without needing prior knowledge of the graph structure or Cypher syntax.

### MOF Duplicate Structure Detection

The schema in MOF-ChemUnity links each MOF structure, “MOF” node, to its names and coreferences. These structures are identified using their CSD reference code. Therefore, each “MOF” node is a unique CSD reference code. However, multiple MOF structures can be duplicates although they are assigned different reference codes. Since the starting set of MOFs in this work is obtained from both CoRE MOF 2019 and QMOF, MOF-ChemUnity inherits a degree of duplicate handling from the two data sets. Moreover, the schema also enables duplicate detection as multiple “MOF” nodes from different papers can be linked to the same name which indicates that these structures are duplicates. In other words, if different papers deposited MOF structures to CSD but the authors refer to their MOF using the same name, then this indicates that the authors simply synthesized the same structure.

### Graph-Enhanced Retrieval Augmented Generation

Our graph-enhanced RAG system retrieves relevant information and use it as few-shot context for general question-answering. The framework also incorporates machine learning-based embeddings to identify structurally or chemically similar MOFs, supporting more informed question answering. The core components—namely the Query and Neighbor Finder tools—are modular and can be invoked by an AI agent as needed.

### MOF Recommendations

To embed MOF crystal structures in a chemically meaningful vector space, we combined chemical and geometric descriptors. Specifically, we used revised autocorrelation (RAC) descriptors to capture the chemistry of each MOF,^11,63^ and geometric features, including pore volume and pore diameter, computed using Zeo++.^47,48^ These descriptors were calculated for approximately 15,000 MOFs from the CoRE MOF 2019 ^36^ and QMOF^13,38^ databases. We then projected the high-dimensional descriptor space into two dimensions using t-distributed stochastic neighbor embedding (t-SNE). Based on this projection, we implemented a nearest-neighbor approach (with k = 6) to recommend candidate materials: given a MOF with a known application label (for instance, extracted via LLMs), similar MOFs were identified based on their proximity in the embedding space and proposed for the same application. To assess the validity of this approach, we compared gas uptake values for recommended MOFs against two baselines: (1) random sampling from CoRE MOF 2019, and (2) the subset of MOFs explicitly recommended in the literature (based on LLM-extracted application annotations). The gas uptakes values were obtained from prior molecular simulations.^11^

## Supplementary Material







## Data Availability

All code used in this study—including LLM workflows for MOF name matching, property and synthesis extraction, and cross-document linking, as well as associated prompts—is openly available under the MIT license at https://github.com/AI4ChemS/MOF_ChemUnity. In addition, for reproducibility, we included benchmarking data sets for matching and extraction workflows, along with an example notebook demonstrating data extraction and graph integration for a set of four publications. Moreover, a spreadsheet of publication information for all papers considered in this work is available as a supplementary file. Due to publishers copyright restrictions on large-scale text and data mining, only a subset of the extracted data is freely available. This includes the full list of resolved CSD reference codes with associated MOF names and coreferences from publications (available as a supplementary file and on github https://github.com/AI4ChemS/MOF_ChemUnity) as well as the water stability labels used to train the machine learning models in this work (available on github https://github.com/AI4ChemS/MOF_ChemUnity). All extracted data from 100 representative publications is provided as a demonstration set.

## Notes

Details on selection, acquisition, and preprocessing of research documents; additional information on LLM workflows, prompts, and benchmarks; analysis of MOF metal types and topologies in the knowledge graph; additional details regarding the expert survey and Graph-enhanced RAG; in-depth details about water stability model training and optimization; detailed analysis of water stable MOFs for carbon capture. (PDF)
List of CSD reference codes, matched names and coreferences. (XLSX)
List of DOIs and titles of the papers considered in this work. (XLSX)
