# Supplementary material for: MOF-ChemUnity: Literature-Informed Large Language Models for Metal–Organic Framework Research
Source: J Am Chem Soc. 2025 Nov 10;147(47):43474–86. doi: 10.1021/jacs.5c11789 (PMC12673590; doi:10.1021/jacs.5c11789)
Supplement: Supplementary file 1 [file ja5c11789_si_001.pdf]

Supplementary Materials

MOF-ChemUnity: Literature-informed large language models for metal-organic framework research

Thomas Michael Pruyn,[†,§] Amro Aswad,[†,§] Sartaaj Takrim Khan,[†] Ju Huang,[†] Robert Black,[‡] and Seyed Mohamad Moosavi[*,†,¶]

†*Chemical Engineering & Applied Chemistry, University of Toronto, Toronto, Ontario M5S 3E5, Canada*

‡*Clean Energy Innovation Research Centre (CEI), National Research Council Canada, Mississauga, Ontario L5K 1B4, Canada*

¶*Vector Institute for Artificial Intelligence, Toronto, Ontario M5G 0C6, Canada*

§*Contributed equally to this work*

E-mail: mohamad.moosavi@utoronto.ca

Contents

1 Overview of MOF ChemUnity Workflows **4**

2 Crystal Structure and Name Matching Workflow **5**

3 Property Extraction **11**

3.1 General Property Extraction 11

3.2 Property Filter and Name Standardization 14
3.3 Specific Property Extraction 21

4 Application Extraction **34**

5 Synthesis Procedure Extraction **43**

6 Benchmarks **44**

6.1 Matching Benchmark Results 44
6.2 General Property Extraction Results 45
6.3 Synthesis Procedure Extraction Results 45

7 Workflow Information **45**

7.1 Retrieval-Augmented Generation Information 46
7.2 LLM Information 46

8 Selection of the Starting Crystal Structures **47**

8.1 CoRE MOF 2019 Entries 49
8.2 QMOF Entries 50
8.3 Input Dataset 50

9 Obtaining Publications Full-Texts **50**

9.1 Identification of Publishers 51
9.2 Full-text Formats 51
9.3 Converting PDF to Markdown 52
9.4 Converting XML to Markdown 52
9.5 Creating Vector Stores 53

10 Extracted Dataset **53**

10.1 Analysis of Matching Workflow Output 54
10.2 Analysis of Property Extraction Workflow 55

10.3 Analysis of Application Extraction Workflow 57

11 MOF ChemUnity Knowledge Graph 62

11.1 Building the Knowledge Graph 62

11.2 Data Provenance 62

11.3 Query Agent Tool 63

12 Graph-Enhanced RAG 66

12.1 Tasks 66

12.1.1 Property Prediction 66

12.1.2 Retrieval 66

12.1.3 Structure-Property Inference 67

12.1.4 Recommendation 67

12.2 Survey 68

13 Water stability modeling 70

13.1 ML Features: RACs and geometric descriptors 70

13.2 Machine learning to predict water stability of MOF 71

13.3 Baseline model 71

13.4 Hyperparameter optimization 72

13.5 Impact of probability threshold 75

13.6 Uncertainty assessment 77

13.7 Feature importance in water stability model 79

13.8 SHAP analysis on model 80

14 Water stable MOFs for carbon capture 83

14.1 MOF curation workflow 83

14.2 DFT and molecular simulation details 85

References 87

1 Overview of MOF ChemUnity Workflows

As shown in Figure S.1, the workflow find a list of crystal information files (CIFs) that are in CoRE MOF 2019[1] or QMOF[2,3] with computed gas uptake or band gap labels and have a Cambridge Structural Database[4] (CSD) reference code. For each CIF in that list, unique crystal identifiers, CSD reference codes and DOI were obtained and stored. The crystal identifiers include crystal information from CSD that is unique to each CIF. The CSD reference code is also added because it is going to be used to form a unique one-to-one link between extracted properties and computational databases. The DOI is used to identify the list of publications considered for this work as well as obtain the full-texts later. In this work, all information from CoRE MOF 2019, QMOF, computed geometric descriptors and revised auto-correlations (RACs) are considered computational data. Any information obtained from the publication during information extraction is considered experimental data.

The publications corresponding to these CIF files were then obtained in accordance with the method mentioned by the publisher's TDM license for each publication. For Elsevier and Wiley, they provide a TDM API to obtain full texts. For Royal Society of Chemistry (RSC) and International Union of Crystallography (IUCr), they allow using scripts to download full texts with a delay between requests. American Chemical Society does not have a TDM API and full-texts were obtained via a special request. These publications were obtained in PDF or XML format, depending on availability. These formats were processed further converted to Markdown (MD) which is more LLM friendly. However, the conversion isolates figures and charts from the text in the publication. In this work, only the text was used for the LLM workflows as it contains most of the information of interest.

The matching workflow takes all CSD reference codes and the corresponding crystal identifiers available for each publication. That is, a single publication can have multiple MOFs and multiple CIF files deposited into CSD. This workflow uses retrieval-augmented generation (RAG).[5] One difference is that large chunks of the publication text are given to the LLM to provide as much context as possible. The output of this workflow is a table

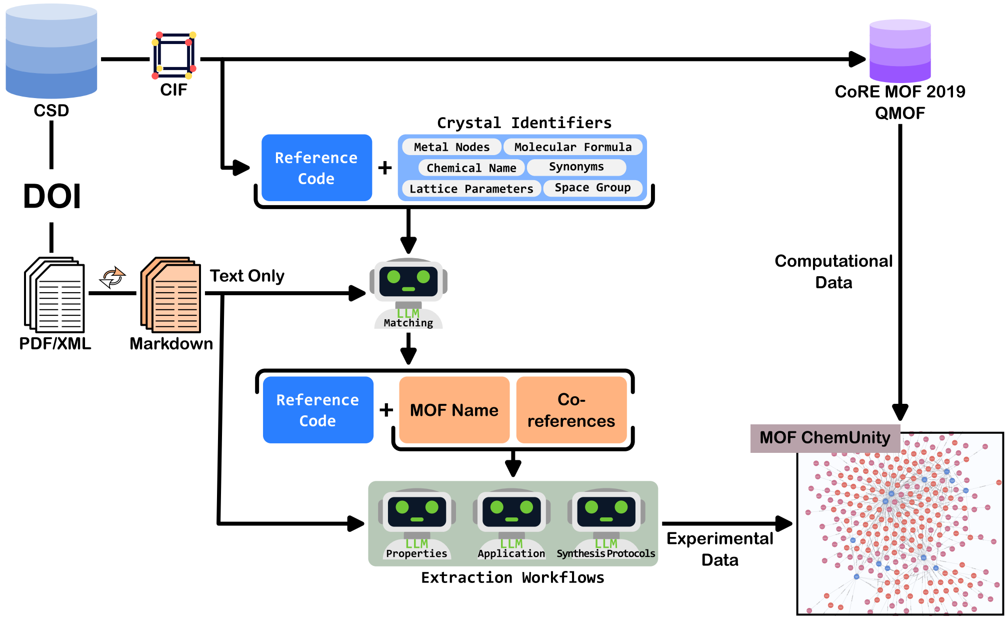

Figure S. 1. Overall workflow to create MOF ChemUnity. The workflow starts with crystal information files (CIFs) and combines computational and experimental information via a unique identifier, which is the Cambridge Structural Database (CSD) Reference Code, and uses a series of LLM calls to extract the data required to create MOF-ChemUnity knowledge graph.

containing the CSD reference code, the corresponding MOF name and any co-references to that name.

The MOF names and co-references that have been matched with a CSD reference code are then used in the extraction workflows. The extraction workflows extract the properties, applications, and synthesis information for each CSD reference code individually. In this way, the extracted output is said to be strictly related to CIF file with the matching CSD reference code.

2 Crystal Structure and Name Matching Workflow

The main purpose of this work is to link experimental data from publications to available computational datasets. To achieve this, CSD reference codes from both CoRE MOF 2019 and QMOF were collected and MOF crystal information was appended from CSD. These include 1) CSD reference code, 2) Space group, 3) Metal types, 4) Lattice parameters (a,

b, c), 5) Chemical name, 6) Molecular formula and 7) Synonyms. The crystal information thereof is provided to the LLM in the prompt as identifiers to help it match the MOF name and co-references from the publication to the corresponding CSD reference code. The space group, lattice parameters, chemical name and synonyms were collected from CSD using the csd-python-api. Molecular formula and metal types were collected using Pymatgen[6] and CoRE MOF 2019/QMOF CIF files. There are significant discrepancies between formulas reported in papers, those in CSD CIFs, and those computed from CoRE/QMOF CIFs. These differences arise because various types of formulas may be reported (e.g., empirical vs. structural), and authors are inconsistent in whether they include guests or solvents. To assess the sensitivity of our workflow to such mismatches, the matching agent was also benchmarked using chemical formulas obtained directly from the CSD API. The results showed negligible differences (95% vs. 96% accuracy), confirming that formula inconsistencies do not significantly affect performance. It should be noted that the LLM often relies on different identifiers as "hints" for different cases of matching MOF Names to reference codes, and as this analysis indicates, the LLM is capable of making correct decisions even when all clues do not directly match what is found in the paper. To indicate which hints were used in the matching process, the LLM also outputs a justification for its answer. Figure S.2 shows the prompt and figures S.3, S.4 and S.5 show example input and output of this step.

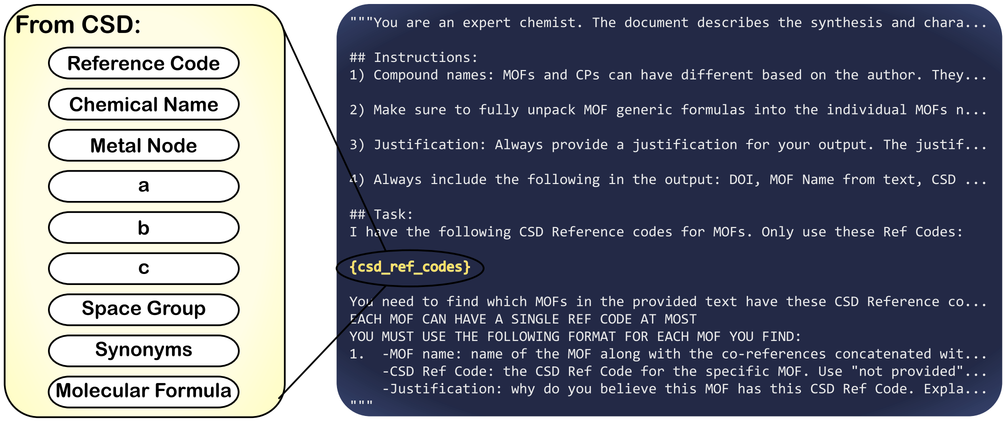

Figure S. 2. Matching prompt template The prompt takes CSD information as input to guide the LLM when matching CSD reference codes to the named MOF entities in the publication.

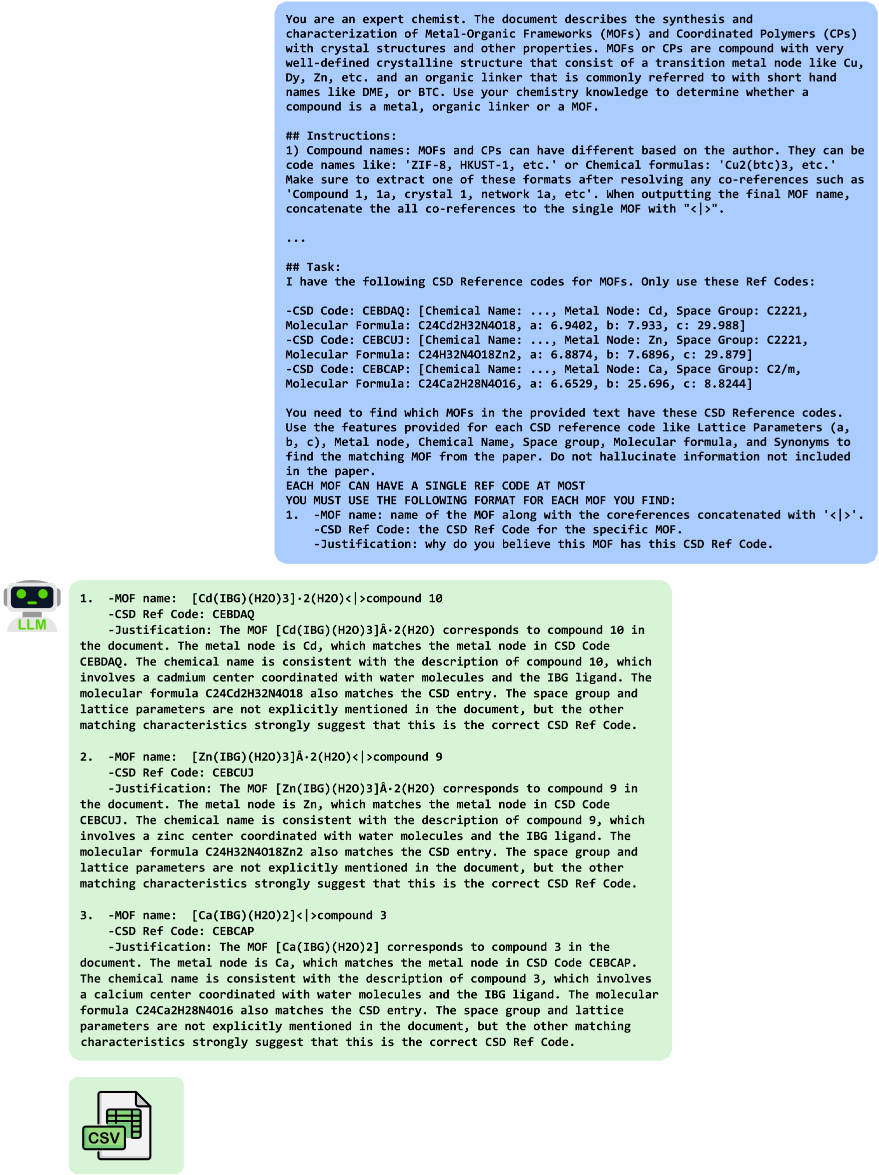

Figure S. 3. Matching example for single DOI. In this example, the LLM should match only a subset of all MOFs mentioned by the authors.

```
You are an expert chemist. The document describes the synthesis and
characterization of Metal-Organic Frameworks (MOFs) and Coordinated Polymers (CPs)
with crystal structures and other properties. MOFs or CPs are compound with very
well-defined crystalline structure that consist of a transition metal node like Cu,
Dy, Zn, etc. and an organic linker that is commonly referred to with short hand
names like DME, or BTC. Use your chemistry knowledge to determine whether a
compound is a metal, organic linker or a MOF.

## Instructions:
1) Compound names: MOFs and CPs can have different based on the author. They can be
code names like: 'ZIF-8, HKUST-1, etc.' or Chemical formulas: 'Cu2(btc)3, etc.'
Make sure to extract one of these formats after resolving any co-references such as
'Compound 1, 1a, crystal 1, network 1a, etc'. When outputting the final MOF name,
concatenate the all co-references to the single MOF with "<|>".

...

## Task:
I have the following CSD Reference codes for MOFs. Only use these Ref Codes:

-CSD Code: UGUKUD: [Chemical Name: ..., Metal Node: Zn, Space Group: P21/c,
Molecular Formula: C28F4H16O20P4Zn8, a: 5.8011, b: 19.139, c: 8.5616]

You need to find which MOFs in the provided text have these CSD Reference codes.
Use the features provided for each CSD reference code like Lattice Parameters (a,
b, c), Metal node, Chemical Name, Space group, Molecular formula, and Synonyms to
find the matching MOF from the paper. Do not hallucinate information not included
in the paper.
EACH MOF CAN HAVE A SINGLE REF CODE AT MOST
YOU MUST USE THE FOLLOWING FORMAT FOR EACH MOF YOU FIND:
1.  -MOF name: name of the MOF along with the coreferences concatenated with '<|>'.
    -CSD Ref Code: the CSD Ref Code for the specific MOF.
    -Justification: why do you believe this MOF has this CSD Ref Code.
```

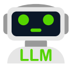


```
1.  -MOF name:  Zn2(OOCC6H4PO3)F<|>compound 2
    -CSD Ref Code: UGUKUD
    -Justification: The compound Zn2(OOCC6H4PO3)F, referred to as compound 2 in the
document, matches the CSD Ref Code UGUKUD based on several key features. The space
group for compound 2 is P21/c, which matches the space group provided for UGUKUD.
The metal node is zinc (Zn), which is consistent with the metal node in UGUKUD.
Additionally, the lattice parameters a, b, and c for compound 2 are 5.8011, 19.139,
and 8.5616, respectively, which match the lattice parameters provided for UGUKUD.
The chemical name "catena-((Î¼7-4-Carboxylatophenylphosphonato)-(Î¼3-fluoro)-di-
zinc)" also aligns with the description of compound 2, where the fluoride anion
serves as a bridging ligand coordinating to zinc atoms. These consistent features
justify the assignment of the CSD Ref Code UGUKUD to Zn2(OOCC6H4PO3)F.
```

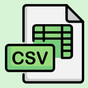

Figure S. 4. Another example of matching. This one is given a single CSD reference code while the paper mentions multiple compounds.

```
You are an expert chemist. The document describes the synthesis and
characterization of Metal-Organic Frameworks (MOFs) and Coordinated Polymers (CPs)
with crystal structures and other properties. MOFs or CPs are compound with very
well-defined crystalline structure that consist of a transition metal node like Cu,
Dy, Zn, etc. and an organic linker that is commonly referred to with short hand
names like DME, or BTC. Use your chemistry knowledge to determine whether a
compound is a metal, organic linker or a MOF.

## Instructions:
1) Compound names: MOFs and CPs can have different based on the author. They can be
code names like: 'ZIF-8, HKUST-1, etc.' or Chemical formulas: 'Cu2(btc)3, etc.'
Make sure to extract one of these formats after resolving any co-references such as
'Compound 1, 1a, crystal 1, network 1a, etc'. When outputting the final MOF name,
concatenate the all co-references to the single MOF with "<|>".

...

## Task:
I have the following CSD Reference codes for MOFs. Only use these Ref Codes:

-CSD Code: FURSES: [Chemical Name: ..., Metal Node: Cu, Space Group: P21/m,
Molecular Formula: C18Cu4H8N8O10, a: 10.015, b: 6.6124, c: 12.4721]
-CSD Code: FUSGOR: [Chemical Name: ..., Metal Node: Co, Space Group: P21/m,
Molecular Formula: C18Co4H8N8O10, a: 10.1792, b: 6.7279, c: 12.8158]

You need to find which MOFs in the provided text have these CSD Reference codes.
Use the features provided for each CSD reference code like Lattice Parameters (a,
b, c), Metal node, Chemical Name, Space group, Molecular formula, and Synonyms to
find the matching MOF from the paper. Do not hallucinate information not included
in the paper.
EACH MOF CAN HAVE A SINGLE REF CODE AT MOST
YOU MUST USE THE FOLLOWING FORMAT FOR EACH MOF YOU FIND:
1.  -MOF name: name of the MOF along with the coreferences concatenated with '<|>'.
    -CSD Ref Code: the CSD Ref Code for the specific MOF.
    -Justification: why do you believe this MOF has this CSD Ref Code.
```

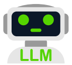


```
1.  -MOF name: [Cu2(OH)(TZI)(H2O)2]n<|>compound 1
    -CSD Ref Code: FURSES
    -Justification: The MOF [Cu2(OH)(TZI)(H2O)2]n, referred to as compound 1 in the
document, matches the CSD Ref Code FURSES. The justification for this match is
based on several key features: the metal node is Cu, the space group is P21/m, and
the lattice parameters a, b, and c are 10.015, 6.6124, and 12.4721, respectively.
These parameters align with the details provided for FURSES. Additionally, the
chemical name "catena-((Î¼-5-(tetrazol-5-yl)isophthalato)-(Î¼-hydroxo)-diaqua-di-
copper(ii) trihydrate)" corresponds to the description of compound 1 in the
document.

1.  -MOF name: [Co2(OH)(TZI)(H2O)2]n<|>compound 2
    -CSD Ref Code: FUSGOR
    -Justification: The MOF [Co2(OH)(TZI)(H2O)2]n, referred to as compound 2 in the
document, matches the CSD Ref Code FUSGOR. The justification for this match is
based on the following features: the metal node is Co, the space group is P21/m,
and the lattice parameters a, b, and c are 10.1792, 6.7279, and 12.8158,
respectively. These parameters align with the details provided for FUSGOR.
Furthermore, the chemical name "catena-[(Î¼-5-(3,5-dicarboxylatophenyl)-
tetrazolato)-(Î¼-hydroxo)-diaqua-di-cobalt tetrahydrate]" corresponds to the
description of compound 2 in the document.
```

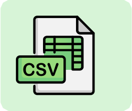

Figure S. 5. Last matching example. In this exmaple, the LLM is given the CSD reference codes for all the MOFs in the paper.

3 Property Extraction

Property extraction was categorized into two different workflows. The first workflow is general property extraction in which the LLM is instructed to extract all properties affiliated with the MOF names and co-references provided. The second workflow is specific property extraction, which allows user-defined prompts to provide the LLM with domain-specific knowledge about the property required for extraction. It also implements a variation of the Chain of Verification (CoV) from Ansari et al.[7]

3.1 General Property Extraction

As shown in Figures S.6 and S.7, the LLM is prompted once using a zero-shot prompt to extract all relevant properties. In this prompt, the LLM is provided with the list of names and coreferences for the MOF of interest. This LLM call also uses structured output API when available to ensure accurate parsing of the output to a CSV file. The structure output requires the fields shown in Table S.1.

Table S. 1. Structured Output for Property Extraction The fields of the structured output for property extraction. These are provided as a Pydantic object to the structured output API.

Field Name	Description
Property	The name of the property extracted
Value	The value of the extracted property (can be numerical or textual)
Units	The units of numerical values
Conditions	The conditions at which the value of the property is observed
Summary	The quote from the publication that contains the extracted information

```
You are an expert in coordinated chemistry and Metal Organic Frameworks (MOF).
You need to extract relevant physical, chemical and structural properties for a MOF
that has the following names: name 1: {[Cd(bdc)(4-bpmh)]}nÂ·2n(H2O) --- name 2:
compound 1.

Please be precise and try to be as accurate as possible. Make sure all properties
are related ONLY to the MOF Names provided.

Your output should have the following format for each property and its value:
1.  -Property Name: the name of the extracted property. Be consistent and use field
     relevant names. Do not include units here.
    -Property Value: the value for the property, please be precise and do not
     include units here, you need to fix any formatting issues.
    -Value Units: the units for this property. Include percentages, letters and
     signs that refer to units. You need to fix any formatting issues.
    -Conditions: the experimental conditions that this value was observed at.
    -Summary: The exact sentences from the provided text that mention the MOF Name
     and this property.
```

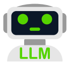


```
finding all properties of name 1: [[Cd(bdc)(4-bpmh)]]n·2n(H2O) ---name 2: compound 1

Result:
1.
   - Property Name: CO2 Uptake at 273 K
   - Property Value: 27.9
   - Value Units: cm3g-1
   - Conditions: 273 K
   - Summary: "The adsorption isotherms of CO2 for compounds 1 and 2 show a gradual increase and
     reach maximal amounts of 27.9 cm3g1 (5.4 wt%) and 43.2 cm3g1 (8.4 wt%) at 273 K,
     respectively."

...

8.
   - Property Name: Space Group
   - Property Value: P21/c
   - Value Units: N/A
   - Conditions: As determined by single-crystal structural analysis
   - Summary: "Compound 1 crystallizes in the monoclinic system with the space group P21/c,
     whereas compound 2 crystallizes in the triclinic system with the P1 space group."

9.
   - Property Name: Pore-Accessible Void Volume
   - Property Value: 1309.6
   - Value Units: Å3
   - Conditions: Per unit cell volume
   - Summary: "PLATON analysis revealed that dehydrated framework of 1 exhibits a large pore
     accessible void volume of 1309.6 Å3 out of 4705.9 Å3 that represents 27.8% per unit cell
     volume while 2 exhibits a void volume of 296.9 Å3 out of 1174.1 Å3 that represents 25.3 %
     per unit cell volume."
```

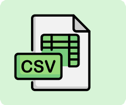

Figure S. 6. General extraction example. Both the MOF name and its in-text co-reference are provided to the LLM to gather all properties mentioned in the paper.

```
You are an expert in coordinated chemistry and Metal Organic Frameworks (MOF).
You need to extract relevant physical, chemical and structural properties for a MOF
that has the following names: name 1: [Cd(bpt)(fum)(H2O)]n ---name 2: complex 10.

Please be precise and try to be as accurate as possible. Make sure all properties
are related ONLY to the MOF Names provided.

Your output should have the following format for each property and its value:
1.  -Property Name: the name of the extracted property. Be consistent and use field
     relevant names. Do not include units here.
    -Property Value: the value for the property, please be precise and do not
     include units here, you need to fix any formatting issues.
    -Value Units: the units for this property. Include percentages, letters and
     signs that refer to units. You need to fix any formatting issues.
    -Conditions: the experimental conditions that this value was observed at.
    -Summary: The exact sentences from the provided text that mention the MOF Name
     and this property.
```

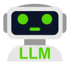


```
finding all properties of name 1: [Cd(bpt)(fum)(H2O)]n ---name 2: complex 10

Result:
1.  -Property Name: Empirical Formula
    -Property Value: C16H14CdN4O6S
    -Value Units: none
    -Conditions: none specified
    -Summary: "Synthesis of [Cd(bpt)(fum)(H2O)]n$nH2O (10). The synthetic procedure for 10 is
     similar to that for 5 except that Cu(NO3)2$8H2O was replaced by Cd(OAc)2$2H2O (0.2 mmol,
     0.053 g), affording yellow block crystals. Yield: 63% (0.063 g, based on bpt). Anal. calcd
     (%) for C16H14CdN4O6S: C 38.22, H 2.81, N 11.14. Found: C 38.14, H 2.95, N 11.19."

...

6.  -Property Name: Coordination Environment
    -Property Value: One nitrogen atom from bpt ligand and six oxygen atoms from three fum
     dianions
    -Value Units: none
    -Conditions: none specified
    -Summary: "The Cd(II) ion is in distorted pentagonal bipyramidal geometry coordinated by one
     nitrogen atom from bpt ligand (Cd–N 2.299(7) A) and six - oxygen atoms from three fum
     dianions (Cd–O 2.319(5) and 2.545(5) A)."

7.  -Property Name: Structural Feature
    -Property Value: Flat 2D (4,4) square grids
    -Value Units: none
    -Conditions: none specified
    -Summary: "The binuclear Cd(II) units are bridged by tetradentate and hexadentate fum
     dianions to form flat 2D (4,4) square grids with the dimensions of 10.87 7.64 A2 (Fig.
     10b)."
```

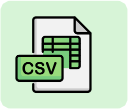

Figure S. 7. Another general extraction example. In this example, the authors use different co-reference naming scheme.

3.2 Property Filter and Name Standardization

In the general property extraction prompt, the LLM is given limited instructions, allowing it to extract many entries related to the specific MOF. Since many of these properties are not of interest, the results were truncated using a filter post-LLM call. The filter is a dictionary mapping a property name (key) to a list of property names that the LLM extracted (value) that refer to this property. The keys include a user-defined list of names for experimental properties of interest, such as CO_2 adsorption, thermal stability, etc. The mapped list of property names contains a subset of the property names extracted by the LLM which were manually added.

Matching property names to the corresponding filter dictionary keys is done using a fuzzy match score. This score is calculated using the *ratio* method within *thefuzz*[8] python library. Fuzzy match scores range from 0 (no similarity) to 1 (exact match). In this work, a threshold of 0.80 was used to determine whether two names are similar. For an extracted property name E_i, the algorithm iterates over all keys, K, and all property names in the corresponding list, P_K. The fuzzy match score, S_K, is computed between each $P_{K,i}$ and E_n. If S_K is higher than the threshold and the current maximum score, S_{max}, then S_{max} and matched key, K_{max}, are updated with S_K and K. Once both loops finished executing, the key K_{max} is returned as the matched property name.

To prevent duplication of property names after filtering, the key K_{max} is removed from the filter dictionary when looping over all extracted properties, E, for a single MOF. Another way to prevent false matches is to add the dictionary key *"Remove"*. This key is mapped to a list of extracted property names that can leak through the matching loops into the wrong keys. These properties are removed, thus, resulting in a very accurate property filter and name standardization. Lastly, if there are no matching properties in the filter, then the algorithm returns *"None"*. This indicates that these properties are not of interest, and consequently, they are removed. The pseudo code for the algorithm is in algorithm S.1. The filter dictionary is in table S.2 and properties considered for the *"Remove"* are in table S.3.

Algorithm S. 1. Property Name Filtering Algorithm

$E \leftarrow$ List of Extracted Properties for A Single MOF
$D \leftarrow$ Filter Dictionary $K \leftarrow$ keys: $P_{K_i} \leftarrow$ List of Names Mapped to Key K_i
$U \leftarrow$ Set of used keys from D
$t \leftarrow 0.80$ (Threshold set to 0.80)
for all E_n in E **do**
    $S_{max} \leftarrow 0$, $K_{max} \leftarrow$ *"none"*
    for all K_i in K and K_i not in U **do**
        $P_{K_i} \leftarrow D[K_i]$
        for all p_j in P_{K_i} **do**
            $S \leftarrow FuzzyRatio(E_n, p_j)$
            if $S > S_{max}$ and $S >= t$ **then**
                $S_{max} = S$
                $K_{max} = K_i$
            end if
        end for
    end for
    $E_n = K_{max}$
end for

Table S. 2. Properties Filter Dictionary. Any extracted property with a name similar to a Synonym will be have its name changed to the property key.

**Property Key**	**Mapped Synonyms**
Synthesis Yield	Synthesis Yield, Yield
Space Group	Space Group, Crystallographic Space Group
Crystal System	Crystal System
Cell Volume	Cell Volume, Unit Cell Volume, Crystal System Volume
Density	Density, Calculated Density, Density Calculated, dc, dcalc, d calc, d calcd, dcalcd, ρcalc, ρc, ρ, ρ calcd
Thermal Stability	Thermal Stability, Stability Temperature, Decomposition Temperature
Chemical Formula	Chemical Formula, Formula, Molecular Formula
Formula Weight	Formula Weight, Molecular Weight, Formula Mass, Molar Mass

**Property Key**	**Mapped Synonyms**
Light Absorption Coefficient	Light Absorption Coefficient, Absorption Coefficient
Topology	Topology, Framework Topology, Network Topology, Structural Topology
Langmuir Surface Area	Langmuir Surface Area, Surface Area Langmuir
BET Surface Area	Surface Area, Surface Area BET, BET Surface Area
Surface Area	Surface Area
Magnetic Susceptibility	Magnetic Susceptibility, Magnetic Susceptibility at Room Temperature, Magnetic Susceptibility at 300 K, Weiss Constant, Curie Constant
Magnetic Moment	Magnetic Moment, Effective Magnetic Moment
Pore Volume	Pore Volume, Void Volume, Void Space, Free Volume, Effective Free Volume, Porosity, Solvent-Accessible Volume, Solvent Accessible Volume
Pore Diameter	Pore Diameter, Pore Size

Property Key	Mapped Synonyms
Hydrocarbon Adsorption	C_3H_8 Uptake Capacity, C_2H_2 Storage Capacity, MeOH Adsorption, C_3H_8 Adsorption Capacity, C_2H_4 Uptake Capacity, Ethene Uptake, 1-Propanol Adsorption Capacity, Propyne Adsorption Capacity, Methanol Uptake, Ethene Uptake, C_3H_8 Adsorption Capacity, C_2H_4 Uptake Capacity, CH_3CH_2OH Sorption Capacity, C_2H_6 Uptake, C_2H_6 Storage, C_2H_6 Adsorption, Ethane Storage, Ethane Adsorption, Ethane Storage Capacity, Ethane Adsorption Capacity, C_2H_6 Adsorption Capacity, C_2H_4 Uptake, C_2H_4 Storage, C_2H_4 Adsorption, Ethene Storage, Ethene Adsorption, Ethene Storage Capacity, Ethene Adsorption Capacity, C_2H_4 Adsorption Capacity, C_2H_2 Uptake, C_2H_2 Storage, C_2H_2 Adsorption, Acetylene Adsorption, Acetylene Storage Capacity, Ethyne Adsorption, Ethyne Storage Capacity, Acetylene Adsorption Capacity, C_2H_2 Adsorption Capacity, Hexene Adsorption, Hexene Storage Capacity, Hexene Uptake, Hexane Adsorption, Hexane Storage Capacity, Hexane Uptake, Cyclohexane Storage, Cyclohexane Adsorption, Hexane Adsorption Capacity, Hexene Adsorption Capacity, CH_3OH Uptake, Benzene Adsorption Capacity
CO_2 Uptake	CO_2 Adsorption, CO_2 Uptake, CO_2 Adsorption Capacity, Carbon Dioxide Uptake, CO_2 Storage Capacity, Carbon Dioxide Storage, Carbon Dioxide Adsorption, CO_2 Adsorption Capacity, Carbon Dioxide Adsorption Capacity

Property Key	Mapped Synonyms
H_2 Uptake	H_2 Uptake Capacity, H_2 Adsorption Capacity, H_2 Sorption Capacity, H_2 Storage Capacity, H_2 Uptake, H_2 Storage, H_2 Adsorption, Hydrogen Uptake, Hydrogen Storage Capacity, Hydrogen Adsorption, Hydrogen Adsorption, Hydrogen Adsorption Capacity, H_2 Adsorption Capacity
N_2 Uptake	N_2 Uptake, N_2 Storage, N_2 Adsorption, Nitrogen Uptake, Nitrogen Storage Capacity, Nitrogen Adsorption, Nitrogen Adsorption, Nitrogen Adsorption Capacity, N_2 Adsorption Capacity
Color	Color, Crystal Color
Band Gap	Band Gap
Excitation Wavelength	Excitation Wavelength, Photoluminescent Excitation Wavelength, Emission Excitation Wavelength, Luminescence Excitation Maximum
Emission Wavelength	Emission Wavelength, Fluorescence Emission Wavelength, Fluorescence Emission Peak, Fluorescence Emission Maximum, Fluorescence Maximum, Fluorescence Peak, Photoluminescence Emission Wavelength, Photoluminescence Emission Maximum, Emission Maximum, Photoluminescence Emission Peak, Photoluminescence Maximum, Photoluminescence Peak, Luminescence Emission Wavelength, Luminescence Emission Maximum, Emission Peak, Luminescence Emission Peak, Luminescence Maximum, Luminescence Peak

Property Key	Mapped Synonyms
Coordination Geometry	Coordination Geometry, Coordination Environment, Coordination Geometry Of, Coordination Geometry -
Coordination Number	Coordination Number
Methane Uptake	Methane Storage, CH_4 Uptake Capacity, CH_4 Adsorption, CH_4 Uptake, CH_4 Adsorption, CH_4 Storage, CH_4 Storage Capacity, CH_4 Adsorption Capacity, Methane Adsorption Capacity, Methane Uptake, Methane Storage Capacity
Iodine Adsorption	Iodine Adsorption, Iodine Adsorption Capacity, I_2 Sorption Capacity, I_2 Adsorption

Table S. 3. Properties Removed. Any extracted property with a name similar to the synonyms in this table was removed.

Properties Removed		
Iodine Desorption Rate	Iodine Adsorption Rate	IR Absorption
CO_2 Adsorption Step	Dye Adsorption Capacity	N_2 and H_2 Adsorption
H_2S Uptake	Enthalpy of Carbon Dioxide Adsorption	N_2 Uptake Capacity
Bond Angles	Water Adsorption Capacity	D_2 Uptake
H_2O Uptake	Angles	Heat of Adsorption
Adsorption Energy	Uptake	Uptake Capacity
Unit Cell Angles	CD_4 Uptake	Storage Capacity
H_2 Adsorption Curve	Bond Distances	Adsorption Heat
Saturation Capacity	Hydrogen Adsorption Enthalpy	Hydrogen Bond Geometry

Carbon Dioxide Adsorption Enthalpy	O_2 Adsorption Capacity	Unique Reflections
Adsorption Enthalpy	Adsorption Sites	Hydrogen Storage Density
Unit Cell Parameter	Unit Cell Dimension	Cell Parameters
Gas Adsorption	Lattice Parameters	CO_2 Working Capacity
CO_2 Adsorption Order	IR Spectrum	IR Spectroscopy
IR Data	IR Spectra Peaks	IR Peaks
IR Absorption Bands	FTIR Peaks	Elemental Analysis (Carbon)
(Hydrogen)	(Nitrogen)	Hydrogen Bonds
O_2 Uptake	SO_2 Adsorption Capacity	Methanol Uptake Capacity
Carbon Content	Hydrogen Content	Metal Ion Adsorption Capacity
Wavelength	Emission Wavelength Range	Sorption Capacity
Photoluminescence	Photoluminescence Emission Maxima	Excess CH_4 Uptake Capacity
Luminescence	No Adsorption Capacity	Hg^{2+} Adsorption Capacity
Emission Peaks	N_2O Adsorption Capacity	N−BuOH Adsorption
Coordination Mode	Methanol Adsorption Capacity	Methanol Uptake
Coordination Sphere	SO_2 Uptake	Heat of Hydrogen Adsorption
NH_3 Uptake Capacity	Elemental Analysis	Chemical Formula Weight
H_2O Adsorption	MeOH Uptake	Oxygen Adsorption
Elemental Analysis - Carbon	I_2 Desorption Temperature	TLIEST Temperature
Elemental Analysis - Hydrogen	Reaction Temperature	Thermal Stability Ranking

Elemental Analysis - Nitrogen	Water Stability	Chemical Stability
CO_2 Uptake Capacity	CH_3OH Adsorption	Coordination Geometry Angles
Molecular Weight Control		

3.3 Specific Property Extraction

This workflow builds on the general property extraction by adding reasoning and extraction validation steps. This workflow adopts the Chain of Verification (CoV) Ansari et al. 2024,[7] with minor changes to the code base to make the CoV more robust and consistent.

The Chain of Verification (CoV) consists of 3 functions: 1) Initial extraction, 2) Verification, 3) Corrective Extraction. In the original work, CoV was implemented using a ReAct agent that calls the appropriate function at each step of the extraction.[7] Its performance was demonstrated for the extraction of water stability of MOFs from the presented literature. The changes made in this work simplify the implementation of CoV by using structured outputs and if-statements to determine function calls instead of a ReAct agent. Figure S.8 shows the simplified implementation. To accommodate this change, the prompts were also slightly altered, removing parts of the prompt that pertain to the ReAct agent. Figure S.9 shows the benchmark results for the simplified workflow with the altered prompt where it achieved 84.6%.

This workflow was also examined against the WS24 dataset from Terrones et al. 2024 which is a semi-automatically extracted water stability dataset for MOFs consisting of 1092 labels.[9] Our matched CSD reference codes include 363 entries from the WS24 dataset, of which the LLM labels only 142 entries. The reason that only 363 entries are common in total is because they collected papers from CoRE MOF 2019 and included MOFs without clean CIF files.[9] In this work, only CoRE MOF 2019 entries with clean CIF files were considered.

Furthermore, WS24 consists of WS14s (181 entries) and WS24s (911 entries).[9] Their WS14s does not necessarily originate from CoRE MOF 2019 as opposed to WS24s. Additionally, the 363 common entries include small number of entries that originate from QMOF (not CoRE MOF 2019) which further indicates that WS24 includes structures that are not strictly from CoRE MOF 2019.

Figure S.10 compares their labels for the 142 entries showing that there are only 78.9% matching labels. The prompts were improved to help eliminate some common error patterns that arise from the LLM ignoring parts of the extraction prompt. Additionally, few-shot examples were added to improve the extraction matching performance between WS24 and LLM labels. Figure S.11 shows slight improvement in the Ansari et al. 2024 benchmark (86.8% accuracy), whereas figure S.12 shows a significant improvement when comparing LLM extractions with WS24 labels (88.2% matching labels). The improved prompt also helped increase the number of labels extracted from the 363 common entries from 142 to 195 (37% more entries). Therefore, the new prompts are used for water stability extraction instead of the original CoV prompts. Figures S.13, S.14 and S.15 show the improved prompts for each of the 3 CoV functions, respectively. Figure S.16 shows the water stability rules adopted from Ansari et al. 2024.[7] Figures S.18 and S.17 show examples of water stability extraction.

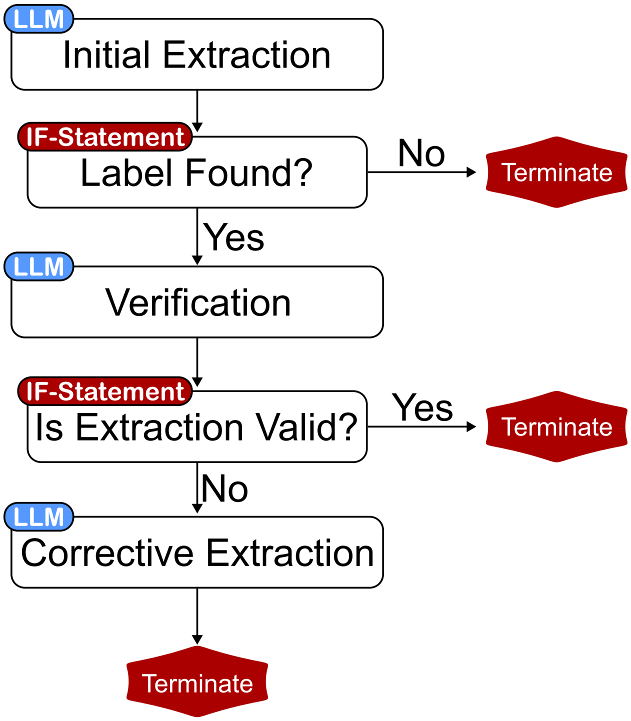

Figure S. 8. Adjusted Chain of Verification (CoV). This adjusted CoV is adopted from Ansari et al. 2024.[7] The major difference is the use of structured LLM output and programming flow control IF statements to determine the next action.

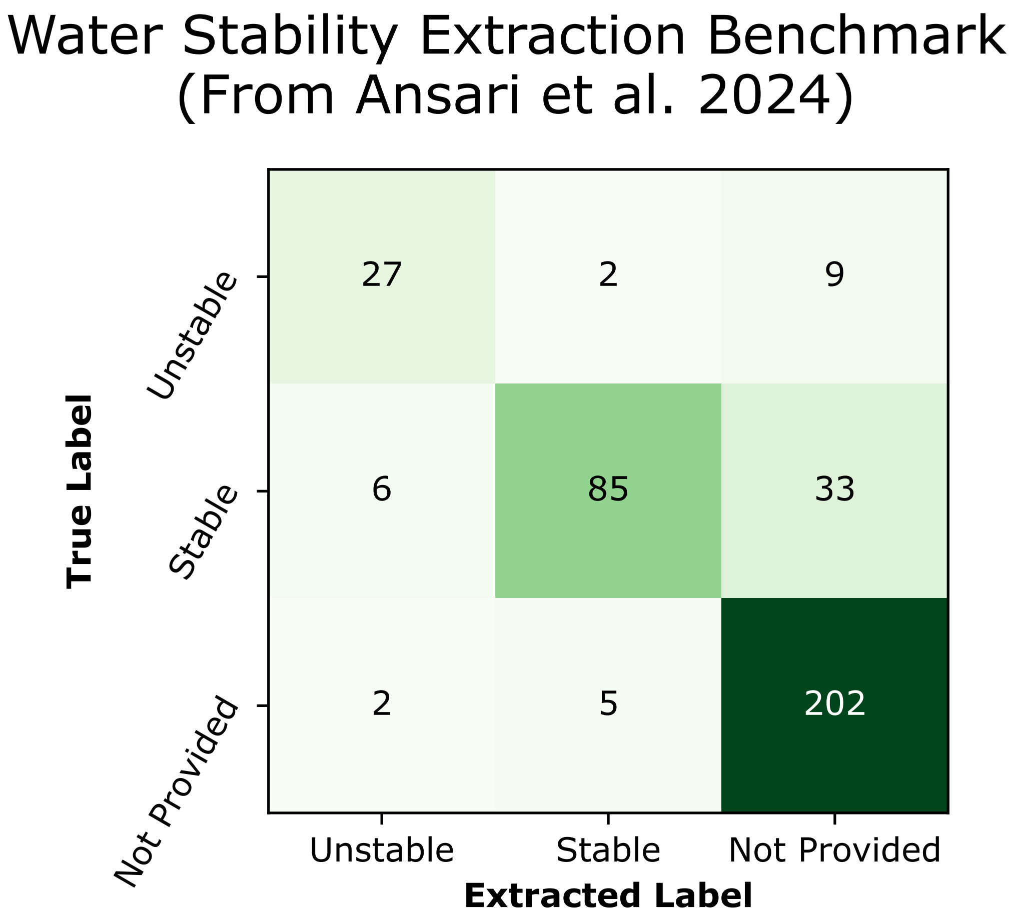

Figure S. 9. Simplified Chain of Verification (CoV) Benchmark Confusion Matrix. The benchmark was performed on the 371 data points from Ansari and Moosavi, 2024[7] by passing the names to the extraction workflow and comparing the extracted labels with true labels provided. The simplified CoV workflow achieved an accuracy of 84.6%.

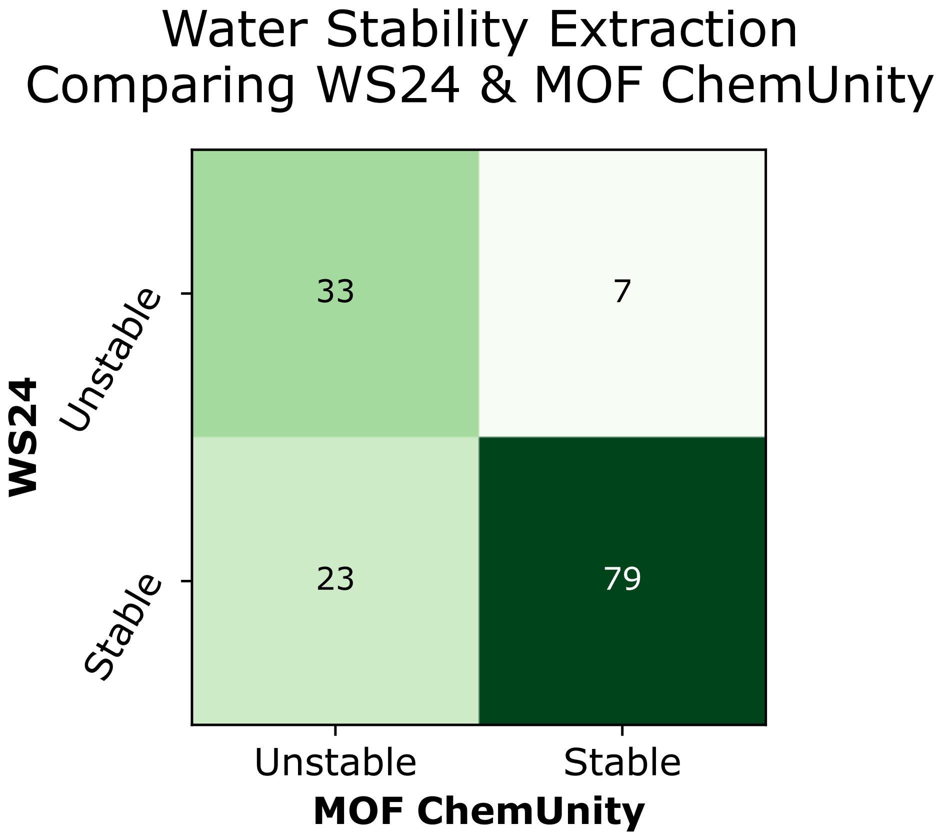

Figure S.10. Simplified CoV Extractions vs. WS24 labels. The confusion matrix compares the labels of the 142 common entries between the simplified CoV extraction (MOF ChemUnity) and the labels from WS24. There is 78.9% matching labels.

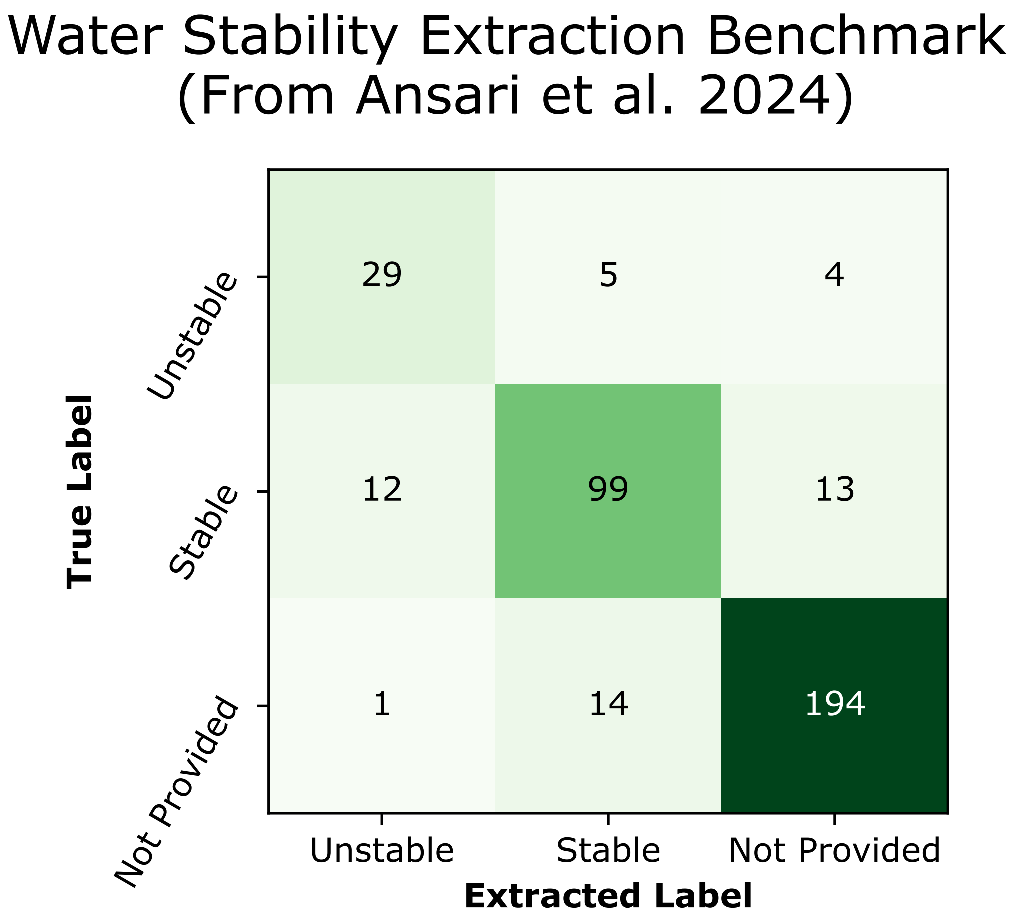

Figure S. 11. Benchmark Results for Improved CoV Prompts. The results on Ansari et al. 2024[7] benchmark using improved CoV prompts show slight improvement with 86.8% accuracy.

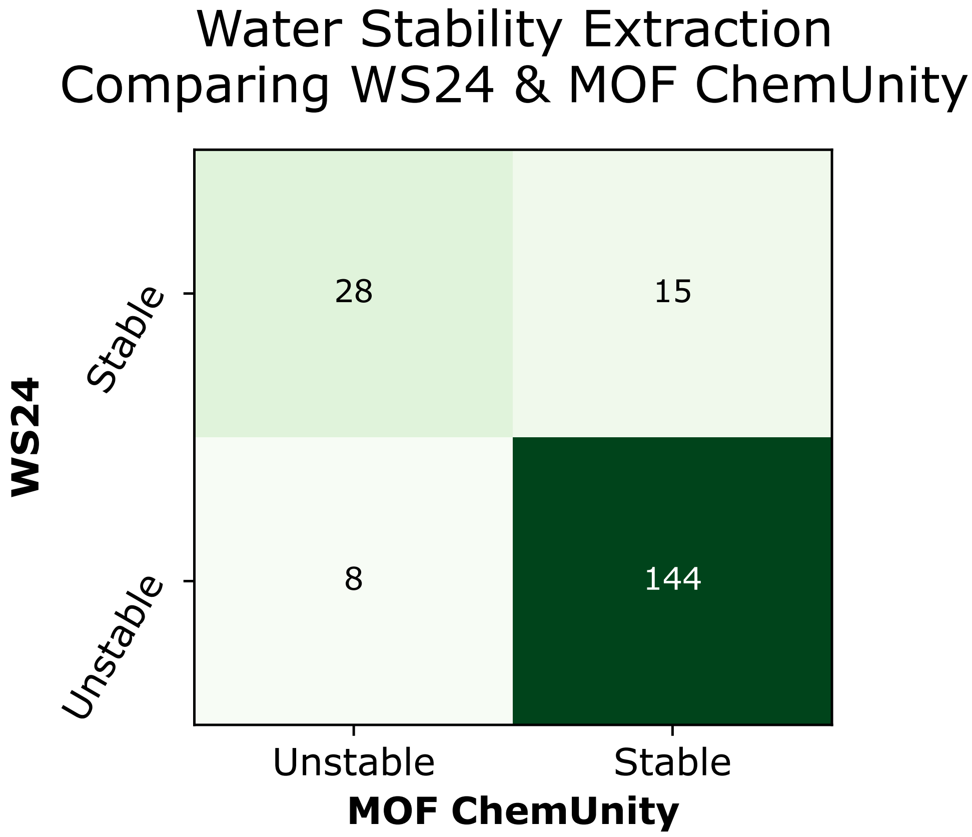

Figure S. 12. Comparing Improved CoV Extractions with WS24. The confusion matrix shows significant improvement with 88.2% of extracted labels matching WS24 labels for 195 entries.

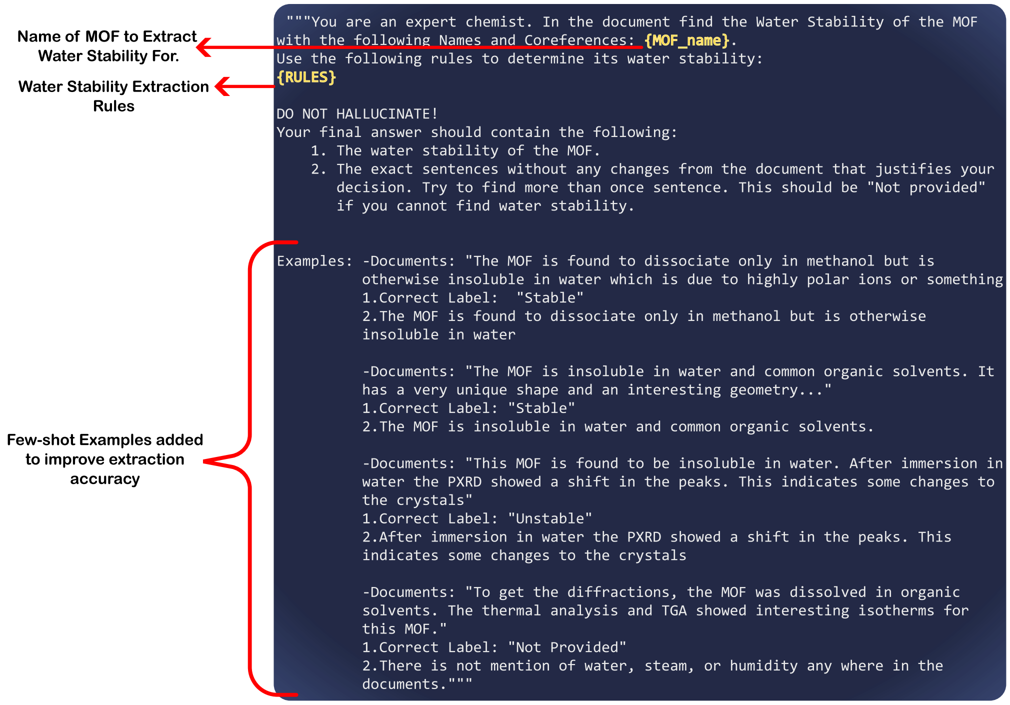

Figure S. 13. Improved Initial Extraction Prompt for Water Stability. This prompt was improved and used in the first step of the Chain of Verification (CoV) which is initial extraction. It was improved by adding few-shot examples.

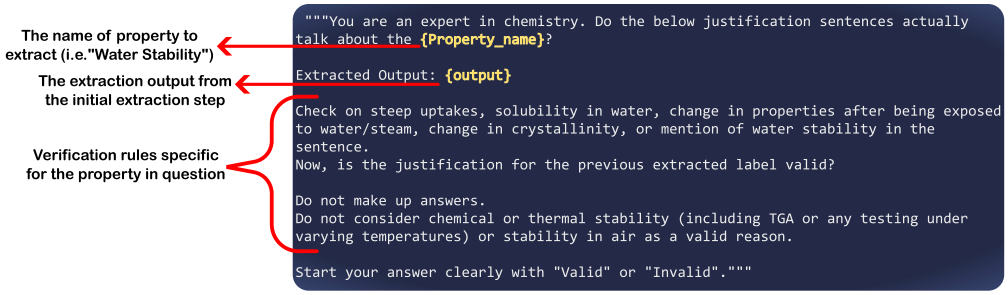

Figure S. 14. Improved Verification Prompt for Water Stability. This prompt was improved and used in the second step of the Chain of Verification (CoV) which is verification. It was improved by making the instructions fit the simplified CoV used in this work.

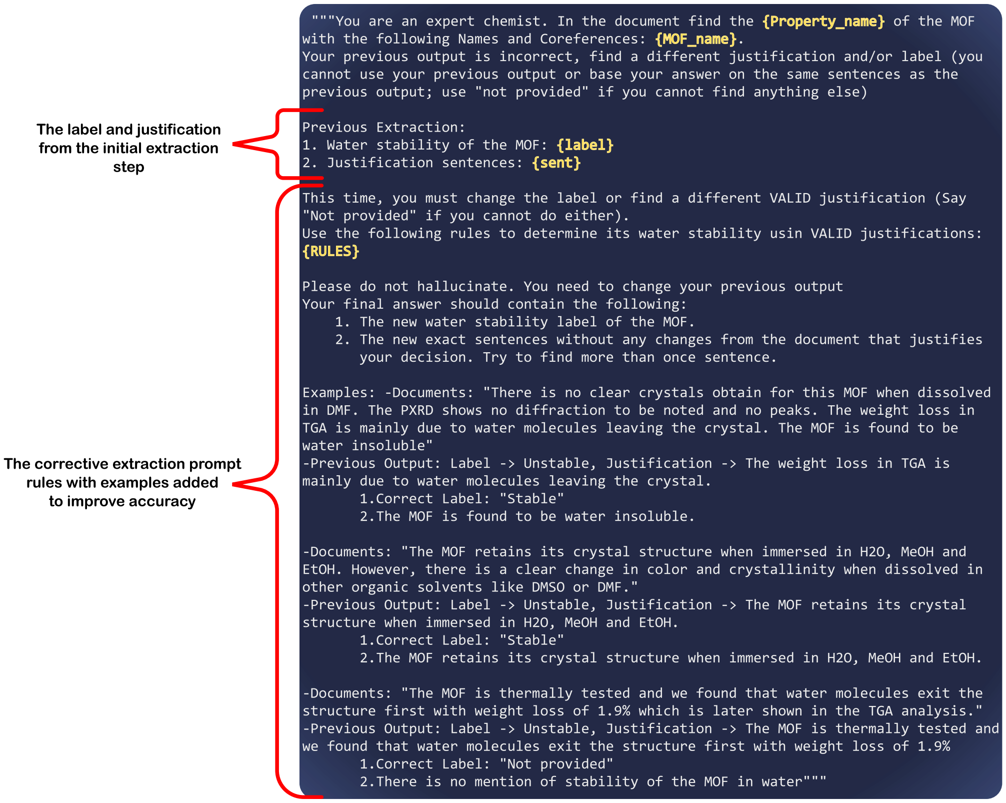

Figure S. 15. Improved Corrective Extraction Prompt for Water Stability. This prompt was improved and used in the last step of the Chain of Verification (CoV) which is corrective extraction. It was improved by making the instructions fit the simplified CoV used in this work. Additionally, examples were added to improve the accuracy of the extraction.

```
 RULES_WATER_STABILITY = """
There are only 2 options for water stability (Stable, Unstable):
1. Stable:  -retains properties/porous structure/crystallinity after exposure to
             water/steam/humidity.
            -Insoluble in water or an aqueous solution.
            -Water adsorption isotherm has a steep uptake.
            -Good cycling performance.

2. Unstable: -The MOF will decompose/change properties/change crystal structure after
              exposure to water/steam/humidity
             -Soluble or patrially soluble in water or an aqueous solution.

3. Not provided: -No information provided for either label
                 -Any thermal analysis or thermal reactions are not water stability.
                 -In-Air Decomposition or Instability of crystal unless humidity is
                  mentioned
                 -Any mention of TGA or TG or temperature is not considered water
                  stability even if water molecules are involved
                 -Information about the interaction with Organic solvents other than
                  including water"""
```

Figure S. 16. Revised Water Stability Rules. These rules were adopted from Ansari et al. 2024.[7] It was improved by making the rules more concise and better formatted. It also suggests using 2 labels (Stable and Unstable) instead of 3 labels (Stable, Unstable, Not provided).

```
You are an expert chemist. In the document find the Water Stability of the MOF with the
following Names and Coreferences: [{((MeCN)Cu)(m3-I)}2{Cu(m-I)}2(bphpyh)2]n<|>compound 6
Use the following rules to determine its water stability:
There are only 3 options for water stability:
1. Stable:  -retains properties/porous structure/crystallinity after exposure to
             water/steam/humidity.
            -Insoluble in water or an aqueous solution.
            -Water adsorption isotherm has a steep uptake.
            -Good cycling performance.

2. Unstable: -The MOF will decompose/change properties/change crystal structure
...

DO NOT HALLUCINATE!
Your final answer should contain the following:
    1. The water stability of the MOF.
    2. The exact sentences without any changes from the document that justifies your
       decision. Try to find more than once sentence. This should be "Not provided" if you
       cannot find water stability.
```

```
Reading to find the Water Stability of [{((MeCN)Cu)(m3-I)}2{Cu(m-I)}2(bphpyh)2]n<|>compound 6
specifically

Water Stability = Unstable
Justification: Compounds 1–6 are relatively air and moisture-stable, and insoluble in common
organic solvents such as CH2Cl2 and MeCN, and slightly soluble in DMF and DMSO.
```

```
You are an expert in chemistry. Do the below justification sentences actually talk about
the Water Stability of the [{((MeCN)Cu)(m3-I)}2{Cu(m-I)}2(bphpyh)2]n<|>compound 6?

Extracted Output: Water Stability = Stable ; Justification: Compounds 1–6 are relatively
air ...

...

Do not consider chemical or thermal stability (including TGA or any testing under varying
temperatures) or stability in air as a valid reason.

Start your answer clearly with "Valid" or "Invalid".
```

```
Invalid, ...
```

```
You are an expert chemist. In the document find the {Property_name} of the MOF with the
following Names and Coreferences: {MOF_name}.
Your previous output is incorrect, find a different justification and/or label (you cannot
use your previous output or base your answer on the same sentences as the previous output;
use "not provided" if you cannot find anything else)

Previous Extraction:
1. Water stability of the MOF: Unstable
2. Justification sentences: Compounds 1–6 are relatively air ...

This time, you must change the label or find a different VALID justification (Say "Not
provided" if you cannot do either).
Use the following rules to determine its water stability usin VALID justifications:
{RULES}

Please do not hallucinate. You need to change your previous output
Your final answer should contain the following:
    1. The new water stability label of the MOF.
    2. The new exact sentences without any changes from the document that justifies your
decision. Try to find more than once sentence.
```

```
Reading to find the Water Stability of [{((MeCN)Cu)(m3-I)}2{Cu(m-I)}2(bphpyh)2]n<|>compound 6
specifically

Water Stability = Not Provided
Justification: Not Provided
```

Figure S. 17. Example of Water Stability Extraction Using CoV In this example, the LLM's initial extraction was that the MOF is "unstable" while the evidence does not imply instability. Therefore, the LLM invalidated the extraction. Then, it requests to find a better justification or a better label which it could not find. The final output is "Not Provided" since LLM could not find evidence that strongly aligns with the rules provided.

```
You are an expert chemist. In the document find the Water Stability of the MOF with
the following Names and Coreferences: MIL-146(Li)<|>Li4[C12H6N2(CO2)4]<|>
MIL-146(Li)[H2O].

Use the following rules to determine its water stability:
There are only 2 options for water stability (Stable, Unstable):
1. Stable:  -retains properties/porous structure/crystallinity after exposure to
             water/steam/humidity.
            -Insoluble in water or an aqueous solution.
            -Water adsorption isotherm has a steep uptake.
            -Good cycling performance.

2. Unstable: -The MOF will decompose/change properties/change crystal structure
              after exposure to water/steam/humidity
             -Soluble or patrially soluble in water or an aqueous solution.

3. Not provided: -No information provided for either label
                 -Any thermal analysis or reactions are not water stability.
                 -In-Air Decomposition or Instability of crystal unless humidity is
                  mentioned
                 -Any mention of TGA or TG or temperature is not considered water
                  stability even if water molecules are involved
                 -Information about the interaction with Organic solvents other
                  than including water

DO NOT HALLUCINATE!
Your final answer should contain the following:
    1. The water stability of the MOF.
    2. The exact sentences without any changes from the document that justifies
       your decision. Try to find more than once sentence. This should be "Not
       provided" if you cannot find water stability.
```

LLM

```
Reading to find the Water Stability of MIL-146(Li)<|>Li4[C12H6N2(CO2)4]<|>MIL-146(Li)[H2O]
specifically

Water Stability = Stable
Justification: The hydration–dehydration process is fully reversible and reproducible as shown in
Fig. 2. The material is able to reversibly bind water without any loss of crystallinity of the
framework. This offers the possibility of the synthesis of 'lightweight' lithium organic
frameworks that show a high gravimetric uptake of guests with strong binding towards a
coordinatively unsaturated lithium centre.
```

```
You are an expert in chemistry. Do the below justification sentences actually talk
about the Water Stability of the MIL-146(Li)<|>Li4[C12H6N2(CO2)4]<|>MIL-146(Li)
[H2O]?

Extracted Output: Water Stability = Stable ; Justification: The hydration–
dehydration process ...

Check on steep uptakes, solubility in water, change in properties after being
exposed to water/steam, change in crystallinity, or mention of water stability in
the sentence.
Now, is the justification for the previous extracted label valid?

Do not make up answers.
Do not consider chemical or thermal stability (including TGA or any testing under
varying temperatures) or stability in air as a valid reason.

Start your answer clearly with "Valid" or "Invalid".
```

LLM

```
Valid, ...
```

CSV

Figure S.18. Another example of water stability extraction. The improved implementation of CoV enables more consistent validation. Initially, the LLM successfully found a justification for the stability of the MOF. The verification step validated the extraction and proceeded to generate the output.

4 Application Extraction

To extract the intended application of each MOF as described by the authors, a different prompt and LLM call were used. This step used the same workflow as the general property extraction. Figures S.19 and S.20 show examples of application extraction. The application extraction workflow uses a different structured output to capture more useful information. Table S.4 shows the fields of the structured output for this workflow. Similar to the general property extraction workflow, this step uses the exact same algorithm (see algorithm 1) to filter and standardize application names. Table S.5 shows the filter dictionary used for this workflow. Lastly, table S.6 shows the applications removed using *"Remove"* key similar to properties.

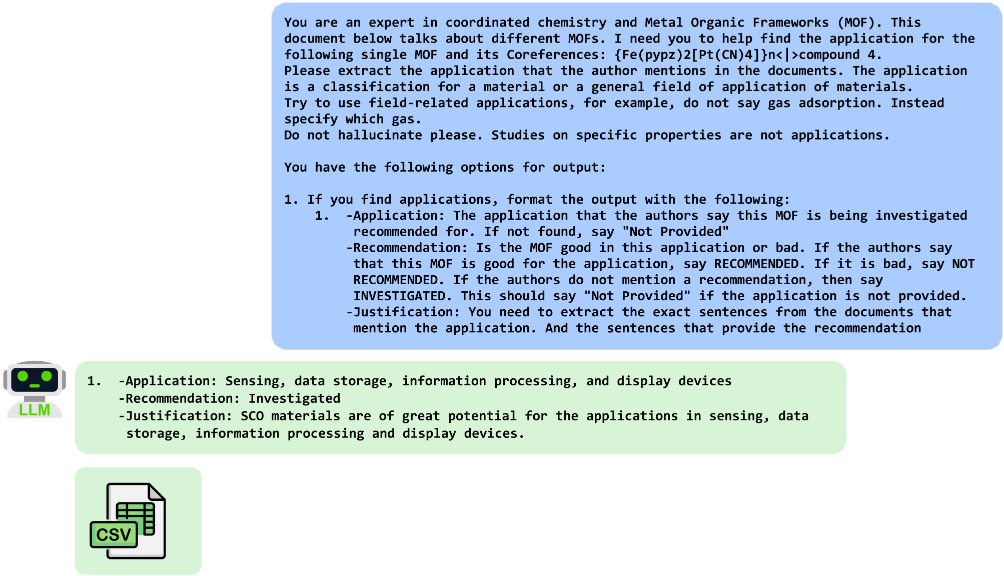

Figure S. 19. Application extraction example. In this example, the LLM identified the application that the MOF is being investigated for but was not necessarily recommended.

```
You are an expert in coordinated chemistry and Metal Organic Frameworks (MOF). This
document below talks about different MOFs. I need you to help find the application for the
following single MOF and its Coreferences: Er-DADQ.
Please extract the application that the author mentions in the documents. The application
is a classification for a material or a general field of application of materials.
Try to use field-related applications, for example, do not say gas adsorption. Instead
specify which gas.
Do not hallucinate please. Studies on specific properties are not applications.

You have the following options for output:

1. If you find applications, format the output with the following:
    1.  -Application: The application that the authors say this MOF is being investigated
         recommended for. If not found, say "Not Provided"
        -Recommendation: Is the MOF good in this application or bad. If the authors say
         that this MOF is good for the application, say RECOMMENDED. If it is bad, say NOT
         RECOMMENDED. If the authors do not mention a recommendation, then say
         INVESTIGATED. This should say "Not Provided" if the application is not provided.
        -Justification: You need to extract the exact sentences from the documents that
         mention the application. And the sentences that provide the recommendation
```

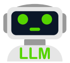


```
1.  -Application: CO2 absorption
    -Recommendation: Recommended
    -Justification: "The chelating-amino groups lie in channels enhancing the CO2 and RhB
     adsorptions."
     "CO2 with higher polarizability can interact well with desolvated Er-DADQ which is mainly
     due to the chelating-amino groups which can have strong interactions with the CO2 molecules
     as well as the open metal sites in the framework."

2.  -Application: Dye separation (RhBâ€“MO and RhBâ€“CV mixtures)
    -Recommendation: Recommended
    -Justification: "The functionalized channels show a good separation of RhBâ€“MO and RhBâ€“CV
     mixtures, as well as CO2 absorption."
     "The results indicate clearly that Er-DADQ is a good material for the separation of the RhB
     MO and RhB/CV mixtures."

3.  -Application: Lewis acid catalysis for cyanosilylation of aldehydes and ketones
    -Recommendation: Recommended
    -Justification: "The metal ions act as strong Lewis acid sites for catalyzing the
     cyanosilylation of aldehydes and ketones in a very short reaction time with no-solvent
     reactions."
     "Er-DADQ showed more than 99% conversions in the cyanosilylation of benzaldehyde and 4
     chlorobenzaldehyde in only one hour."
```

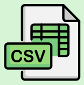

Figure S. 20. Multiple applications extraction example. For this MOF, the authors discussed multiple recommended applications which were extracted.

Table S. 4. Structured Output for Application Extraction The fields of the structured output for application extraction. These are provided as a Pydantic object to the structured output API.

Field Name	Description
Application	The name of the application extracted
Recommendation	Whether the authors indicate the MOF is recommended or not recommended for this application
Justification	The quote from the publication that contains the extracted information along with the recommendation

Table S. 5. Applications Filter Dictionary. This filter dictionary is used with to standardize and filter applications similar to the properties filter.

Application	Mapped Synonyms
Gas Adsorbent Material	Gas Selectivity, Gas Diffusion, Mixed Gas Separation, Gas Mixture Separation, Oxygen Adsorption, N_2/O_2 Separation, Separation Of Gases, Gas Mixture Selectivity, Gas Adsorption Separation, Gas Partitioning, Gas Storage And Purification, Gas Cleaning, Gas Purification, Gas Isolation, Separation Technology, Selective Gas Permeability, Gas Filtering, Gas Mixture Filtration, Gas Adsorption, Gas Storage And Separation, Gas Sorption Applications, N_2 Adsorption, N_2 Uptake, Gas Storage Materials, Acetylene Storage And Separation, C_2H_2 Storage And C_2H_2/CO_2 Separation, N_2/Ar Separation, Air Separation, Gas Storage And Purifications
Heterogeneous Catalysis	Heterogeneous Catalysis
Photocatalysis	Photocatalysis, Photocatalysis Degradation Of, Photocatalytic Degradation Of, Photocatalytic Production, CO_2 Photoreduction, Photoreduction Of

Application	Mapped Synonyms
Electrocatalysis	Electrocatalysis, Electrocatalysis, Electrocatalytic Reduction, Reduction Catalysis, Reduction Reaction Catalysis, Catalysis Of Reduction, Oxidation Catalysis, CO_2 Reduction, CH_4 Reduction, Hydrogen Evolution Reaction, Oxygen Reduction, H_2 Evolution Reaction, O_2 Evolution Reaction, CO_2 Fixation, Water Oxidation
Catalysis	Catalysis, Catalytic Activity, Catalyst, Enzyme Mimic, Organic Synthesis Catalysis, Catalytic Reaction, Catalyst Activity, Catalytic Process, Catalyst Efficiency, Catalysis Materials, Reaction Catalysis, Chemical Reaction Catalysis, Asymmetric Catalysis
Water Purification	Water Purification, Water Treatment, Wastewater Filtration, Water Remediation, Water Decontamination, Clean Water Production, Fluoride Removal, Dye Separation, Purification Of Water, Clean Water Treatment, Water Filtration, Water Cleaning, Water Detoxification, Safe Drinking Water, Water Purity, Water Purification Systems, Filtration Of Water, Water Adsorption, Dye Adsorption

Application	Mapped Synonyms
Pollutant Removal	Pollutant Degradation, Pollution Removal, Degradation Of Organic Pollutants, Environmental Remediation, Toxic Compound Degradation, Organic Pollutant Breakdown, Industrial Waste Degradation, Pollutant Breakdown, Pollution Degradation, Organic Degradation, Pollution Detoxification, Pollutant Remediation, Decomposition Of Pollutants, Toxic Waste Treatment, Pollutant Neutralization, Photocatalytic Degradation Of Organic, Degradation Of
Drug Delivery	Drug Delivery, Targeted Drug Delivery, Controlled Drug Release, Therapeutic Delivery, Pharmaceutical Carrier, Bioactive Compound Delivery, Anticancer Drug Delivery, Drug Release System, Medication Delivery, Therapeutic Compound Delivery, Targeted Medication Delivery, Drug Carrier Systems, Pharmaceutical Delivery Systems
Photoluminescence	Photoluminescence, Fluorescence, Light Emission, Photonic Properties, Luminescence, Light Absorption And Emission, Emission Of Light, Light Absorption, Luminescent Materials, Photon Emission, Light Scattering, Photoemission, Photonic Activity, Photoluminescent Materials, Photoluminescence, Luminescent Properties, Blue-Luminescent Materials, Blue-Fluorescent Materials

**Application**	**Mapped Synonyms**
Energy Storage	Energy Storage, Battery Materials, Thermal Energy Storage, Electrochemical Energy Storage, Energy Retention, Energy Harvesting, Fuel Cell Materials, Energy Accumulation, Energy Conservation, Energy Retention Systems, Energy Storage Materials, Energy Management, Energy Storage Technology, Fuel Cells
Gas Sensing	Gas Sensing, Gas Detection, Gas Sensor Applications, Toxic Gas Monitoring, Volatile Organic Compound (VOC) Detection, Environmental Sensing, Industrial Gas Monitoring, CO_2 Sensor, NOx Sensor, SOx Sensor, Detection Of Gases, Gas Monitoring, Gas Sensing Applications, VOC Sensing, Toxic Gas Detection, Gas Analysis
Membrane Technology	Membrane Technology, Membrane Separation, Gas Separation Membranes, MOF-Based Membranes, Water Desalination Membranes, Permeation Membranes, Selective Permeability Membranes, Membrane Materials, Gas Permeability Membranes, Filtration Membranes, Membrane Filtration, Separation Membranes
Cancer Therapy	Cancer Therapy, Oncological Applications, Cancer Treatment, Antitumor Activity, Photodynamic Therapy, Chemodynamic Therapy, Therapy For Cancer, Anticancer Therapy, Tumor Treatment, Cancer Therapeutics, Treatment Of Tumors, Cancer Management

Application	Mapped Synonyms
Sensors	Chemical Sensors, Biosensors, Optical Sensors, Electrochemical Sensors, MOF-Based Sensing Applications, Detection Sensors, Sensor Technology, Analytical Sensors, Sensing Materials, Sensor Applications, Sensing Devices, Fluorescent Sensing, Sensors, Pressure Sensors, Luminescent Sensors, Detection Of Nitroaromatic Explosives, Detection Of Nitrobenzene, Nitrobenzene Detection, Detection Of Acetone, Detection Of Fe^{3+} Ions, Sensing Of Fe^{3+} Ions, Luminescence Sensing, Sensing Of Small Molecules, Luminescent Probes, Cation Sensing, Magnetic Sensor Devices, Sensing Applications
Heavy Metal Removal	Lead Removal, Mercury Removal, Cadmium Removal, Arsenic Removal, Metal Ion Adsorption, Heavy Metal Detoxification, Removal Of Heavy Metals, Heavy Metal Filtration, Metal Ion Removal, Detoxification Of Metals, Metal Contamination Removal, Heavy Metal Removal, Arsenic Removal, Lead Removal
Solar Energy Harvesting	Solar Cells, Photovoltaics, Light Absorption, Solar Energy Conversion, Solar Panel Materials, Harvesting Solar Energy, Solar Light Conversion, Solar Energy Materials, Solar Light Absorption, Solar Energy Systems
Self-Healing Materials	Self-Repairing Materials, Autonomous Healing Materials, Structural Repair Materials, Self-Reinforcing MOFs, Self-Healing Systems, Self-Healing Polymers, Materials With Self-Repair Capability, Self-Fixing Materials

**Application**	**Mapped Synonyms**
Data Storage	Data Retention, Information Storage, Memory Storage, Data Preservation, Digital Storage, Data Storage Capacity, Data Archiving, High-Density Storage, Data Storage Systems, Retention Of Information, Digital Data Preservation, Data Storage Materials, Data Accumulation
Optical Devices	Optical Materials, Nonlinear Optical Materials (NLO), Optical Devices, Nonlinear Optical Properties (NLO), Nonlinear Optics (NLO), Optoelectronic Devices, Nonlinear Optical (NLO) Applications, Optics, Optoelectronic Properties, Optical Applications
Magnetic Refrigeration	Magnetic Refrigeration, Cryogenic Magnetic Refrigeration, Cryogenic Magnetorefrigeration
Magnetic Material	Magnetism, Magnetic Material, Magnetic Properties
Electrical Conductivity	Electrical Conductivity, Supercapacitors, Semicapacitors, Capacitors, Ferroelectricity, Ferroelectric Materials, Supercapacitor
Small Molecule Capture	Separation Of Isomers, Molecular Separations, Small Molecular Separations, Small Molecular Storage, Small Molecule Storage And Separation, Molecular Storage And Separation
Molecular Magnets	Molecular Magnets, Molecular Magnetism

Application	Mapped Synonyms
Light Emitting Diodes	Light Emitting Diodes (LEDs), Blue-Light Emitting Diodes, Blue-Light Emitting Material, White-Light Emitting Material, Blue-Light Emitting Diodes, White-Light Emitting Diodes, Light Emitting Properties, White-Light Emission, Solid-State Lighting
Iodine Capture	I_2 Adsorption, I_2 Capture, I_2 Separation, Iodine Adsorption, Iodine Capture, Iodine Separation
Remove	Multiferroics, O_2/N_2 Separation, Ion Exchange, CO_2 Selectivity, Anion Separation, Adsorption, Storage, Sorption Material, Separations, Separation, Adsorptive Material, Electrochemical Fields, Luminescence Tuning, Chiral Sorption, Ethanol Adsorption, N-Butane Adsorption, Biomedical Applications, Photochemical Applications, Protein Adsorption, Solvent Storage And Separation, Adsorption And Separation, Solvent Adsorption And Separation, Molecular Adsorption/Separation, Adsorption And Separation

Table S. 6. Applications Removed. Any extracted application with a name similar to the synonyms in this table was removed.

Applications Removed		
Multiferroics	O_2/N_2 Separation	Ion Exchange
CO_2 Selectivity	Anion Separation	Adsorption
Storage	Sorption Material	Separations
Separation	Adsorptive Material	Electrochemical Fields
Luminescence Tuning	Chiral Sorption	Ethanol Adsorption

N-Butane Adsorption	Biomedical Applications	Photochemical Applications
Protein Adsorption	Solvent Storage And Separation	Adsorption And Separation
Solvent Adsorption And Separation	Molecular Adsorption/Separation	Adsorption And Separation

5 Synthesis Procedure Extraction

To extract the synthesis procedures of each MOF as described by the authors, a differet prompt and LLM call were used. This step used the same workflow as the general property extraction. Figure shows an example of synthesis procedure extraction. This workflow uses a custom structured output to capture more useful information. Table S. 7 shows the fields of the structured output for this workflow.

Table S. 7. Structured Output for Synthesis Extraction The fields of the structured output for synthesis extraction. These are provided as a Pydantic object to the structured output API.

**Field Name**	**Description**
Metal Precursor	The name of the metal precursor(s) used in the synthesis.
Solvent	The solvent(s) used in the synthesis process.
Temperature	The temperature at which the synthesis was conducted.
Reaction Time	The duration for which the synthesis reaction was carried out.
Synthesis Procedure	A detailed step-by-step summary of how the MOF was synthesized.
Additional Conditions	Any other experimental parameters mentioned, such as pH, pressure, additives, or special conditions.
Justification	The exact sentences from the provided text that describe the synthesis procedure.

6 Benchmarks

6.1 Matching Benchmark Results

A hand-labeled benchmark was constructed from 60 papers that are referenced by MOFs in the Cambridge Structural Database (CSD).[4]These papers were randomly sampled from the corpus, without handpicking specific cases. While the benchmark set includes only 60 papers ($< 0.1\%$ of the full text-mined corpus), this sample size is in line with other studies that rely on manually labeled data.[10,11] We do not expect the accuracy estimates to change substantially with larger test sets. Performance was based off of two metrics, accuracy and yield. If the output correctly matches the inputted CSD Code to its MOF name in the paper, accuracy is scored 1/1, and yield is score 1/1. If a name is matched for an inputted CSD Code, but it is incorrect, the accuracy is scored 0/1 and the yield 1/1. If the inputted CSD Code is not matched to any MOF name, the accuracy is score 0/0, and yield 0/1. From the 60 papers, there were 108 associated CSD Codes. The model made predictions for 106 of them, and was correct for 101. This results in a yield of 98%, and accuracy of 94%. Note, the condition to satisfy a "correct" name match was that at least one extracted co-reference must exactly match a correct co-reference in the paper. This includes cases where a MOF has undergone post-synthetic modifications, such as nanoparticle loading, and the MOFs name is altered to indicate this modification. For example if the MOF "UiO-66" was loaded with platinum, the matching agent must output the name "Pt@UiO-66" as a co-reference to be deemed correct. The matching agent was also tested with examples of isoreticular MOFs (where one paper reports several different MOFs with the same topology but changes in linker length, functionality, or metal node identity and therefore has a unique crystal structure). For example, the matching agent correctly differentiates between Cd2Cl2(Me-3py-trz-pba)2 and Cd2I2(Me-3py-trz-pba)2 during benchmarking.

6.2 General Property Extraction Results

A hand-labeled benchmark was constructed from 20 papers that are referenced by MOFs in CSD. From these papers, 90 experimental properties were manually extracted by readers. If the model extracts a property that the reader also identified, the yield is scored 1/1. If that property's value was correct, the accuracy is scored 1/1. If a property was extracted by the reader, but not extracted by the LLM, the yield is scored 0/1, and accuracy scored 0/0. From these 20 papers, the model extracted 84 properties, of which 80 had accurate values. This results in a yield of 94% and accuracy of 89%

6.3 Synthesis Procedure Extraction Results

A hand-labeled benchmark was constructed from 20 papers that are referenced by MOFs in CSD. From each paper, one MOF's synthesis procedure was manually extracted by readers. From each synthesis procedure, 6 pieces of information were extracted: Metal Precursor, Linker, Solvent, Temperature, Reaction Time, Synthesis Procedure Summary, and Additional Conditions. The LLM returns a structured output matching these 6 fields. If the model output matches the reader's manual extraction for a given field, the accuracy is scored 1/1, and the yield is scored 1/1. If the model incorrectly outputs a value for a given field, the accuracy is scored 0/1, and the yield 1/1. If the model returns "not provided" but the reader was able to find and extract that piece of info from the text, the yield is scored 0/1, and the accuracy is scored 0/0. Across the 20 papers, the model made predictions for all 120 fields, and was correct for all of them. This results in a yield and accuracy of 97.5%.

7 Workflow Information

In this work, a workflow refers to an LLM provided with a specific prompt and the processing of the LLM output in the final tabulated output. For example, the general properties extraction workflow includes the LLM call (with a specific prompt) and the filtering step.

This section will focus on the setting and options used by the LLM call for each step.

7.1 Retrieval-Augmented Generation Information

Retrieval-augmented generation (RAG) techniques were implemented to improve the extraction outcome of the LLM workflows. In this work, the documents were split into chunks and converted into vector stores using OpenAI *"text-embedding-ada-002"* model[12] (see SI section 7.5 for more details). The retrieval prompt, figure S.21, consists of a question - input/question from the user - and context - the chunks from the vector store. At first, all relevant chunks are added to the retrieval prompt context. If the prompt exceeds the LLM context window size, the least relevant chunk is removed from it. This iterative process continues until the LLM accepts the prompt. Table S.8 shows the RAG settings used in this work.

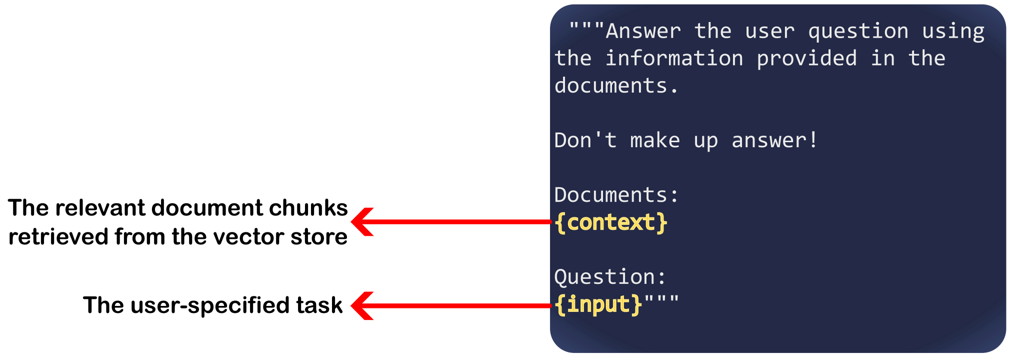

Figure S. 21. Retrieval-augmented Generation QA Prompt. This prompt was used for all extraction tasks including: matching, property extraction, and application extraction. It takes *"context"* and *"input"* as variables. Context is replaced with relevant document chunks retrieved from the vector stores. Input is the task specific prompt.

7.2 LLM Information

For all workflows, GPT-4o was the LLM used. Additionally, all LLM calls were made using the structured output API endpoint. This API endpoint provides more accurate parsing than using the prompt alone.[13] Table S.9 shows the LLM settings used by each workflow, as

Table S. 8. RAG Settings for Workflows.

RAG variable	Value	Description
k	9	Initial (Maximum) number of relevant chunks to include
min_k	2	Minimum number of relevant chunks to include
search_type	"similarity"	The similarity algorithm used to find relevant document chunks
fetch_k	50	Number of documents to pass to the similarity algorithm

well as the total cost.

Table S. 9. LLM Model and Temperature.

Workflow	Model Name	Temperature
Matching	GPT-4o	0
Property Extraction	GPT-4o	0.1
Application Extraction	GPT-4o	0.1

8 Selection of the Starting Crystal Structures

To best achieve an accurate one-to-one link between available computational data and the experimental information extracted in this work, the initial list of MOFs was chosen from the union of the Computational-Ready Experimental MOF (CoRE MOF 2019) and the Quantum MOF (QMOF) datasets. These two datasets contain a collection of valuable computational labels such as gas uptakes at low and high pressures, and band gap.[1–3] Note that these databases contain materials that are misclassified as MOFs or have invalid crystal structures. Improving database quality is an ongoing challenge in computational MOF research.[14]

Since crystal information is required for the matching workflow, only entries with a CSD reference code provided were considered. Additionally, each CSD entry is associated with the DOI of the publication in which it was first discovered. This DOI is later used to obtain publisher information and the full text of the publications. Figure S.22 shows a step-by-step flow chart, which highlights the number of MOF entries (CIF) and publications (DOI) after

each step.

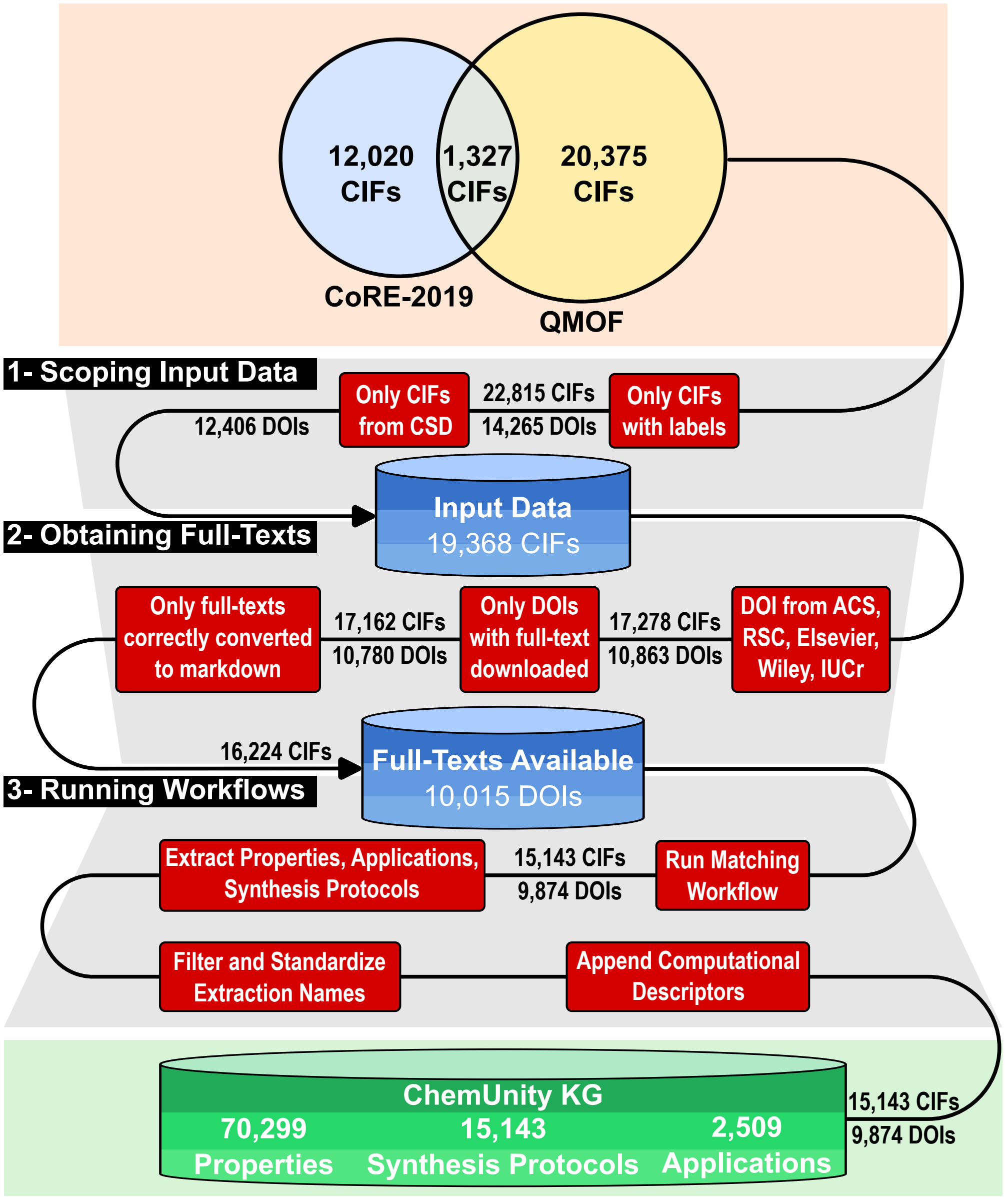

Figure S. 22. Detailed step-by-step flow chart for input data curation All data points considered are from either CoRE MOF 2019 or QMOF. Overall, 66% of all entries with labels from CoRE MOF 2019 or QMOF are included in MOF ChemUnity.

8.1 CoRE MOF 2019 Entries

For this work, we only considered entries in CoRE MOF 2019 that are also in CSD (they have a CSD reference code) and have computed gas uptake labels. Initially, CoRE MOF 2019 contains 12,020 entries.[1] According to the criteria in this work, 8,081 entries are considered

for the input.

8.2 QMOF Entries

Similar to CoRE MOF 2019, the Quantum MOF dataset (QMOF) contains valuable computational labels such as the electronic properties MOFs.[2,3] In this work, only entries that have a CSD reference code associated with them are considered. Similar to before, entries that are missing computed labels are also excluded. Within QMOF, there are 20,375 entries of which 12,352 are considered for the input.[2,3]

8.3 Input Dataset

The input file considered for this work contains the union of QMOF and CoRE MOF 2019 entries that match the criteria. In other words, the input file contains 20,524 entries (1,065 entries common between QMOF and CoRE MOF 2019). In terms of number of publications, the input file consists of 12,406 unique publications (identified by their DOI). Table S.10 shows the breakdown of the data in the input file.

Table S. 10. Breakdown of Input Data. The data was originally obtained from CoRE MOF 2019 and QMOF. Only entries with CSD reference codes were kept.

	Original		Input File	
Dataset	CSD Codes	DOIs	CSD Codes	DOIs
**CoRE MOF 2019**	12,020[15]	-	8,081	4,766
**QMOF**	20,375[16]	10,498	12,352	8,872
**Shared**	1,327	-	1,065	807

9 Obtaining Publications Full-Texts

The input file also contains 12,406 unique DOIs which correspond to publications from different publishers and various journals. The full-texts are obtained using the API provided

by each publisher (except ACS). All processing and analysis of the full texts were carried out in accordance with the TDM licenses of each of the publishers.

9.1 Identification of Publishers

From those DOIs, figure S.23 shows the breakdown of how many correspond to each publisher. For the purpose of this work, only the top 5 publisher were considered since they cover almost 10,863 (88%) of all publications and more than 17,278 unique MOF entries (89%). Upon obtaining these files, 10,780 of the total number files were successfully downloaded (17,162 MOF entries).

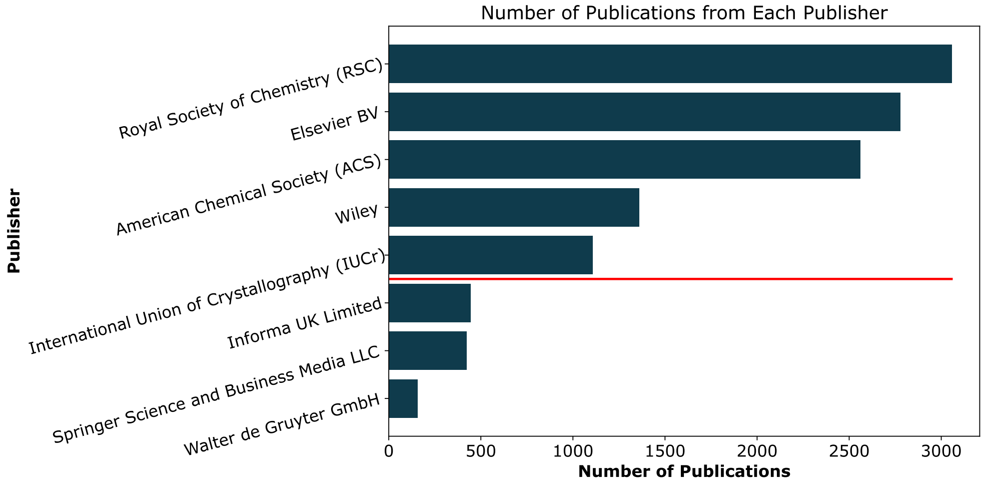

Figure S.23. Number of Publications from Each Publisher. Only the top 5 publishers were considered for this work which amounted to 10,863 publications in total.

9.2 Full-text Formats

Each publisher provides the full texts in different file formats according to their TDM licenses. Table S.11 summarizes the file formats obtained from each publisher.

Table S. 11. File Formats Obtained. Different file formats were provided from each of the publishers as per their text and data mining (TDM) license.

Publisher	File Format	Number of Files Obtained
American Chemical Society (ACS)	XML	2,560
Elsevier	XML	2,780
Royal Society of Chemistry (RSC)	PDF	3,056
Wiley	PDF	1,278
International Union of Crystallography (IUCr)	PDF	1,106

9.3 Converting PDF to Markdown

Prior to processing the files using the LLM workflows, they were first converted to markdown. This applies to both PDF and XML file formats. For PDF files, they were converted to markdown using a local conversion python library called Marker.[17] This is an open-source library that uses various document processing techniques to extract document elements and construct a markdown file. All PDF files were successfully converted to markdowns with varying degrees of accuracy. It was observed that Marker struggles with double column formats as it starts missing some headers in some cases. However, it would maintain the correct flow of the text.

9.4 Converting XML to Markdown

Converting XML files to markdown was more challenging since ACS and Elsevier use different XML tags in their full texts. In the case of ACS, Pub2TEI Python client[18] was used to convert the publisher-specific XML format to a standard (TEI) XML format. A script was then used to extract the text and section headers from XML to generate the markdown file. Pub2TEI was not used with Elsevier because it did not sucessfully convert the XML formats they provided. Therefore, a custom XML parsing script was used to convert Elsevier XML files to markdown. In both cases, converting XML files to markdown format proved to be more difficult because a larger number of files failed to correctly convert to markdown, majority of which were from Elsevier.

9.5 Creating Vector Stores

After converting the full texts to markdowns, there were 9,874 files remaining. In order for the LLM to provide an accurate extraction, the files were not passed whole. Instead, they were split into chunks and converted to individual vector stores. For all files, the embedding model used was OpenAI's *"text-embedding-ada-002"*. Overall, 9,874 vector stores were created.

10 Extracted Dataset

After running the LLM workflows, 70,299 properties and 2,509 applications were extracted for 15,143 MOF entries after removing duplicate CSD reference codes. Below is a deeper analysis of the data.

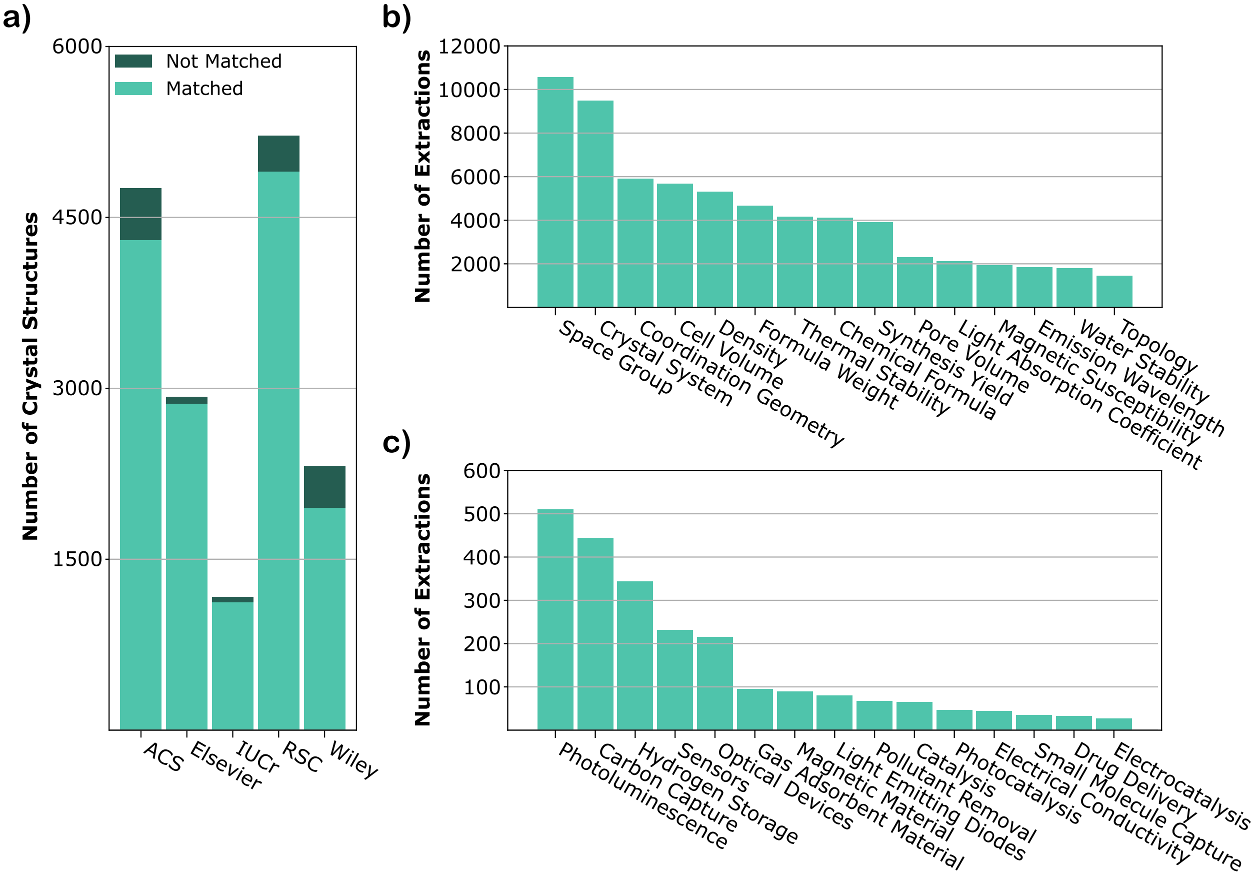

Figure S. 24. Extracted Properties and Applications. (a) The extracted dataset contains 15,143 CSD reference codes matched with MOF names and co-references, which is 93% of all reference codes across all publishers, including American Chemical Society (ACS), Elsevier, International Union of Crystallography (IUCr), Royal Society of Chemistry (RSC), and Wiley. There are 70,299 properties and 2,509 applications extracted using the names and co-references from these papers, where the top 15 extracted properties (b) and applications (c) are shown.

10.1 Analysis of Matching Workflow Output

In total, the workflows matched 15,151 MOFs out of the 16,224 from the input file. Within the matched MOFs, 8 entries did not have unique CSD reference codes and were removed from the dataset, leaving 15,143 MOFs. Figures S.25, S.26, and S.27 provide a breakdown of the data in terms of year, topology and metal types within this dataset, respectively.

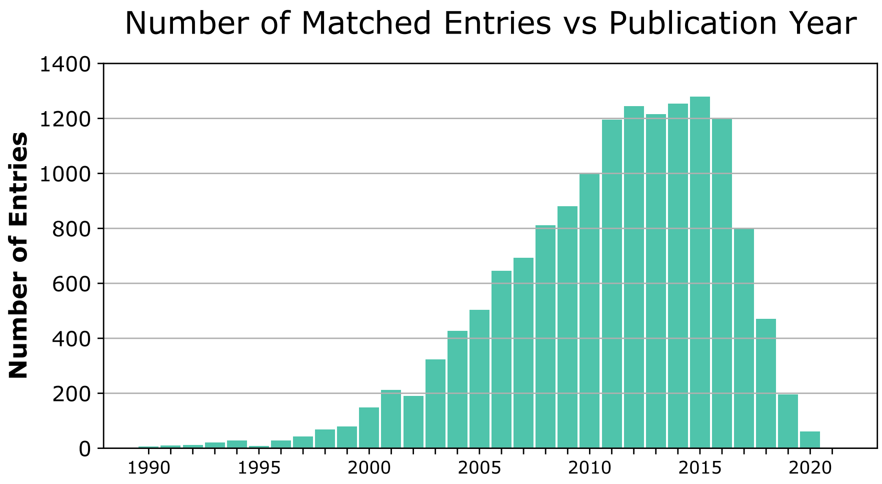

Figure S. 25. Number of Entries per Year. Since both CoRE MOF 2019 and QMOF were used to identify candidate inputs to the workflows, most data points are from the last decade.

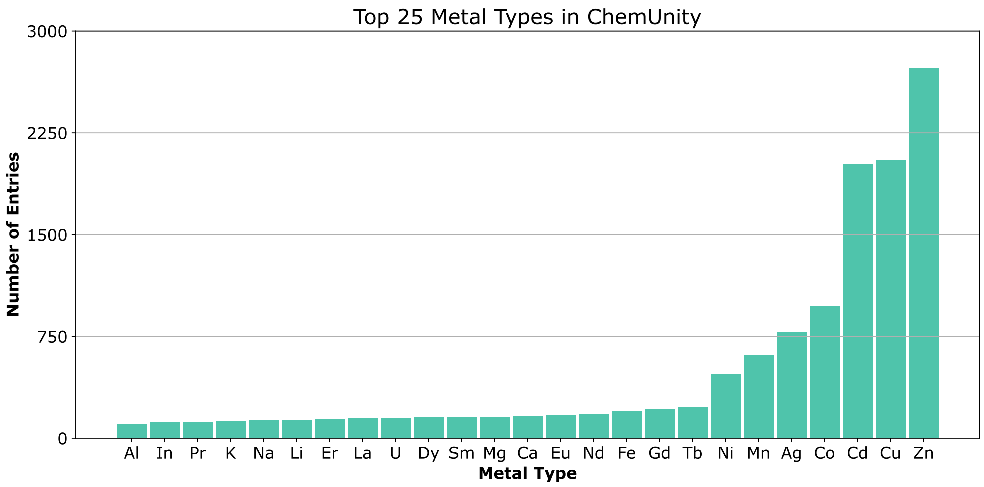

Figure S. 27. Top 25 Metal Types in MOF ChemUnity. Most MOF entries within MOF ChemUnity have Zn, Cu or Cd as the only metal type in their structures.

10.2 Analysis of Property Extraction Workflow

Initially, the general property extraction workflow extracted 154,105 rows in total. These extractions corresponded to 27,220 unique properties. After filtering the output, only 29 unique properties were left (as per table S.2). In total, 70,299 properties remained after the

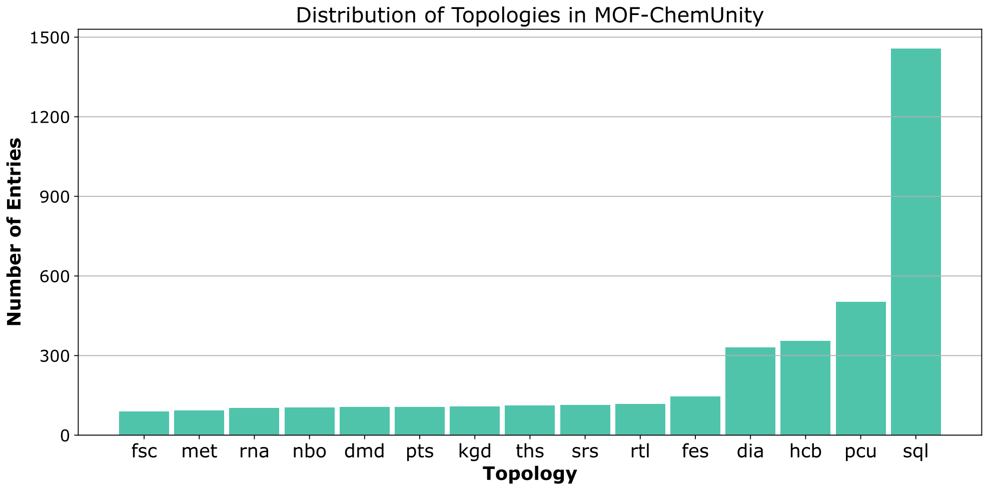

Figure S. 26. Top 15 topologies within MOF ChemUnity. The topologies for MOFs within MOF ChemUnity were obtained from MOFid's where available.[19]

filter. To further verify the filter, table S.12 shows the original property names that were mapped to each of selected properties extracted.

Table S. 12. Filtered Properties and Their Original Name Demonstrates the original property names matched with each of the selected extracted properties.

**Filter Property Name**	**Original Property Name**
Space Group	Space Group, Crystallographic Space Group, Superspace Group, Space Group, Crystallization Space Group, Crystal Space Group, Crystallographic Data - Space Group, Crystal Space Group, Space Group (1P), Space Group (1M), Crystallographic Space Group, Space Group of 2
Crystal System	Crystal System, Crystal System, Crystal System Type

Cell Volume	Cell Volume, Channel Volume, Cell Volume, Unit Cell Volume Change, Unit Cell Volume, Crystal Volume, Unit Cell Volume (1), Unit Cell Volume (1d), Unit Cell Volume (LS), Unit Cell Volume Change, Crystal Cell Volume, Unit Cell Volume (1HT), Unit Cell Volume (3HT), Cage Volume, Unit-Cell Volume, Crystal System Volume, Unit-Cell Volume
Density	Density, Density (Calculated), Z, Calculated Density, Calculated Density, Dc, D Calc, Density (Calculated), Density, Calculated, D(Calcd), ρ_{calcd} (Calculated Density), D Calcd., Density, D Calcd, Dcalc, Density Calculated, Dcalcd, DCalc, Dcal, ρ_{calc}, Dcalcd., Calculated Density, Density Calculated, D Calc., Dcalc (Calculated Density), d Calcd

10.3 Analysis of Application Extraction Workflow

The application extraction workflow initially extracted 15,962 rows, which consists of 3,294 unique applications. The filtered data set consists of 31 applications and 2,509 rows in total. Table S.13 shows the mapping of extracted applications to filter keys.

Table S. 13. Filtered Applications and Their Original Name Demonstrates the original application names matched with each of selected extracted applications.

Filter Application Name	Original Application Name
Photoluminescence	Blue Fluorescent Materials, Blue Luminescent Materials, Luminescence, Photoluminescent Material, Luminescent Materials, Luminescence Materials, Fluorescent Materials, Photoluminescence, Luminescent Properties, Luminescent Material, Photoluminescent Materials, Blue-Fluorescent Materials, Photoluminescent Devices, Fluorescent Material, Fluorescence, Luminescent Properties, Phosphorescent Materials, Photoluminescence (PL), NIR Luminescent Materials, Luminescence Properties, Luminescent Sensor Materials, Luminescent Sensory Materials, Luminescent Property, Solid Luminescent Materials, Fluorescence Material, Hybrid Luminescent Materials, Luminescent Sensor Material, Photoluminescence Materials, Fluorescence Materials, Blue-Light Luminescent Materials, Green Luminescence Materials, Luminescent Material Field, Luminescent Sensing Materials, Blue-Fluorescent Materials, Green-Fluorescent Materials, Tunable Luminescent Material, Solid-State Photoluminescent Materials, Photoluminescent and Magnetic Materials, Photocatalytic Properties, Purple-Fluorescence Materials, Efficient Luminescent Materials, Blue-Luminescent Materials

Carbon Capture	CO_2 Sorption, Carbon Dioxide (CO_2) Adsorption, Carbon Dioxide Storage, CO_2 Uptake, Carbon Dioxide Capture, CO_2 Capture and Separation, CO_2 Adsorption, CO_2/N_2 Separation, CO_2/CH_4 and CO_2/N_2 Separation, CO_2 Capture, CO_2 Storage, Carbon Dioxide (CO_2) Capture, Selective Carbon Dioxide Adsorption, CO_2 Separation, Carbon Dioxide Adsorption, CO_2 Gas Storage, CO_2 Capture, CO_2 Gas Adsorption, CO_2/N_2 and CO_2/CH_4 Separation, Carbon Dioxide Capture, CO_2 Adsorption, Carbon Dioxide (CO_2) Storage, CO_2 over N_2 Separation, Carbon Dioxide (CO_2) Capture, CO_2 Sequestration, CO_2 Separation, CO_2 Storage and Separation, Carbon Dioxide Sorption, CO_2 and CH_4 Adsorption, CO_2 Capture and/or Separation, CO_2 and CO Separation, Carbon Dioxide Selective Capture, CO_2 Storage and Sensing, Carbon Dioxide Selective Capture, Carbon Dioxide and Methane Sorption, Carbon Dioxide (CO_2) Sorption, Carbon Dioxide Separation, CO_2/N_2 Gas Separation, CO_2 Absorption, Carbon Capture, CO_2 Adsorption and Storage, CO_2/N_2 and CO_2/H_2 Separation, Carbon Dioxide Uptake, Carbon Dioxide Fixation, CO_2 Adsorption and Separation, Carbon Dioxide (CO_2) Adsorption, CO_2 Gas Selectivity, CO_2 Adsorption and CO_2/N_2 Separation, Selective Carbon Dioxide Capture, CO_2 Adsorption/Separation

Hydrogen Storage	Hydrogen (H_2) Adsorption, Hydrogen Storage, Hydrogen Adsorption, Hydrogen Sorption, Hydrogen (H_2) Storage, Hydrogen Storage and Proton Migration, Hydrogen Absorption, H_2-Storage Applications, Hydrogen Storage and Purification, H_2 Adsorption, H_2 Storage, Hydrogen Separation, H_2 Uptake, H_2 Gas Adsorption, Hydrogen Gas Storage, Hydrogen Storage, Hydrogen Gas Adsorption

Optical Devices	Nonlinear Optical (NLO) Switch, Optics, Optical Devices, Nonlinear Optical Properties, Nonlinear Optical (NLO) Materials, Optical Materials, Optoelectronic Properties, Opto-Electronic Devices, Nonlinear Optical (NLO) Applications, Nonlinear Optical (NLO) Materials, Second-Order Nonlinear Optical (NLO) Applications, Nonlinear Optics (NLO), Nonlinear Optical (NLO) Properties, Nonlinear Optical Applications, Optical Device Applications, Nonlinear Optics, Nonlinear Optics, Nonlinear Optical Materials, Optoelectronics, Nonlinear Optical Materials, Optoelectronic Devices, Nonlinear Optical (NLO) Applications, Nonlinear Optical (NLO) Properties, Nonlinear Optical (NLO) Materials, Nonlinear Optical (NLO) Effect, Non-Linear Optical (NLO) Materials, Nonlinear Optical (NLO) Devices, Red Optical Device, Nonlinear Optical (NLO) Material, Non-Linear Optics (NLO), Ferroelectric Properties, Nonlinear Optical (NLO) Properties, Non-Linear Optics, Optoelectronic Switching Devices, Optical Window Applications, Non-Linear Optical (NLO) Applications, Novel Optical Materials, Electronic Devices, Surface Photoelectronic Properties, Non-Linear Optical Materials, Optoelectronic Device, Optical Applications, Nonlinear Optical Application, Non-Linear Optical (NLO) Properties, Non-Linear Optical Properties, Nonlinear Optics (NLO) Materials, Nonlinear Optics Materials, Nonlinear Optic (NLO) Materials

11 MOF ChemUnity Knowledge Graph

11.1 Building the Knowledge Graph

Each extraction workflow results in a ".csv" file. To create a knowledge graph from this tabular data, we assign the different headings of these files to be Nodes or Relationships, based on our pre-defined schema. Each node and relationship type in the schema can store different pieces of information. For example, Experimental Property Nodes store a property name, and Has Property Relationships store Values, Units, Conditions, Summaries, and References. For our schema, most of the data is stored in relationships, connecting MOF nodes to various properties, applications, etc.

By describing our schema and the contents of our CSV files in depth to Chat-GPT4o, we obtained a set of cypher queries to import our data into Neo4j.[20] Imports can be done through the Neo4j python driver,[?] and the exact script used to generate the MOF ChemUnity knowledge graph is found in our code base. To run the script, Neo4j must be installed locally on your computer, and you must create a local graph "instance". Then, you simply add the Neo4j username, password, and URI as environment variables and run the python script.

11.2 Data Provenance

The design of the schema for MOF-ChemUnity ensures scalability to multiple sources of heterogeneous data which include experimental data from literature and computational data. However, the state-of-the-art and the accuracy of this data is always subject to changes due to realized inadequacies or sources of error. Therefore, the schema must model data provenance to facilitate updates to the knowledge graph, ensuring the data remains both current and accurate.

This is implemented using the “Has Source” relationship, which facilitates tracking of additional knowledge and potential updates by tracking citations to a reference DOI. Moreover, the “Has Property”, “Has Application” and “Has Synthesis” relationships for extracted information all include an attribute, “reference”, to allow traceability of sources. Consequently, handling contradictions can be facilitated by exploiting these references which are stored for all sources of extraction information.

For the computational labels and descriptors, on the other hand, the “Has Property” relationship includes a “based on” attribute which stores a reference to the source of CIF file used for the computation. Since CoRE MOF 2019 and QMOF structures can be vastly different the original deposited CIF files in CSD, it is important that each computational data point refers to which CIF was used in the computation. Moreover, storing this information also facilitates updates to the knowledge graph with new or updated datasets.

11.3 Query Agent Tool

To explore the knowledge graph locally with ease, we have built a "Query Agent" tool that turns natural language requests into cypher queries and returns tabular datasets. Some examples of natural language requests are: "All water stable MOFs with copper metal", or "All computational descriptors for Zinc MOFs".

This query tool is powered by GPT-4o, and the LLM system prompt contains two parts. Firstly, an instruction prompt defines the agent’s purpose, sets behavioral rules, and provides few-shot examples, as shown in Figure S.28. However, in order to generate Cypher queries that work with our specific graph, the agent must also be aware of the schema in detail. To achieve this, the LLM is given a JSON-style object in the system prompt that describes the graph schema, including node labels, relationship types, and key properties. This JSON object is not static, instead, it is generated dynamically at runtime by querying the live Neo4j database, allowing the query tool to remain compatible with future changes to the knowledge graph.

To construct this schema, the agent executes Cypher queries to extract key structural components:

- **Node schema extraction:**
  The method `_fetch_node_schema()` matches all nodes using `MATCH (n)`, extracts their labels and property keys using `labels(n)` and `keys(n)`, and then groups nodes by label (e.g., `Material:MOF`). It flattens and deduplicates the property keys across all nodes of the same label to build a mapping from node label to its sorted list of properties.

- **Relationship schema extraction:**
  The method `_fetch_rel_schema()` first retrieves all relationship types using `CALL db.relationshipTypes()`, and then, for each type, queries the graph to extract all unique property keys associated with that relationship. The result is a dictionary mapping each relationship type to its list of keys.

- **Supplementary fields:**
  Additional metadata is extracted to support retrieval tasks. `_fetch_property_names()` and `_fetch_application_names()` extract all distinct `name` values from `Property` and `Application` nodes, respectively.

The resulting schema is serialized into JSON and passed into the LLM's system prompt, enabling Cypher generation that is both schema-aware and resilient to structural changes in the underlying Neo4j database.

```
You are an expert Cypher query assistant for a Neo4j knowledge-graph of Metal-Organic Frameworks (MOFs).

You will receive TWO system messages:
  1 :  This instruction prompt.
  2 :  A JSON object that describes the live schema:
     {
       "nodes":         {label: [node_property_keys]},
       "relationships": {relType: [relationship_property_keys]},
       "propertyNames": [every valid p.name string],
       "applicationNames": [every valid Application.name string]
     }

Use that JSON to pick the correct labels, relationship types, and property keys.

RULES:
• Always return **one row per MOF**, using `m.refcode` as the ID.
• If multiple fields are requested, align them per-MOF with `OPTIONAL MATCH … WITH`.
• Use `p.name = "<exact string>"` only when matching `:Property` nodes.
  – Do **not** add extra labels like `:Computational` / `:Descriptor` unless the user explicitly asks.
• If the field lives on a relationship (see JSON relationship keys), reference it as `r.<field>` rather than `p.name`.
• Use `OPTIONAL MATCH` when data might be missing.
• Never add `LIMIT` unless the user asks.
• Output **only raw Cypher** — no markdown fences, no comments.
• If checking whether a property is “reported / available”, filter with `r.value IS NOT NULL`.

EXAMPLES:
● **Named property + synthesis field**
Q: List MOFs with `smiles_linker` and water stability
A:
MATCH (m:MOF)
OPTIONAL MATCH (m)-[r1:has_property]->(p1:Property)
WHERE p1.name = "water stability" AND r1.value IS NOT NULL
WITH m, r1.value AS water_stability
OPTIONAL MATCH (m)-[r2:has_synthesis]->(s:Synthesis)
RETURN m.refcode, r2.linker AS smiles_linker, water_stability
● **All descriptors (name / value pairs)**
Q: Show all descriptors and their values for every MOF
A:
MATCH (m:MOF)-[r:has_property]->(p:Property:Descriptor)
RETURN m.refcode, p.name AS descriptor_name, r.value AS descriptor_value
● **Applications**
Q: List MOFs with CO₂ storage applications and recommendations
A:
MATCH (m:MOF)-[r:has_application]->(a:Application)
WHERE toLower(a.name) CONTAINS "co2"
RETURN m.refcode, a.name AS application, r.recommendation
IMPORTANT → Never wrap the query in ``` back-tick fences and never emit extra prose.
```

Figure S. 28. Query Agent System Prompt. The instruction prompt for the query agent. The LLM sees this first, followed by the dynamically generated JSON object which gives schema specific details.

12 Graph-Enhanced RAG

12.1 Tasks

To demonstrate the future potential of Graph-Enhanced RAG for MOF research, we developed four benchmarking tasks: Property Prediction, Information Retrieval, Struture-Property Inference, and Material Recommendation. For all of these tasks, we show that injecting the LLM with few-shot context retrieved from the knowledge graph improves performance. Although this is one of the simplest forms of Graph-RAG, it acts as a proof-of-concept for future works.

12.1.1 Property Prediction

We compare the performance of Vanilla (non-augmented) LLMs with graph-enhanced LLMs in predicting the water stability of MOFs based off MOFid. We use a test-set of over 322 experimentally reported water stability labels to evaluate performance. The graph-enhanced workflow ufinds the 5-nearest MOFs with a reported water stability label in the train-set. The calculated similarity is based off of UMAP-embeddings of computational MOF descriptors. The stability label of the MOFs, as well as their distances to the test MOF, are given as few-shot context to the LLM, helping to improve performance from 33% accuracy to 56%.

12.1.2 Retrieval

Both LLMs are asked "Is ULMOF-5 water stable?". The graph-enhanced LLM can access the data in the MOF ChemUnity knowledge graph, and is able to successfully answer that the MOF is not water stable, as well as provide a source. When that source is inspected further, it can be observed that its water stability is reported with respect to the name "Compound 1". This shows the graph-enhanced workflow's ability to resolve co-references, as well as argument-mine the stability label from ambiguous text (inferring its dissolution in water and methanol to be "unstable"). Lastly, this workflow not only searches for a singular

MOF, but will search for all MOFs with the name "ULMOF-5". While not applicable here (there is only one MOF with this name in our database), it shows that the graph-enhanced workflow resolves duplicate entries in CSD, and will gather information from all crystal structures that are detected as duplicates.

12.1.3 Structure-Property Inference

Two structure-property inference questions are asked to the LLMs - "How are linker length and pore size connected to water stability?", and "How does metal node selection effect thermal stability". For these inference tasks, the query tool is used to collect a subset of relevant information from the graph and inject it as few-shot context. For the first question, 15 examples of MOF Pore Size, SMILES Linker, and Water Stability are collected. For the second, metal type and decomposition temperature are collected for 30 example MOFs.

12.1.4 Recommendation

Both LLMs are asked "What is a MOF that is similar to HKUST-1, is water stable, and could be used for carbon capture?". The graph-enhanced workflow uses the neighbour tool to find the most similar MOFs to HKUST-1 that are reported to be water stable, and uses the query tool to retrieve their CO2 uptakes. This information is injected as context to the LLM, and the MOF "PCN-46" is selected. As shown in Figure S.29, this MOF is much more similar to HKUST-1 than the vanilla LLM's suggestion, and has been recommended for Carbon Capture in its synthesis paper.[21]

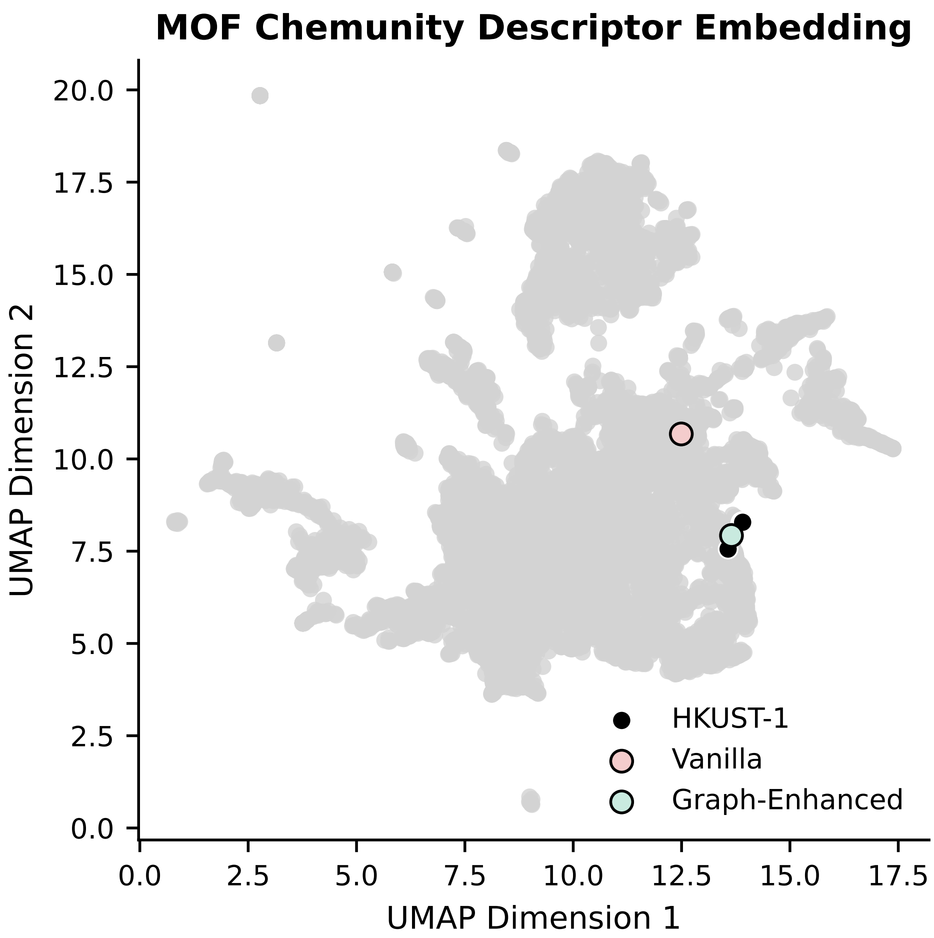

Figure S. 29. UMAP embedding space of MOF Descriptors The MOF that the Graph-Enhanced LLM recommends (PCN-46) is much more similar to HKUST-1 than the MOF the Vanilla LLM recommends (Mg-MOF-74).

12.2 Survey

To evaluate the quality and trustworthiness of the generated responses, a user study was conducted using an online survey tool. The survey covered three of the four benchmark tasks: Retrieval, Inference (Water Stability and Thermal Stability), and Recommendation. For each task, participants were shown a question along with two anonymized model responses (one from a vanilla LLM and one from a graph-enhanced LLM), in randomized order. Participants were asked to rate them in terms of perceived quality and trustworthiness on a scale of -5 to

+5. The complete survey details, including the exact wordings of the questions and model responses are shown in Table S.14

Table S. 14. Vanilla and graph-enhanced LLM responses shown to reviewers in the survey.

**Question**	**Vanilla LLM Response**	**Graph-Enhanced LLM Response**
Is ULMOF-5 water stable?	ULMOF-5's stability depends on metal-ligand bond strength, ligand hydrophobicity, and structural integrity, but no definitive answer can be given without more details.	ULMOF-5 is not water stable due to Li-based weak bonds and low surface area. Solubility in water and methanol confirms instability [doi:10.1021/cg100449z].
How does the selection of metal node affect the thermal stability of MOFs?	Metal node choice affects bond strength, oxidation state, coordination, cluster structure, and thermal conductivity. High-valent metals like Zr or Al increase stability.	Thermal stability is enhanced by strong metal-oxygen bonds and stable clusters. Examples include Er-MOF (480℃) [doi:10.1021/ic060568u] and Tm-MOF (560℃) [doi:10.1021/cg400531j].
How does pore volume and linker length affect the water stability of MOFs?	Larger pores and longer linkers increase water access and framework flexibility, reducing stability. Shorter linkers and small pores improve resistance to hydrolysis.	Larger pores and longer linkers reduce water stability. Stable examples include N,N'-piperazinebismethylenephosphonic acid MOF [doi:10.1021/ja804936z].
What is a relatively unstudied MOF that is similar to HKUST-1 and would be good for CO_2 capture?	MOFs like MOF-177, ZIF-8, MIL-101(Cr), UiO-66, and Mg-MOF-74 are similar and effective for CO_2 capture.	PCN-46 is structurally similar to HKUST-1 (distance: 0.43067) and has high CO_2 uptake (21.0 mmol/g at 30 bar) [doi:10.1039/c002767g].

Responses were collected using an online survey tool and standardized using Z-score normalization to account for differences in individual reviewer scoring tendencies. In total, we collected ratings and compiled results from nine reviewers: seven at the Professor, Postdoctoral, or Industry PhD level, and two current PhD students. Details on respondent demographics can be viewed in Figure S.30

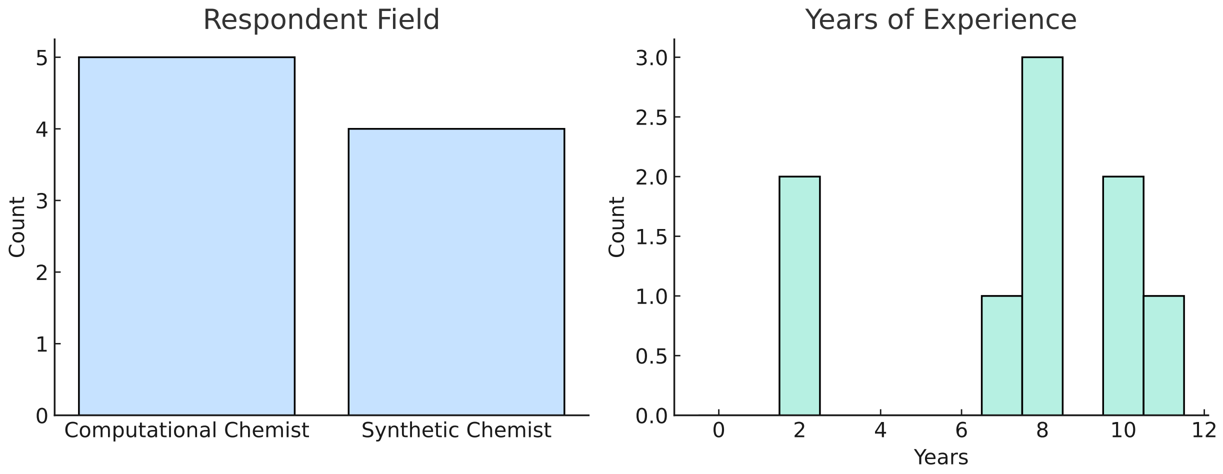

Figure S. 30. Survey Demographics. Summary of respondents from the response evaluation survey. **(Left)** Distribution of participants by research area, including computational and synthetic chemists. **(Right)** Histogram of self-reported years of experience.

13 Water stability modeling

From leveraging the extracted water stability labels, machine learning models were constructed in which it took the chemistry revised autocorrelations (RACs)[22,23] and geometric descriptors[24,25] of the MOFs as inputs to make classification predictions.

13.1 ML Features: RACs and geometric descriptors

There are two distinct inputs to the water stability machine learning model: the metal, ligand and functional group centered revision autocorrelation functions (RACs) and the geometric descriptors - which include largest included sphere, largest free sphere, largest included sphere along free path, accessible surface area (ASA), not accessible surface area (NASA), accessible volume (AV) and not accessible volume (NAV).

RACs, in this context, are defined as the product and differences on a graph of heuristic atomic properties.[22] It can be mathematically defined as:

$$P_d = \sum_i \sum_j P_i P_j \delta(d_{ij}, d)$$

$$P'_d = \sum_i \sum_j (P_i - P_j)\delta(d_{ij}, d)$$

Where P_d is the RAC for some property P at depth d (which is the number of bonds), P'_d is the analogous difference RAC, P_i is some property for some defined atom "i" and $\delta(...)$ is the Kronecker delta function.[9]

RACs[22] were calculated using the molSimplify 1.7.4 library[23] by getting these descriptors from the primitive cell of the input MOF structure. The parameters used for the computations are the depth (chosen as 3) and the maximum number of allowed atoms (6000). The geometric descriptors were computed through utilizing Zeo++ v0.3,[24,25] with a probe radius of 1.4 Åfor surface area and volume calculations. A Monte Carlo sampling procedure is used for the surface area and probe-occupiable volume computations, with 10000 samples chosen.

13.2 Machine learning to predict water stability of MOF

The model utilized is an XGBoost model from the "xgboost" open-source package. Before training the model, the labels should be in a discretized, binary space. The stability labels initially extracted were either given as "stable" or "unstable" strings. This space can be discretized such that:

$$S = \begin{cases} 0, & \text{if unstable} \\ 1, & \text{if stable} \end{cases}$$

13.3 Baseline model

When referring to the "baseline" model, this is a model in which no hyperparameter tuning, sampling method or uncertainty assessment is utilized on it. From the water stability dataset, an 80/20 train/test split was taken, and an XGBoost classifier model was trained with its default parameters. It can be seen that, while the metrics utilized indicate that the model

performs well, when looking at the confusion matrix (Figure 31), the model performs poorly when attempting to predict unstable labels (hence the low balanced accuracy of 70%). We believe this may be due to the dataset class imbalance or unstable labels being generally more difficult to predict.

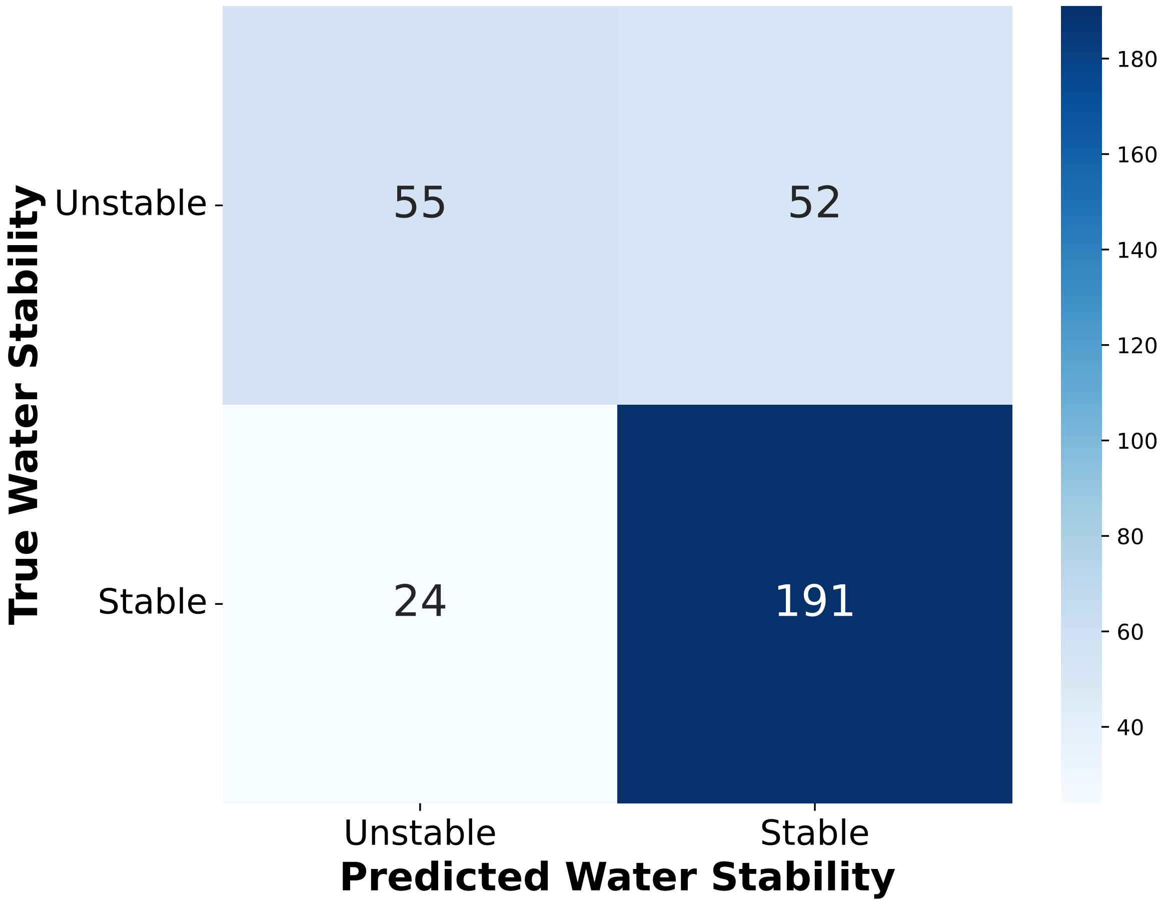

Figure S. 31. Confusion matrix for baseline model An XGBoost classifier was trained on a random 80/20 split (random seed = 1 arbitrarily chosen), with its default hyperparameters from the xgboost package. For this particular seed, F1 = 0.83, accuracy = 76%, balanced accuracy = 70%, recall = 0.89, precision = 0.79, ROC-AUC = 0.77, unstable accuracy = 51%.

13.4 Hyperparameter optimization

Due to the class imbalance, hyperparameter tuning has proven to be instrumental in making our model more "pessimistic" (i.e. inclined to predict unstable more often). For applications

purposes, a pessimistic model is usually preferred as when screening for MOFs, experimentalists require water stable MOFs, and a model that predicts stable more than unstable would incorrectly provide experimentalists with unstable MOFs - thus making it inefficient and slow down material discovery. As a result, we are interested in:

1. Making a model that scores well in terms of unstable accuracy and precision. The unstable accuracy can be defined as:

$$U_S = \frac{TN}{TN + FP}$$

   When referring to negative and positive, negative is considered unstable, and positive is considered stable.

2. Of course, we want our model to still be balanced (and not always predict unstable, as that would mean there will be an opportunity cost), so recall should be considered too.

During hyperparameter optimization, we decided to use a BayesSearchCV (CV = 5) approach, in which Bayesian Optimization is used to explore a space of hyperparameters to maximize the following custom function:

$$f = \alpha_1 U_s + \alpha_2 P + \alpha_3 R$$

Where U_s, P and R are unstable accuracy, precision and recall respectively. The coefficients for unstable accuracy, precision and recall were chosen as 0.90, 0.20 and 0.94 respectively. The search space explored (while setting maximum delta step as 1.0) was:

1. Learning rate, from 0.0001 to 1.0; optimal learning rate has been found to be 0.72.

2. maximum depth, from 1 to 6 (integers); optimal maximum depth has been found to be 5.

3. "scale pos weight", from 0.0001 to 10; optimal scale pos weight has been found to be 0.13.

Figure 32 shows the updated confusion matrix, showing a much improved prediction of unstable accuracy compared to the baseline model.

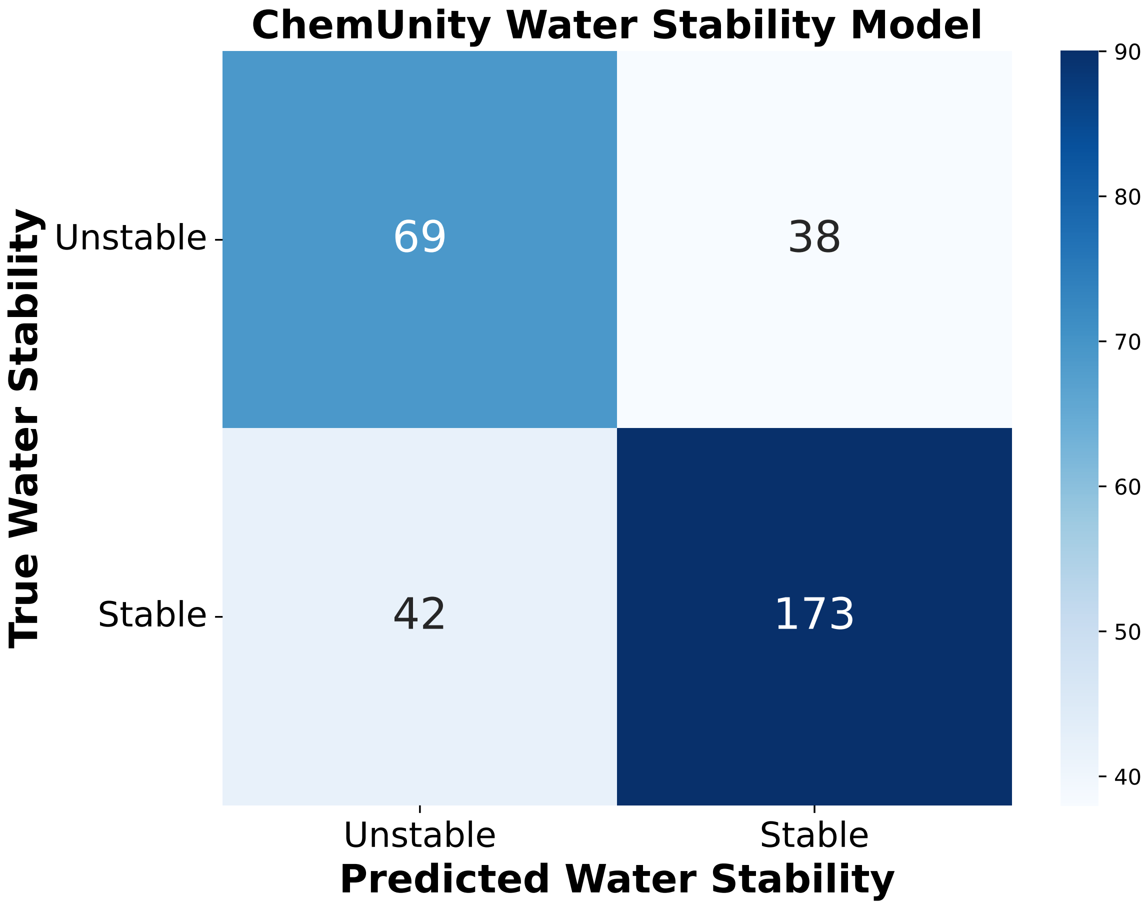

Figure S. 32. Confusion matrix for hyperparameter optimized model From using the previously described tuned hyperparameters in the new XGBoost classifier model, the water stability predictions have been shown to be significantly more balanced in comparison to Figure 31 (especially in terms of unstable accuracy). The metric are F1-score = 0.81, accuracy = 75.2%, balanced accuracy = 72.5%, recall = 0.80, precision = 0.82.

K-Fold cross validation was done on both the baseline and hyperparameter-tuned models (k = 10). It can be seen that between the baseline and hyperparameter tuned model, there is a sharp drop in the recall score (0.87 to 0.79). However, our objective was to make the

model more pessimistic and improve property predictions on unstable MOFs while retaining a respectable performance on predicting stable MOFs, which was achieved from looking at the balanced accuracy (65% to 70%), precision (0.76 to 0.80) and unstable accuracy (44% to 61%).

Table S. 15. Performance comparison between the baseline and hyperparameter-optimized models. Mean values are presented with their respective standard deviations.

**Metric**	**Baseline**	**Hyperparameter Optimized**
F1-Score	0.81 ± 0.03	0.79 ± 0.03
Accuracy (%)	73 ± 3.42	73 ± 3.45
Balanced Accuracy (%)	65 ± 3.84	70 ± 3.63
Unstable Accuracy (%)	44 ± 6.60	61 ± 6.98
Precision	0.76 ± 0.03	0.80 ± 0.05
Recall	0.87 ± 0.04	0.79 ± 0.04
ROC-AUC	0.75 ± 0.05	0.74 ± 0.04

13.5 Impact of probability threshold

The logic used in the XGBoost classifier is, by default:

$$y_{\text{pred}} = \begin{cases} 1, & \text{if } P(y|x) \geq 0.5 \\ 0, & \text{otherwise} \end{cases}$$

In this case, the threshold probability is 0.5. This can be played with to find some optimal probability threshold based on our previously stated objective. To do this, an 80/10/10 train/val/test split was done. Next, the threshold was varied from 0.1 to 0.95, and the model was trained on the train set and, from using the new threshold, the binary predictions were made on the validation set. The stable and unstable accuracies on the validation set with respect to the threshold were collected and plotted (Figure 33). From inspection of the profiles, a threshold of 0.63 was chosen.

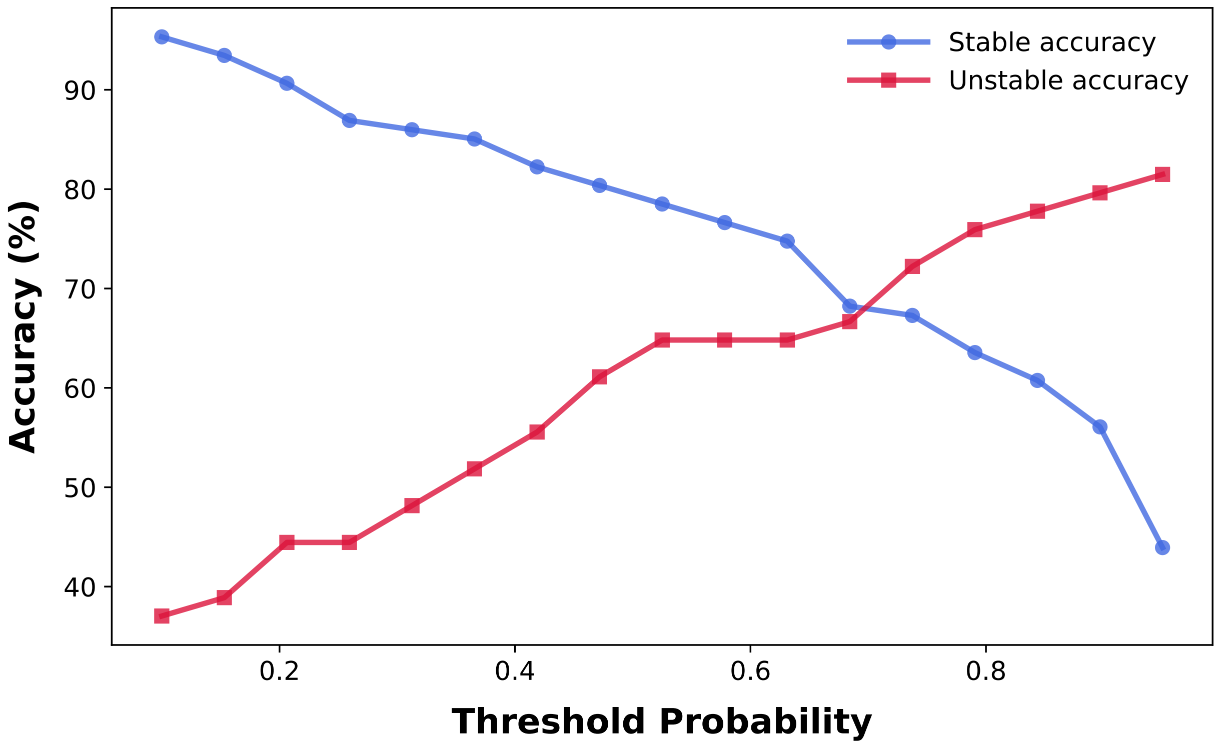

Figure S. 33. Stable and unstable accuracies on validation set for threshold assessment From doing an 80/10/10 train/validation/test split, the model was trained on the train set and evaluated on the validation set with varying probability thresholds.

From using the selected threshold, the same evaluation was done on the test set. Figure 34 shows the confusion matrix on the test set with the new threshold, showcasing an improvement in the unstable property prediction, while retaining balance when predicting the stable MOFs.

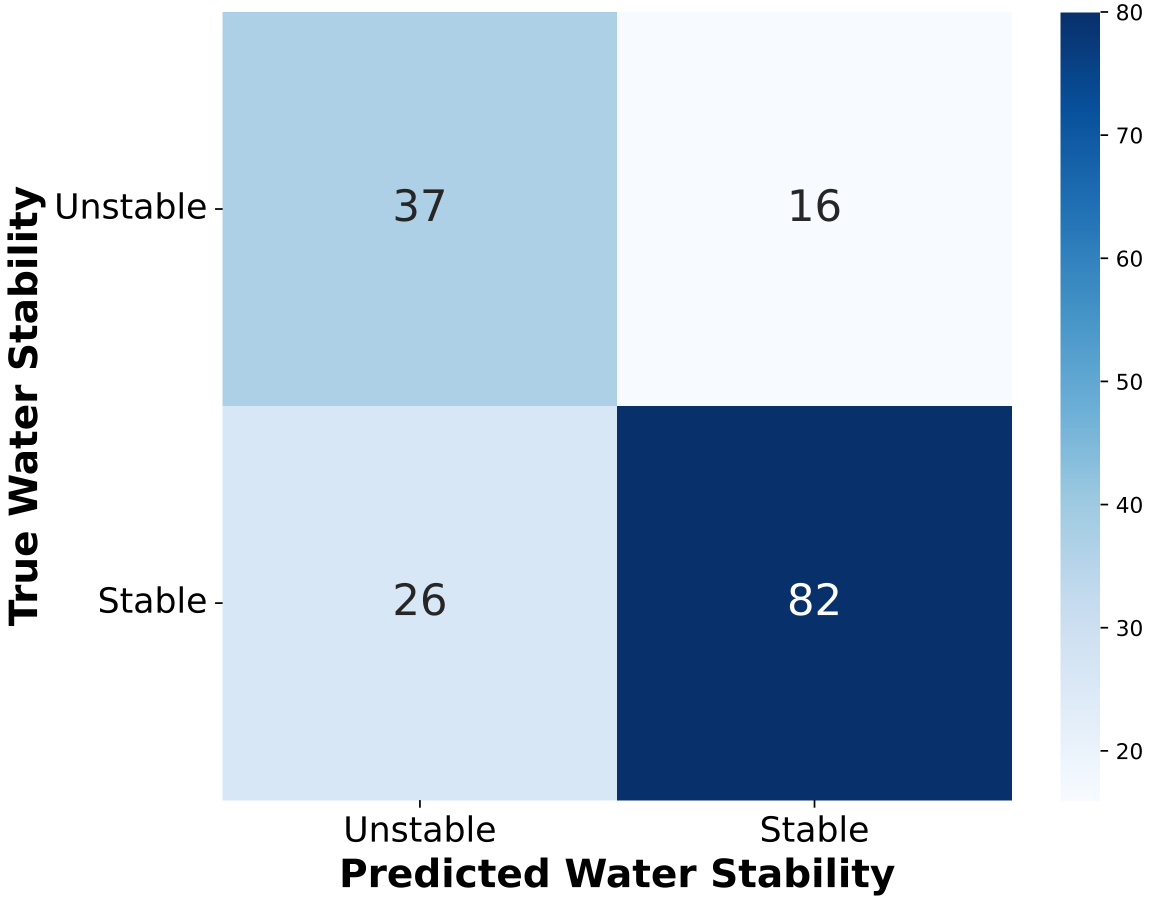

Figure S. 34. Confusion matrix for chosen probability threshold From using a selected probabilistic threshold of 0.63, the model is shown to be more accurate in predicting the unstable MOFs, while retaining balance towards stable MOFs. Metrics are F1 = 0.80, accuracy = 73.9%, balanced accuracy = 72.8%, recall = 0.76, precision = 0.84, ROC-AUC = 0.79, unstable accuracy = 70%.

13.6 Uncertainty assessment

An uncertainty assessment was done to remove data points that the model may not be sure of. This was achieved by:

1. Doing an 80/20 train/test split of the data;
2. Performing bootstrapping on train set (splitting 80% of train) to retrieve 50 different training subsets;

3. Training 50 different XGBoost classifiers on those 50 sets, creating an ensemble of models;

4. Evaluate this ensemble on the test set to get the mean probabilities and their corresponding standard deviations for each data point;

5. Remove data point "i" if the following condition is met: $\sigma_i \geq \sigma_{\text{threshold}}$

A standard deviation of threshold is chosen as it increases the scores of the model while retaining a reasonable amount of test data points (30% of the test set filtered out). The calibrated model is compared to the baseline model (no data points removed) and a model trained and evaluated on WS24s[9] (Figure S35). The model has, overall, an improved performance - particularly in the unstable accuracy and precision.

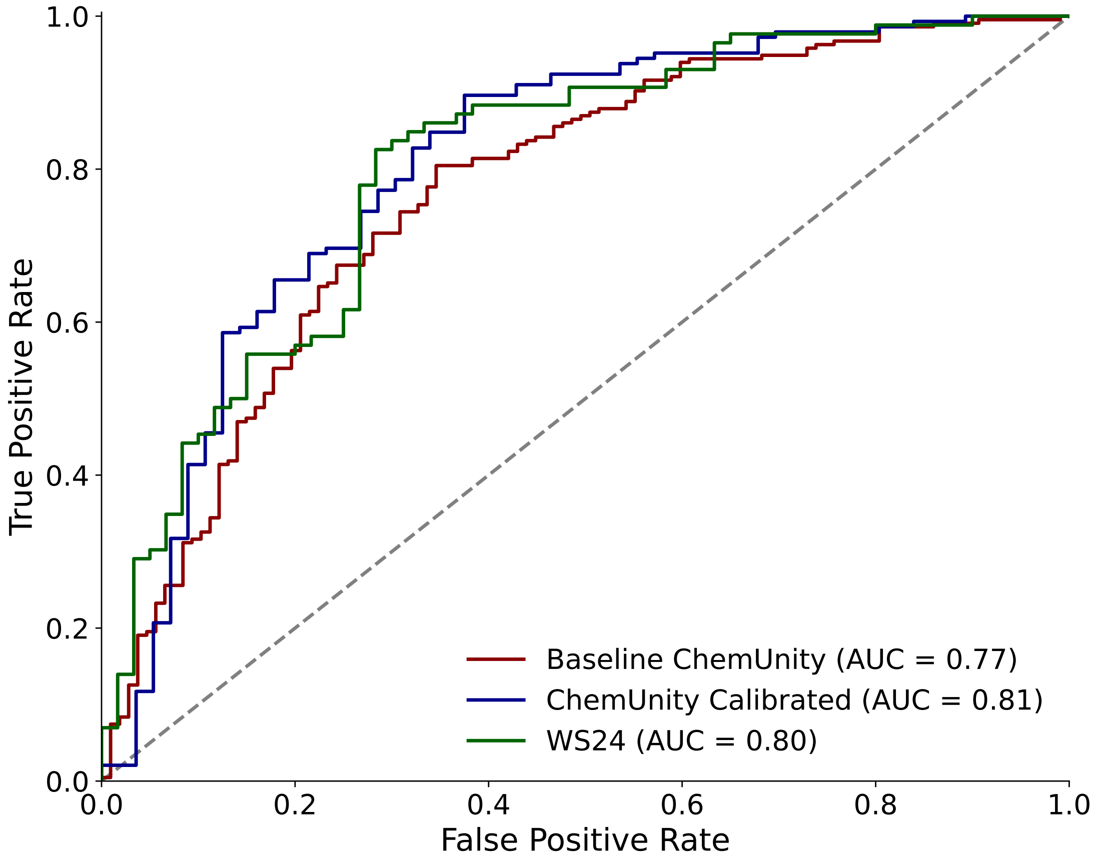

Figure S. 35. ROC-AUC curves comparing the model that is uncertainty calibrated (blue) with the baseline (red) and WS24s (another water stability dataset) From this, when comparing the calibrated model to the baseline, there is a clear improvement in ROC-AUC, and the score is also comparable to a model trained and evaluated on WS24s. For uncertainty calibrated model, metrics: F1 = 0.86, accuracy = 80.1%, balanced accuracy = 75%, recall = 0.87, precision = 0.86, unstable accuracy = 63%.

13.7 Feature importance in water stability model

The model feature importances in Figure S. 36 indicate that the model is primarily reliant on metal-centered, metal-linker coordinating atom and functional group-based descriptors. In general, the most "important" atomistic properties that appear with high importance include covalent radii (S), electronegativity (chi) and nuclear charge (Z), but there are also

mentions of the connectivity (T). These observations are commented on in Section 13.9 (SHAP analysis).

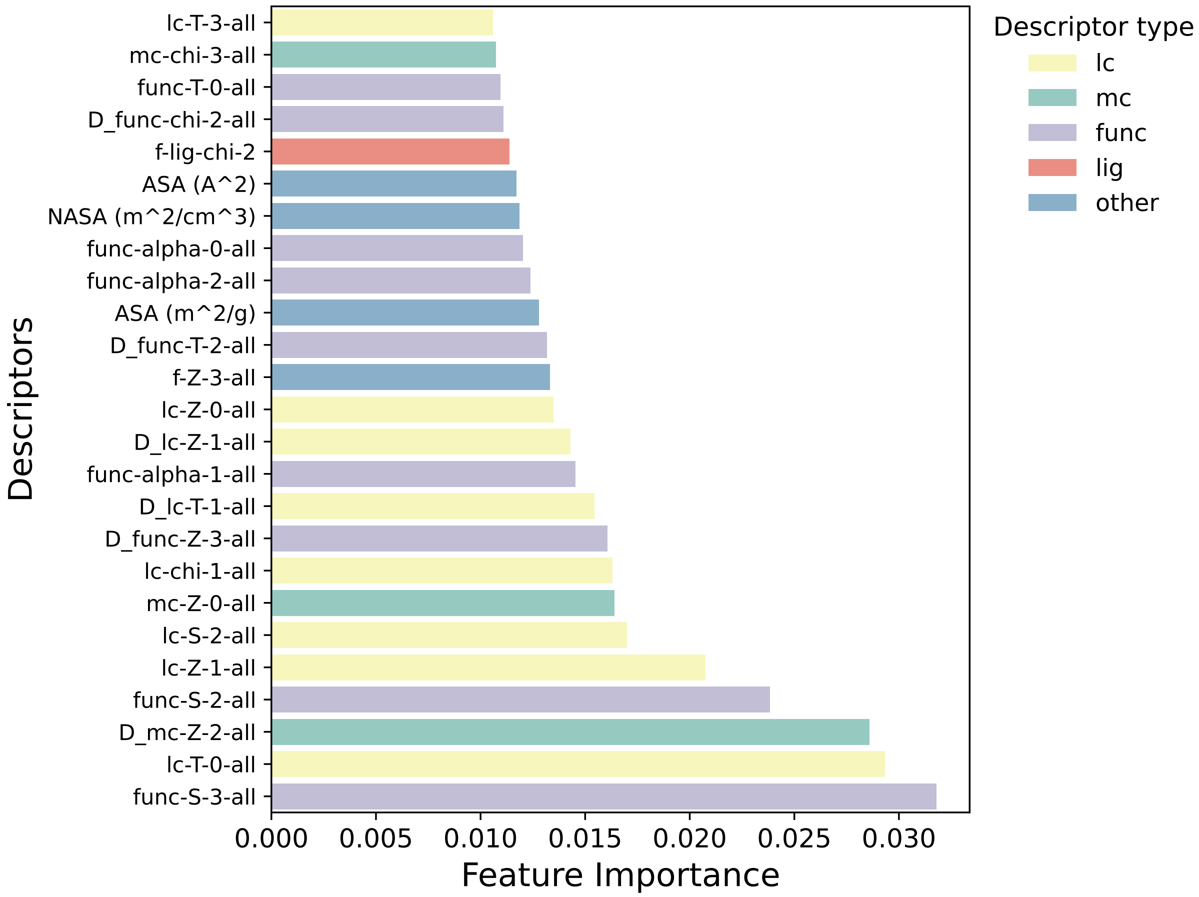

Figure S. 36. Feature importances for machine learning model in predicting stability) From this, it is possible to see which features contribute to machine learning model while being split into categories per descriptor type. The metal-centered, functional group and linker-coordinating atom descriptors are shown to be the most important descriptors, validating MOF literature in water stability.[26] lc: linker-connecting atoms (yellow). mc: metal-centered (green). func: functional groups (purple). lig: full ligand (red). other: either f- (descriptors describing full MOF unit cell) or geometric descriptors (blue).

13.8 SHAP analysis on model

Figure S. 37 shows a SHAP analysis done on the machine learning model, with the top 15 features (selected from the top SHAP values) displayed. There are a couple of interesting

observations of note. It can be seen that with a more packed framework, the MOF becomes more stable, which is a trend that has not been noted before to the author's knowledge. One explanation for this can be explained through the slight inverse correlation between the crystal density and the largest cavity diameter (LCD); with a higher density, it generally leads to a lower LCD (and thus, a more water stable framework). Some more interesting results can be seen with the descriptors in which the electronegativity (chi), nuclear charge (Z) and polarizability (alpha) are used as the atomistic properties. For instance, D-lc-chi-1-all is the electronegativity of the scope of atoms that are within 1 bond away from the linker-connecting atom (including the coordinating atom). The SHAP analysis indicates that an increase in this leads to more stability in general, which aligns with the intuition that a higher electronegativity of the coordination atom leads to an increase in the metal node-linker bond strength, thus improving stability. However, there are some results that are considered surprising. For instance, from using the definition of RACs (Section 13.1), D-lc-alpha-3-all is the polarizability of the scope of atoms that are within 3 bonds away from the linker-connecting atom (difference). The SHAP analysis indicates that an increase in polarizability of these atoms leads to more water stability. This is a surprising result, as dipole-dipole interactions between the polarized atoms and water molecules may lead to the framework losing structural integrity. A geometric property that is shown to show a surprising result in the SHAP result is the largest included sphere diameter (D_i), as it is implied that a larger D_i leads to a more water stable MOF. However, as there is an inverse correlation between the linker length and water stability, this result is considered quite surprising. Overall, the SHAP analysis provided an intuition on which atomistic properties and which parts of the MOF framework are considered interesting and important to look into for water stability assessment in MOFs.

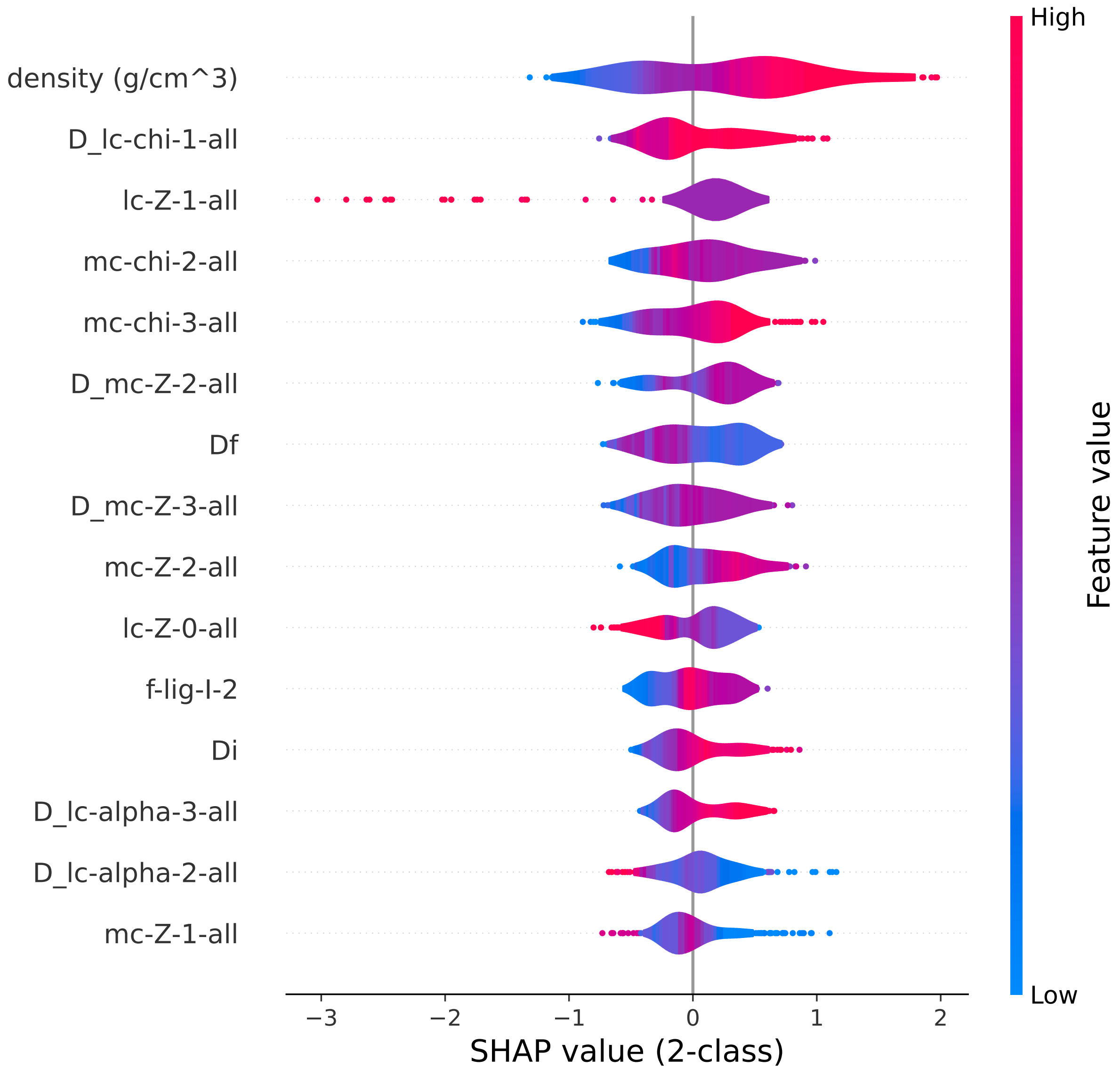

Figure S. 37. SHAP analysis on the hyperparameter-tuned model across the top 15 features (selected from top SHAP values) The top 15 features are listed from top to bottom, with increasing feature values denoted by the colour bar. As you go from left to right, the stability of the MOF increases for that particular feature.

14 Water stable MOFs for carbon capture

14.1 MOF curation workflow

Our selection of MOFs were done through a set of curation steps that we summarize in Figure S. 38, with the goal of identifying water stable MOFs with high CO_2 adsorption. First, the entire ChemUnity database is screened by using the pretrained machine learning model to predict water stability, and all MOFs flagged as unstable are filtered out. Then, using literature-informed decision-making, MOFs that are classified as promising for carbon capture are filtered by using pre-calculated CO_2 uptake values (at 0.15 bar).[22] MOFs that are calculated to have an uptake greater than or equal to 2 mol/kg are flagged as promising, with the rest filtered out.[27,28] The selected MOFs at this stage go through a couple of checks and further refinements. The main motivation for this is that computational databases, such as CoRE MOF-2019, may have erroneous structures.[14] Moreover, the computational properties by Moosavi et al.[22] were performed over original structures from CoRE MOF-2019 without DFT optimization as well as using point charges from EQeq method. Here, we aim to improve on these. For starters, as the calculations for CO_2 uptake are done on computationally ready (CR) MOF structures, there is a risk of part of the structure being removed during the process of solvent removal. A manual check is done by comparing the CR structure with its CSD counterpart, and if the structures are evidently the same (apart from the presence of clear bounded/unbounded solvents), the structure is kept, else it is taken out. Furthermore, interpenetrability is a concern in CO_2 uptake applications, and MOFs that contained interpenetrability were removed from our subset. A MOF validation check is done using MOFChecker,[29] with a further oxidation state check done by utilizing MOSAEC.[14] These checks remove structures with wrong charges as a result of removing solvents that are part of the structure.

The resulting set is then optimized using density functional theory (DFT) and DFT-derived partial charges (DDEC) were computed for this set. Finally, we compute the CO_2

Henry coefficient using Widom insertion on the optimized structure using the DDEC charges (Details of simulation can be found on section 14.2).

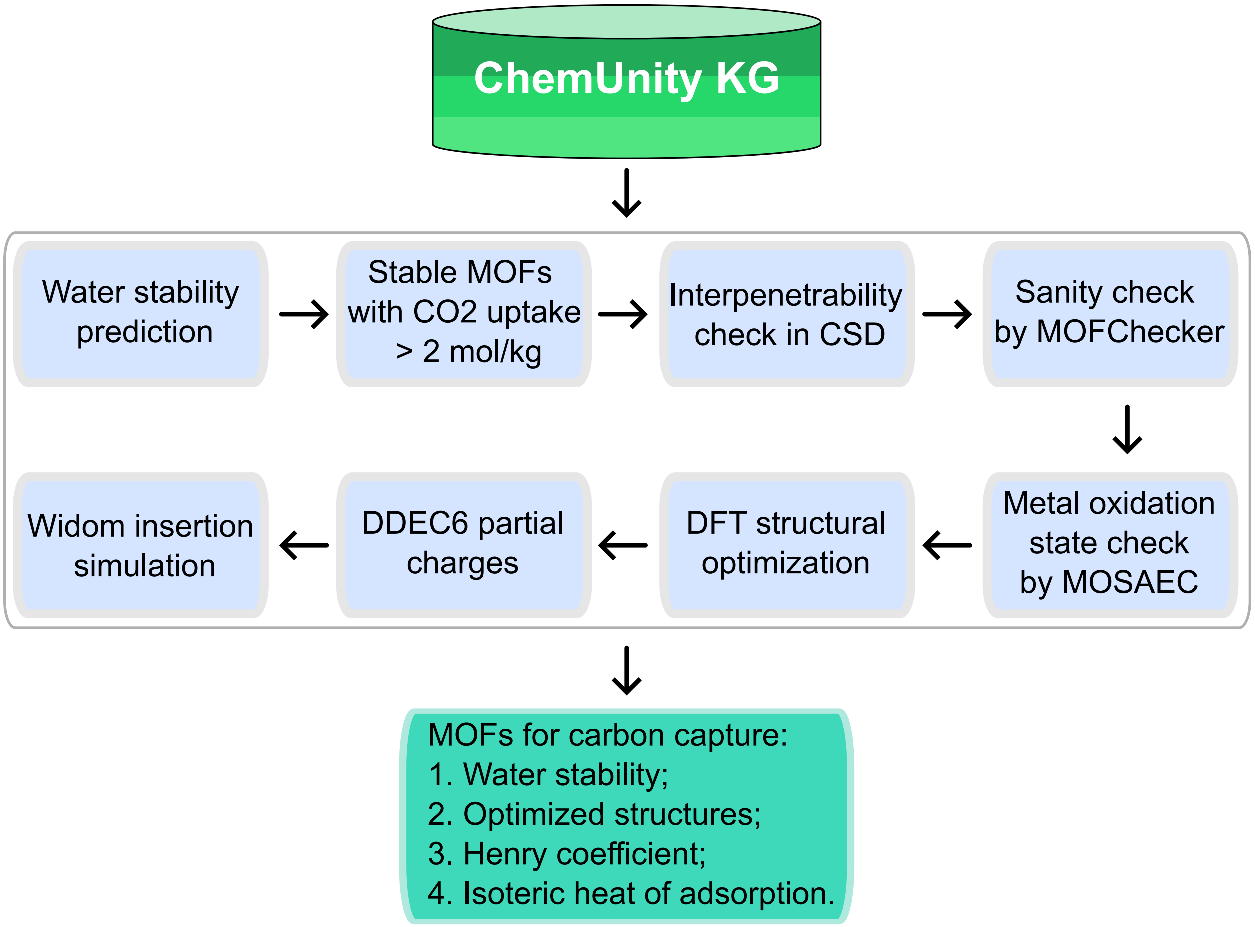

Figure S. 38. MOF curation workflow. The workflow of selecting MOFs from ChemUnity KG with structures are water stable and are good for carbon capture application. It includes water stability prediction, initial check literature CO_2 uptake and structural checks, and the molecular simulations of carbon capture properties.

Figure S. 39 shows the resulting structures from this curation workflow. While some structures show volume change in during the DFT optimization, the results show that the majority of structures retain their porous structure and there isn't a collapse in pore volume during the structure optimization. From this curation workflow, we have successfully identified a set of MOFs that are predicted to be stable, do not have their pore volume collapsed, have a high Henry coefficient for CO_2 (within the accuracy of DFT-derived partial charges and UFF force field) and high Q_{st}, such as ZEDQEG, NEYZAU, and XUMRUU.[30–32]

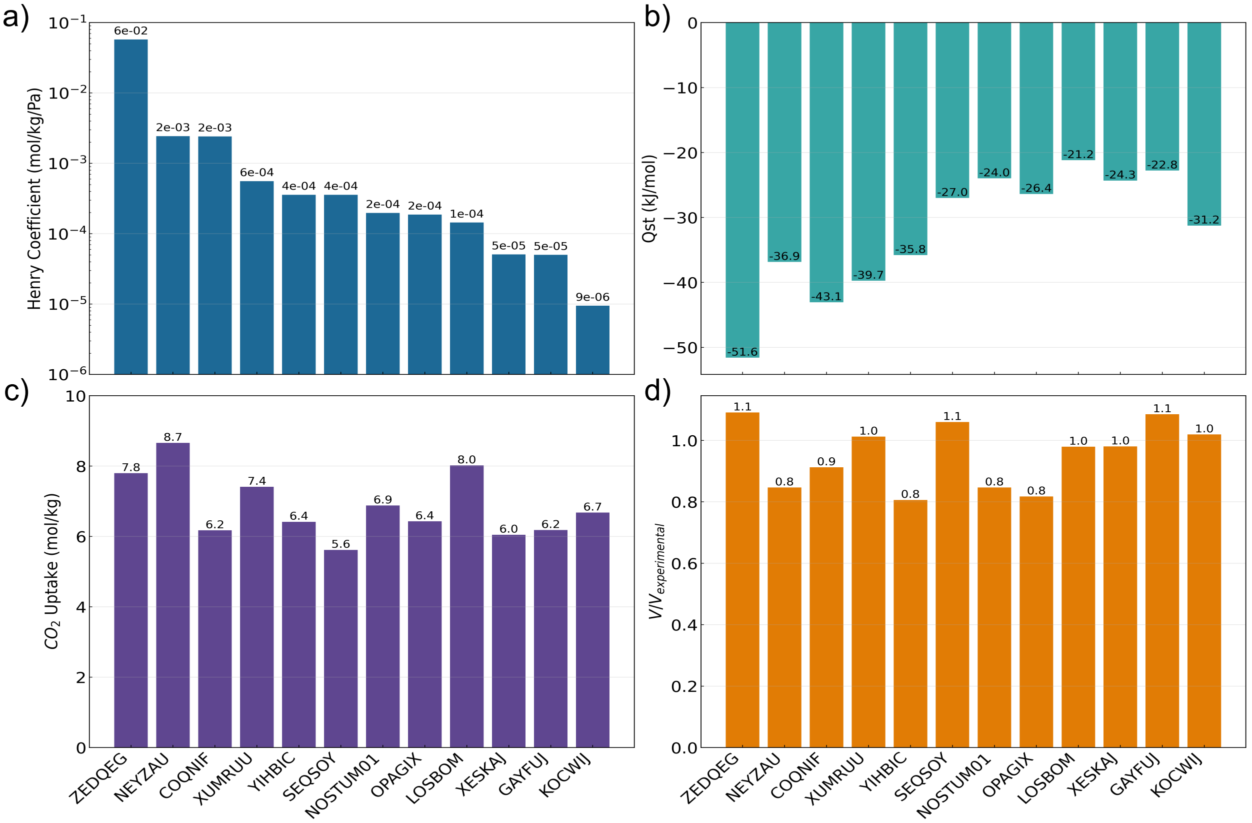

Figure S. 39. Molecular simulation results of predicted water stable MOFs. (**a**) Henry coefficient and (**b**) isoteric heat of adsorption from Widom insertion simulations at 298 K. (**c**) The CO_2 uptake from the work of Moosavi et al.[22] (**d**) The normalized volumes of MOF structures after DFT optimization to before optimization. The $V_{experimental}$ means the structural volume without optimization.

14.2 DFT and molecular simulation details

DFT and molecular simulations of these structures were performed with automated workflows and through the Automated Interactive Infrastructure and Database for Computational Science, AiiDA.[33] The structural optimization, the partial charges, and the Widom insertion simulations were executed through the calculation functions and work chains of the *aiida-lsmo* plugin.[34,35]

The work chain of structural optimization and DDEC6 partial charges[36] was *Cp2k-MultistageDdec* with executing the quickstep code of the CP2K version 2023.1.[37] The experimental structures were optimized with geometry and cell. DFT simulations were performed

with the Perdew–Burke–Ernzerhof (PBE) functionals[38] with dispersion corrections of DFT-D3(BJ).[39] The DZVP-MOLOPT-SR basis set, GTH pseudopotential,[40] and Gaussian plane wave were used in CP2K. The cutoff energy of the plane wave, the energy convergence and maximum force were 800 Ry, 1.0E−8 Ry and 0.00015 $bohr^{-1} \times$ hartree, respectively. The DDEC6 partial charges were derived from the electron density distribution of fully optimized structures from CP2K using Chargemol version 3.5.

The Widom insertions were computed with RASPA2 package with the workchain of *SinglecompWidom*. All these molecular simulations were simulated with the optimized structures from above DFT. The Widom insertions were performed at zoom temperature of 298 K. The force field parameters for MOF frameworks and CO_2 molecules were from UFF[41] and TraPPE,[42] respectively. The interactions of host-guest and guest-guest were modeled Lennard-Jones potential truncated and shifted at 12.8 Å and the Coulombic electrostatic interactions computed by Ewald summation. The pore geometry was performed from using Zeo++.[24,43] The Henry coefficient and the isoteric heat of adsorption of all structures were simulated with 100000 cycles. All other parameters were set to the default settings in the related work chains.

References

(1) Chung, Y. G.; Haldoupis, E.; Bucior, B. J.; Haranczyk, M.; Lee, S.; Zhang, H.; Vogiatzis, K. D.; Milisavljevic, M.; Ling, S.; Camp, J. S.; others Advances, Updates, and Analytics for the Computation-Ready, Experimental Metal–Organic Framework Database: CoRE MOF 2019. *Journal of Chemical & Engineering Data* **2019**, *64*, 5985–5998.

(2) Rosen, A. S.; Iyer, S. M.; Ray, D.; Yao, Z.; Aspuru-Guzik, A.; Gagliardi, L.; Notestein, J. M.; Snurr, R. Q. Machine learning the quantum-chemical properties of metal–organic frameworks for accelerated materials discovery. *Matter* **2021**, *4*, 1578–1597.

(3) Rosen, A. S.; Fung, V.; Huck, P.; O'Donnell, C. T.; Horton, M. K.; Truhlar, D. G.; Persson, K. A.; Notestein, J. M.; Snurr, R. Q. High-throughput predictions of metal–organic framework electronic properties: theoretical challenges, graph neural networks, and data exploration. *npj Computational Materials* **2022**, *8*, 1–10.

(4) Moghadam, P. Z.; Li, A.; Wiggin, S. B.; Tao, A.; Maloney, A. G.; Wood, P. A.; Ward, S. C.; Fairen-Jimenez, D. Development of a Cambridge Structural Database subset: a collection of metal–organic frameworks for past, present, and future. *Chemistry of Materials* **2017**, *29*, 2618–2625.

(5) Lewis, P.; Perez, E.; Piktus, A.; Petroni, F.; Karpukhin, V.; Goyal, N.; Küttler, H.; Lewis, M.; Yih, W.-t.; Rocktäschel, T.; Riedel, S.; Kiela, D. Retrieval-augmented generation for knowledge-intensive nlp tasks. *Advances in Neural Information Processing Systems* **2020**, *33*, 9459–9474.

(6) Ong, S. P.; Richards, W. D.; Jain, A.; Hautier, G.; Kocher, M.; Cholia, S.; Gunter, D.; Chevrier, V. L.; Persson, K. A.; Ceder, G. Python Materials Genomics (pymatgen):

A robust, open-source python library for materials analysis. *Computational Materials Science* **2013**, *68*, 314–319.

(7) Ansari, M.; Moosavi, S. M. Agent-based learning of materials datasets from the scientific literature. *Digital Discovery* **2024**, *3*, 2607–2617.

(8) Cohen, A. *TheFuzz*: Fuzzy String Matching for Python. Version 0.22.1; GitHub: San Francisco, California, USA, 2024.

(9) Terrones, G. G.; Huang, S.-P.; Rivera, M. P.; Yue, S.; Hernandez, A.; Kulik, H. J. Metal–organic framework stability in water and harsh environments from data-driven models trained on the diverse WS24 data set. *Journal of the American Chemical Society* **2024**, *146*, 20333–20348.

(10) Park, H.; Kang, Y.; Choe, W.; Kim, J. Mining insights on metal–organic framework synthesis from scientific literature texts. *Journal of Chemical Information and Modeling* **2022**, *62*, 1190–1198.

(11) Kang, Y.; Lee, W.; Bae, T.; Han, S.; Jang, H.; Kim, J. Harnessing Large Language Models to Collect and Analyze Metal–Organic Framework Property Data Set. *Journal of the American Chemical Society* **2025**, *147*, 3943–3958.

(12) OpenAI text-embedding-ada-002 Text Embedding Model. 2022; `https://openai.com/research/new-and-improved-embedding-model`, Initial release 15 Dec 2022; model accessed 15 Nov 2024.

(13) Pokrass, M.; Colby, C.; Guan, M.; Sanders, T.; Zhang, B. Introducing Structured Output In The API. 2024.

(14) White, A. J.; Gibaldi, M.; Burner, J.; Mayo, R. A.; Woo, T. K. High Structural Error Rates in "Computation-Ready" MOF Databases Discovered by Checking Metal Oxidation States. *Journal of the American Chemical Society* **2025**, *147*, 17579–17583.

(15) Computation-Ready, Experimental Metal-Organic Framework (CoRE MOF) 2019 Dataset. doi: `10.5281/zenodo.3370144`, Accessed: 2019-06.

(16) Rosen, A. S.; Iyer, S. M.; Ray, D.; Yao, Z.; Aspuru-Guzik, A.; Gagliardi, L.; Notestein, J. M.; Snurr, R. Q. Machine learning the quantum-chemical properties of metal–organic frameworks for accelerated materials discovery. *Matter* **2021**, *4*, 1578–1597.

(17) Paruchuri, V. *Marker*. Version 1.9.3; GitHub: San Francisco, California, USA, 2020.

(18) Lopez, P. Pub2TEI: A Service for Converting and Enhancing Heterogeneous Publisher XML Formats into TEI. 2015; `https://github.com/kermitt2/Pub2TEI`, Accessed: 2025-12-1.

(19) Bucior, B. J.; Rosen, A. S.; Haranczyk, M.; Yao, Z.; Ziebel, M. E.; Farha, O. K.; Hupp, J. T.; Siepmann, J. I.; Aspuru-Guzik, A.; Snurr, R. Q. Identification Schemes for Metal–Organic Frameworks To Enable Rapid Search and Cheminformatics Analysis. *Crystal Growth & Design* **2019**, *19*, 6682–6697.

(20) *Neo4j*. version 4.4.39; Neo4j, Inc: San Mateo, California, USA, 2024.

(21) Zhao, D.; Yuan, D.; Yakovenko, A.; Zhou, H.-C. A NbO-type metal–organic framework derived from a polyyne-coupled di-isophthalate linker formed in situ. *Chemical communications* **2010**, *46*, 4196–4198.

(22) Moosavi, S. M.; Nandy, A.; Jablonka, K. M.; Ongari, D.; Janet, J. P.; Boyd, P. G.; Lee, Y.; Smit, B.; Kulik, H. J. Understanding the diversity of the metal-organic framework ecosystem. *Nature communications* **2020**, *11*, 1–10.

(23) Ioannidis, E. I.; Gani, T. Z.; Kulik, H. J. molSimplify: A toolkit for automating discovery in inorganic chemistry. *Journal of computational chemistry* **2016**, *37*, 2106–2117.

(24) Willems, T. F.; Rycroft, C. H.; Kazi, M.; Meza, J. C.; Haranczyk, M. Algorithms and tools for high-throughput geometry-based analysis of crystalline porous materials. *Microporous and Mesoporous Materials* **2012**, *149*, 134–141.

(25) Martin, R. L.; Smit, B.; Haranczyk, M. Addressing challenges of identifying geometrically diverse sets of crystalline porous materials. *Journal of chemical information and modeling* **2012**, *52*, 308–318.

(26) Burtch, N. C.; Jasuja, H.; Walton, K. S. Water stability and adsorption in metal–organic frameworks. *Chemical reviews* **2014**, *114*, 10575–10612.

(27) Mahajan, S.; Lahtinen, M. Recent progress in metal-organic frameworks (MOFs) for CO2 capture at different pressures. *Journal of Environmental Chemical Engineering* **2022**, *10*, 108930.

(28) Khan, S. T.; Moosavi, S. M. Connecting metal-organic framework synthesis to applications using multimodal machine learning. *Nature Communications* **2025**, *16*, 5642.

(29) Jin, X.; Jablonka, K. M.; Moubarak, E.; Li, Y.; Smit, B. MOFChecker: a package for validating and correcting metal–organic framework (MOF) structures. *Digital Discovery* **2025**,

(30) Zhu, Z.-B.; Wan, W.; Deng, Z.-P.; Ge, Z.-Y.; Huo, L.-H.; Zhao, H.; Gao, S. Structure modulations in luminescent alkaline earth metal-sulfonate complexes constructed from dihydroxyl-1, 5-benzenedisulfonic acid: Influences of metal cations, coordination modes and pH value. *CrystEngComm* **2012**, *14*, 6675–6688.

(31) Gong, Y.; Hao, Z.; Li, J.; Wu, T.; Lin, J. Mg (II)-induced second-harmonic generation based on bis-monodentate coordination mode of thiobarbiturate. *Dalton Transactions* **2013**, *42*, 6489–6494.

(32) Wang, H.; Yi, F.-Y.; Dang, S.; Tian, W.-G.; Sun, Z.-M. Rational assembly of Co/Cd-MOFs featuring topological variation. *Crystal growth & design* **2014**, *14*, 147–156.

(33) Pizzi, G.; Cepellotti, A.; Sabatini, R.; Marzari, N.; Kozinsky, B. AiiDA: automated interactive infrastructure and database for computational science. *Computational Materials Science* **2016**, *111*, 218–230.

(34) Ongari, D.; Yakutovich, A. V.; Talirz, L.; Smit, B. Building a consistent and reproducible database for adsorption evaluation in covalent–organic frameworks. *ACS central science* **2019**, *5*, 1663–1675.

(35) LSMO, T. team, A. An aiida workflows for the LSMO laboratory at EPFL (2023). 2023.

(36) Manz, T. A.; Limas, N. G. Introducing DDEC6 atomic population analysis: part 1. Charge partitioning theory and methodology. *RSC advances* **2016**, *6*, 47771–47801.

(37) VandeVondele, J.; Krack, M.; Mohamed, F.; Parrinello, M.; Chassaing, T.; Hutter, J. Quickstep: Fast and accurate density functional calculations using a mixed Gaussian and plane waves approach. *Computer Physics Communications* **2005**, *167*, 103–128.

(38) Perdew, J. P.; Burke, K.; Ernzerhof, M. Generalized gradient approximation made simple. *Physical review letters* **1996**, *77*, 3865.

(39) Grimme, S.; Ehrlich, S.; Goerigk, L. Effect of the damping function in dispersion corrected density functional theory. *Journal of computational chemistry* **2011**, *32*, 1456–1465.

(40) Goedecker, S.; Teter, M.; Hutter, J. Separable dual-space Gaussian pseudopotentials. *Physical Review B* **1996**, *54*, 1703.

(41) Rappé, A. K.; Casewit, C. J.; Colwell, K.; Goddard III, W. A.; Skiff, W. M. UFF, a full

periodic table force field for molecular mechanics and molecular dynamics simulations. *Journal of the American chemical society* **1992**, *114*, 10024–10035.

(42) Potoff, J. J.; Siepmann, J. I. Vapor–liquid equilibria of mixtures containing alkanes, carbon dioxide, and nitrogen. *AIChE journal* **2001**, *47*, 1676–1682.

(43) Ongari, D.; Boyd, P. G.; Barthel, S.; Witman, M.; Haranczyk, M.; Smit, B. Accurate characterization of the pore volume in microporous crystalline materials. *Langmuir* **2017**, *33*, 14529–14538.
